# Identification of pleiotropy at the gene level between psychiatric disorders and related traits

**DOI:** 10.1038/s41398-021-01530-4

**Published:** 2021-07-29

**Authors:** Tatiana Polushina, Niladri Banerjee, Sudheer Giddaluru, Francesco Bettella, Thomas Espeseth, Astri J. Lundervold, Srdjan Djurovic, Sven Cichon, Per Hoffmann, Markus M. Nöthen, Vidar M. Steen, Ole A. Andreassen, Stéphanie Le Hellard

**Affiliations:** 1 NORMENT, Department of Clinical Science, University of Bergen, Bergen, Norway; 2 Dr. Einar Martens Research Group for Biological Psychiatry, Department of Medical Genetics, Haukeland University Hospital, Bergen, Norway; 3 NORMENT, Institute of Clinical Medicine, University of Oslo, Oslo, Norway; 4 NORMENT, Division of Mental Health and Addiction, Oslo University Hospital, Oslo, Norway; 5 Department of Psychology, University of Oslo, Oslo, Norway; 6 Department of Biological and Medical Psychology, University of Bergen, Bergen, Norway; 7 Department of Medical Genetics, Oslo University Hospital, Ullevål, Oslo, Norway; 8 Medical Genetics, Institute of Medical Genetics and Pathology, University Hospital Basel, Basel, Switzerland; 9 Department of Biomedicine, University of Basel, Basel, Switzerland; 10 Institute of Neuroscience and Medicine (INM-1), Research Center Juelich, Juelich, Germany; 11 Institute of Human Genetics, University of Bonn, School of Medicine & University Hospital Bonn, Bonn, Germany

**Keywords:** Genomics, Clinical genetics, Medical genetics

## Abstract

Major mental disorders are highly prevalent and make a substantial contribution to the global disease burden. It is known that mental disorders share clinical characteristics, and genome-wide association studies (GWASs) have recently provided evidence for shared genetic factors as well. Genetic overlaps are usually identified at the single-marker level. Here, we aimed to identify genetic overlaps at the gene level between 7 mental disorders (schizophrenia, autism spectrum disorder, major depressive disorder, anorexia nervosa, ADHD, bipolar disorder and anxiety), 8 brain morphometric traits, 2 cognitive traits (educational attainment and general cognitive function) and 9 personality traits (subjective well-being, depressive symptoms, neuroticism, extraversion, openness to experience, agreeableness and conscientiousness, children’s aggressive behaviour, loneliness) based on publicly available GWASs. We performed systematic conditional regression analyses to identify independent signals and select loci associated with more than one trait. We identified 48 genes containing independent markers associated with several traits (pleiotropy at the gene level). We also report 9 genes with different markers that show independent associations with single traits (allelic heterogeneity). This study demonstrates that mental disorders and related traits do show pleiotropy at the gene level as well as the single-marker level. The identification of these genes might be important for prioritizing further deep genotyping, functional studies, or drug targeting.

## Introduction

Many mental disorders share features such as clinical symptoms, cognitive deficits or drug prescriptions. This overlap has led to arguments for treating mental disorders along symptom spectrums rather than as separate diagnostic categories. The genetic architectures of many mental illnesses, cognitive phenotypes and brain morphology traits also display many general similarities as they are highly heritable and are influenced by many genetic variants of small effect [1–5]. Recently, studies have shown that mental disorders and associated traits also share genetic risk factors [6]. Statistical methods taking into account polygenic effects and gene-wide effects, linkage disequilibrium regression or empirical Bayesian models, have characterized specific genetic overlaps between many traits by using the effects observed in one trait to extract more information about the effects observed in another trait [6–11]. Indeed, genetic overlaps have been reported within the domain of mental disorders [9, 12] or between psychiatric and cognitive traits [13–15]. However, in these studies, the underlying assumption is that the genetic correlation between traits arises due to the presence of markers affecting both traits, i.e. pleiotropy at the SNP level.

There is a paucity of studies that have systematically used GWAS data, with the focus specifically at the gene level, to investigate the effects of independent variants on complex mental traits. In classical Mendelian diseases, it is well known that different pathogenic variants within one gene can be implicated in the same or a highly similar disease: e.g., numerous mutations within *CFTR* can cause cystic fibrosis [16–20], while several mutations in the *BRCA1* can increase the risk to develop breast cancer [21–23]. This phenomenon, called allelic heterogeneity, arises when different variants within a gene are independently associated with the same trait, usually because they all lead to similar pathogenic changes in protein function. Previously, we used schizophrenia (SCZ) GWAS summary statistics to identify several gene loci within which several independent markers were associated with SCZ [24, 25]. Going one step further, we here investigate pleiotropy at the gene level, which is defined as the association of several genetic variants in one locus with different traits. Many studies have investigated overlaps between mental disorders at the SNP level, but none to our knowledge have looked at genome-wide pleiotropy at the gene level. We obtained GWAS summary statistics for multiple mental disorders, cognitive functions, brain imaging traits and personality traits as relevant endophenotypes to identify pleiotropy at the gene level.

Cognitive deficits are consistently reported in many mental disorders such as SCZ, bipolar disorder (BPD) and autism [26, 27], and it has been suggested that measures of cognitive functions represent underlying phenotypes across different patient groups [28]. GWASs on educational attainment [29], general cognitive function (gF) [2, 30] and intelligence [31] have successfully identified genetic factors implicated in these traits. Genetic overlap has also been demonstrated between intelligence and SCZ and BPD [13], and between educational attainment and SCZ [14].

Regarding brain volume imaging traits, the ENIGMA consortium [32, 33], among others, has shown that patients with SCZ have smaller intracranial, hippocampus, amygdala, thalamus and accumbens volumes, but larger bilateral caudate, putamen, pallidum and lateral ventricle volume [34]. Genetic studies have also shown some polygenic overlap between SCZ and volumes of hippocampus, putamen and ICV; and between major depression and white matter integrity [35], although this was not found in all studies [36].

Personality traits may also influence behaviours and social functions [37]. For patients with mental disorders, such traits can be a risk factor (for example, neuroticism [38]) and can affect the clinical symptoms observed in the patients. Five-factor personality scales (neuroticism, openness, agreeableness, extraversion, conscientiousness) help to discriminate between the range of schizophrenia spectrum disorders [39]. Aggressive behaviour in early childhood may indicate behavioural problems and risk of mental disorders in adulthood [40, 41]. Furthermore, impaired social relationships and loneliness can lead to different psychiatric disorders, including personality disorders [42]. The genetic overlap between mental disorders and personality traits is indicated in various studies (e.g. neuroticism and SCZ [43]; or well-being and anxiety disorders [6]).

At the genetic level, the atlas of genetic correlation published by the BrainStorm consortium [6] reported genetic overlap between several of these traits at the single-marker level (SNP). They observed high correlation among attention deficit hyper-activity disorder (ADHD), BPD, major depressive disorder (MDD), and schizophrenia. The BrainStorm consortium also shows that some personality traits and cognitive traits correlated with mental disorders. However, the BrainStorm consortium did not examine the genetic overlaps at the gene level.

In the present study, we investigated genetic overlaps between mental disorders, brain morphometric traits and cognitive traits. We performed a systematic analysis of these GWASs to identify independent signals of association, using conditional regression [25], followed by comparative examination of certain genes and genomic regions which were associated with at least two different traits.

## Materials and Methods

Summary statistics from GWAS were obtained (see details in Supplementary Material) to test the three categories of phenotypes. For (i) mental disorders: we obtained GWAS from schizophrenia (SCZ) [1], autism spectrum disorder (ASD) [44], major depressive disorder (MDD) [45], anorexia nervosa [46–48], ADHD [49], BPD [50] and anxiety (Anxiety Neuro Genetics Study- ANGST) [51]; For (ii) brain morphometric traits: we obtained GWAS for subcortical brain volumes [33]; For (iii) cognitive traits: we obtained GWAS for educational attainment [29], general cognitive function (gF) [30]; and (iv) personality traits: subjective well-being, depressive symptoms, neuroticism [52], extraversion [53], openness to experience, agreeableness and conscientiousness [54], children’s aggressive behaviour [40] and loneliness [55]. All the GWASs selected were performed on a sample of European origin.

The pipeline of analyses consisted of several steps which are described in detail below. Briefly, the pipeline, described in Supplementary Fig. 1, consisted of the following steps: (1) Perform unified quality control for all the GWAS obtained from different databases, (2) In each GWAS identify independent signal of association with conditional regression using cojo_GCTA, from which we derive a list of all genomic regions associated in all GWAS, (3). Annotation of the associations to genes or group of genes (if some association were mapping to genomic regions with more than one gene); (4) Identification of genes and gene groups with more than one association; (5) Classification of the regions with more than one signal in three possible scenarii which corresponds to either allelic heterogeneity (one trait several association), marker-based pleiotropy (same marker associated with different traits) or gene-based pleiotropy (different markers in the same gene/gene group associated with several traits). For the gene and gene groups identified in step 3, we also perform an analysis based on gene score calculated for each trait, taking all the SNPs located in the gene, which were then compared across traits.

### Quality control of GWAS summary statistics

The GWAS datasets, including SNPs and sample numbers, are described in the Supplementary Material. For all the studies, we performed the same quality control (QC) procedure based on the summary statistics obtained for the different GWASs (see Supplementary Table 1). The QC parameters were selected based on recommendation for using the cojo-GCTA method [25]: poorly-imputed SNPs with imputation score < 0.9, ambiguous SNPs, markers with minor allele frequencies < 0.1 and insertions/deletions were filtered out during our QC. Genome mapping was based on the human genome reference hg19. In studies using hg18 coordinates, conversion to hg19 was conducted using the UCSC Genome Browser liftOver tool [56]. Statistics for chromosome X were available only for the SCZ GWAS. We thus focused only on autosomal chromosomes.

All sample collections were approved by the relevant local ethic committee and all individuals provided informed consent.

### Selection of independent signals

For each trait, independent signals of association were selected by conditional stepwise regression using the cojo-GCTA tool [25]. We and others have shown previously that cojo-GCTA is better than LD pruning for identifying independent and conjunctional association signals in summary statistics [24, 25]. Input data comprised (a) summary statistics, filtered after performing stringent QC procedures – checking effect sizes, standard error, *p*-value and allele frequency for each GWAS; and (b) a sample of Norwegian origin and a sample of German with 4678 individuals with 7,111,231 genotyyped and imputed markers, according to cojo-GCTA protocol (for details see [24, 25]). We previously demonstrated that a cojo-GCTA threshold of 10^−5^ is reliable to identify independent signals.

### Gene and genomic region binning

Selected independent SNPs were annotated to 26,025 known unique RefSeq genes (1.02.2017 freeze) [57]. In the RefSeq library all isoforms of a gene are mapped to one unique RefSeq gene. The LDsnpR tool [58] was used to assign signals to genes if they were located within the boundaries of a RefSeq gene, (±10 kb). Genes that were fully overlapping or antisense and which thus contained the same combination of annotated markers were merged into gene_blocks. Gene_blocks were defined as genomic regions containing several genes where the boundaries of the block correspond to the minimum start position and the maximum end position among the overlapping genes included in the block. The SNPs were assigned to a total of 1410 genes.

### Identifying SNP-based allelic heterogeneity and pleiotropy within and across traits

A total of 226 gene-groups (with 242 genes) contained more than 2 association signals identified by cojo-GCTA (*p* < 1 × 10^−5^), 177,344 SNPs were mapped to the selected 226 regions. Using the pair-wise SNP pruning procedure from PLINK [59] with a 10 Mb window and *r*^2^ = 0.2 and the same LD reference as described early, these 177,344 SNPs were assigned to 8772 independent markers. Since our analyses are focusing on these regions, the experiment-wide significance threshold at 5% with Bonferroni correction was set to 0.05/8772 = 5.70 × 10^−6^.

### Identifying gene-based allelic heterogeneity and pleiotropy within and across traits

All the SNPs mapped to the genes were used to calculate a gene score for each gene and each trait using the Brown method [60, 61]. Brown scores were calculated for all 1410 genes which contain at least one SNP identified by cojo-GCTA, all SNPs in these genes were included to calculate the Brown score, including 10 kb flanking regions. The LD corrections were based on the LD reference described earlier (i.e. our German and Norwegian genotyped sample). The scores were corrected for multiple testing using the false discovery rate (FDR) method within a trait. The threshold for the FDR-adjusted gene score was 0.05/27 phenotypes tested = 0.0018 for association across phenotypes. A total of 379 genes passed this threshold for at least two traits. We further filtered these genes by excluding those minimal *p*-values > 5.70 × 10^−6^ (study-wide threshold of association).

### Haplotype analysis

For the purpose identify potential haplotype association in independent association, we scrutinize haplotype frequencies for one example: *MAN2A1* (chr5: 109.03–109.22 Mb). Frequencies of haplotypes were estimated using the German and Norwegian reference samples in Haploview [62]. Haploblocks were defined with the four gamete rule in Haploview [62] with *r*^2^ threshold 0.8 and window size 500 kb. We examined only haplotypes with frequencies higher 10%. We also examine the haplotypes for SNPs with *r*^2^ > 0.8 with rs4388249; (2) and SNPs with *r*^2^ > 0.8 with rs1368357; SNPs cluster were derived using Plink [59] from Norwegian + German reference set.

## Results

### Identification of independent association signals and corresponding gene loci

The analysis pipeline is shown in Supplementary Fig. 1. We selected 7 GWASs for mental disorders, together with 20 GWASs for cognitive, personality and brain morphology traits that have been suggested as potential endophenotypes for those mental disorders [30, 33, 34, 40, 63, 64], or have some genetic overlap with those mental disorders. Using the summary statistics from all 27 GWASs, we identified a total of 2190 unique associated SNPs after conditional regression [25].

### Gene annotations

Out of 2190 unique SNPs, 775 could not be annotated to any gene, whereas 1415 SNPs were annotated to 1410 genes. If two or more genes had overlapping SNPs, we merged them into gene_blocks. This resulted in the identification of 1161 genes or gene-groups (Supplementary Fig. 1). We identify genetic overlaps either by selecting genes based on the single SNP minimal p-value, which we call SNP-based analysis, or by selecting genes with a Brown score lower than the study wise FDR level.

### Genetic overlaps at the gene level and SNP level within or between traits

**Fig. 1:**
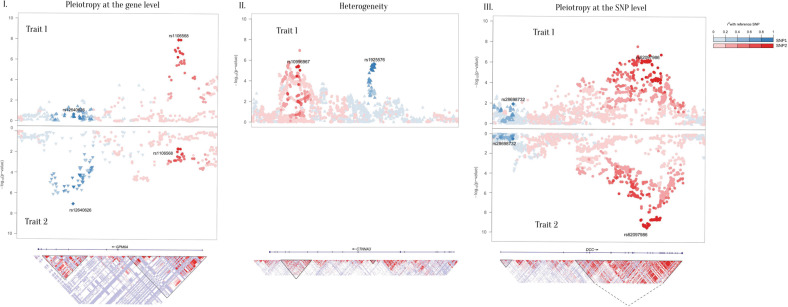
Three possible scenarios for genes harbouring multiple association signals. Upper panels: regional association plots illustrating the three different scenarios. Scenario I, Pleiotropy at the gene level: two LD-independent clusters within the gene *GPM6A* are associated with SCZ (red cluster, trait 1) and education (blue cluster, trait 2). Scenario II, Allelic heterogeneity: within one gene (*CTNNA3*), two LD-independent clusters are associated with one trait (Education). Scenario III, Pleiotropy at the SNP level: within the gene *DCC*, one cluster of LD-dependent genetic variants is associated with both depression (trait 1) and putamen volume (trait 2). Red and blue colours correspond to the strength of LD with the first and second tagged SNPs. Figures were plotted using the LocusZoom tool [90]. Lower panels: underlying LD pattern for each region. Figures were obtained using the UCSC Genome Browser [56] with CEU population as reference and *LOD* values plotted.

Out of the 1161 gene groups, 226 displayed significant association to at least two different traits (Table 1). Loci with *p*-values below the experiment-wide threshold of 5.70 × 10^−6^ were selected for further analyses. We observed three possible scenarios (see Fig. 1):

- Scenario I. Pleiotropy at the gene level: genes or gene-groups with multiple associations to LD-independent genetic variants for at least two traits (*N* = 25).
- Scenario II. Allelic heterogeneity within a trait: genes or gene-groups with multiple associations to LD-independent genetic variants and with only one trait (*N* = 9).
- Scenario III. Pleiotropy at the SNP level: regions with multiple associations to LD-dependent genetic variants and with more than one trait (*N* = 47).

**Table 1:** Number of gene/gene-groups containing more than one marker associated after cojo-GCTA at different significance threshold (genome-wide significance threshold 5 x 10^−8^, experiment-wide threshold 5.7 x 10^−6^ and recommended threshold for cojo-GCTA 1 × 10^−5^).

	2+ markers < 5 × 10^−8^	2+ markers < 5.7 × 10^−6^	2+ markers < 1 × 10^−5^	Scenario
*Markers independent (not in LD)*				
One trait	2	9	27	II
Several traits	0	25	96	I
*Markers dependent (in LD)*				
Several traits	15	47	79	III
*Mixed*				
Several traits	5	24^a^	24	

^a^Includes 3 gene groups with the same SNP selected in different traits.

A locus was considered to contain dependent markers if at least one pair of SNPs that were associated to traits had *r*^2^ ≥ 0.2 (Table 1, based on the European-descent individuals (EUR) from the 1000 Genomes [65]).

#### Scenario I: Pleiotropy at the gene level

In this scenario, we observed two situations: (i) several LD blocks were present within a gene or a gene-group, and SNPs from different LD blocks were associated with different traits; or (ii) positionally overlapping sets of SNPs not in LD were associated with different traits (Supplementary Table 2, Supplementary Fig. 2; these genes can also be visualized at https://genlocus.shinyapps.io/2locus/).

Twenty-five genes displayed independent associations with two traits: *RERE*, LOC102724552, *GPM6A* (SCZ and educational attainment); *RBKS* (SCZ and BPD); *DLGAP2* and *ERICH1-AS1* (SCZ and ADHD); *GLIS3* (SCZ and neuroticism); *JADE2* (educational attainment and BPD); *ASTN2* (ASD and BPD); *FHIT* (SCZ and MDD); *TEAD1* (educational attainment and subjective well-being); *TENM4* and *MACTOD2* (gF and BPD); *EXT1* (gF and AUT); *LRRC4C* (gF and anorexia); *CACNA1E* (gF and Neuroticism); *CTNNA2, LRP1B, ATXN1, SNX29, CDH2* (educational attainment and gF); *ZNF385B, MIPEPP3, PHACTR3* (gF and SCZ). In addition, *RBFOX1* has two independent association signals with both SCZ and gF, giving four independent hits within one gene.

#### Scenario II: Allelic heterogeneity within a trait

We observed 9 genes in which several independent signals are associated with one trait (Supplementary Table 3, Supplementary Fig. 3): *BSN* and *CTNNA3* for educational attainment, *RPS6KA2* for BPD, *NKAIN2* and *CDKAL1* for gF. For Alzheimer’s disease, allelic heterogeneity was observed in the genomic locus containing *NECTIN2*, *TOMM40*, *APOE* and *APOC1*, which has been reported before [66, 67] and reflects the fact that different *APOE* haplotypes ε2, ε3 or ε4 can be risk factors or protective factors for Alzheimer’s disease [68–71].

### Scenario III: Pleiotropy at the SNP level

We identified 47 loci with 53 genes where the same SNP, or SNPs in LD (r^2^ ≥ 0.2), were associated with different traits at the study-wide level. For most of the genes, a pair of markers in LD were linked to two traits. However, for *DCC* and *STK24*, 5 and 4 SNPs were associated with 6 and 4 traits respectively (see Supplementary Table 4, Supplementary Fig. 4). Typically, Scenario III overlaps should also be identified in studies that investigate genetic overlap at the single-marker level [7–9]. Since not all the genetic overlaps that we have tested here have been investigated using marker-based pleiotropy, we could not compare systematically whether the single-marker pleiotropy we identified is actually reported with other methods. We compared with one published study for overlap of schizophrenia and education at the marker level [14], 8 of the 10 markers identified in the scenario III had been identified previously, the two additional being probably due to different significance thresholds between the studies.

#### Mixed cases

are loci that contain both LD-dependent and -independent markers which are associated with different traits; e.g. genes with allelic heterogeneity for one trait and an association of one of these SNPs with another trait (see Supplementary Table 5, Supplementary Fig. 5). For example, *CACNA1C* has two sets of SNPs which are associated with SCZ, while rs10744560 is also associated with BPD (Supplementary Fig. 5R). Some of these overlaps at the SNP level have been identified previously: *ZEB2* [14], *JMJD1C* [10, 72], *LINC00461*; *EXOC4* and *SORCS3* [13].

### Gene score-based pleiotropy

To explore the combined effect of markers within a gene, we also examined association at the gene level by obtaining gene-based scores. For each gene, we calculated Brown scores [61] across traits and filtered those for association at the study-wide level. We identified 23 additional genes with pleiotropy at the gene level across traits (see Table 2 and Supplementary Fig. 6). The pleiotropic genes were: *LRRN2, MAN2A1* and *EYS* (SCZ and educational attainment); *COL16A1, EFNA5*, *SHANK3* (educational attainment and gF); *BRE-AS1* and *LINC01378* (SCZ and BPD); *CLU*, *MIR6843* (SCZ and Alzheimer’s disease); *SFXN5, SATB2, FXP1, CKB, TRMT61A, APOPT1*, (SCZ and gF); *TEX41* (ADHD and educational attainment); *ZMIZ2, TMEM245 and SLCO3A1* (educational attainment and gF); *KCNC2*, (educational attainment and neuroticism); *CDH8* (ADHD and gF); *TCF4* (SCZ and neuroticism). One of these 23 genes, *SHANK3*, had been reported in another study [10, 72].

**Table 2:** Number of genes identified by gene-based analysis.

Criterion		# of genes
Genes significantly associated with ≥2 traits		379
Genes identified with marker-based analysis		69
Not identified by SNP-based analysis	Marginal SNP p-values do not pass threshold 5.7 e-6	135
	LD-independent markers (scenario I)	23
	LD-dependent markers (scenario III)	104
	Mixed	48

We also report 104 additional genes with LD-linked markers (Supplementary Table 4) and 48 genes with a mixed case scenario (Supplementary Table 5).

An overview of the genes identified with gene-based pleiotropy is presented in Supplementary Table 6 with a description of their functions and annotation to additional relevant clinical and experimental information such as mouse models, or involvement in mental retardation, or other evidence for involvement in mental disorders, such as the presence of rare variants and CNVs. Rare variants and CNVs in 11 of the 48 genes have been implicated in intellectual disabilities or mental retardation, while rare variants and CNVs in 5 of these genes have been associated with ASD or SCZ.

### Haplotype association

**Fig. 2:**
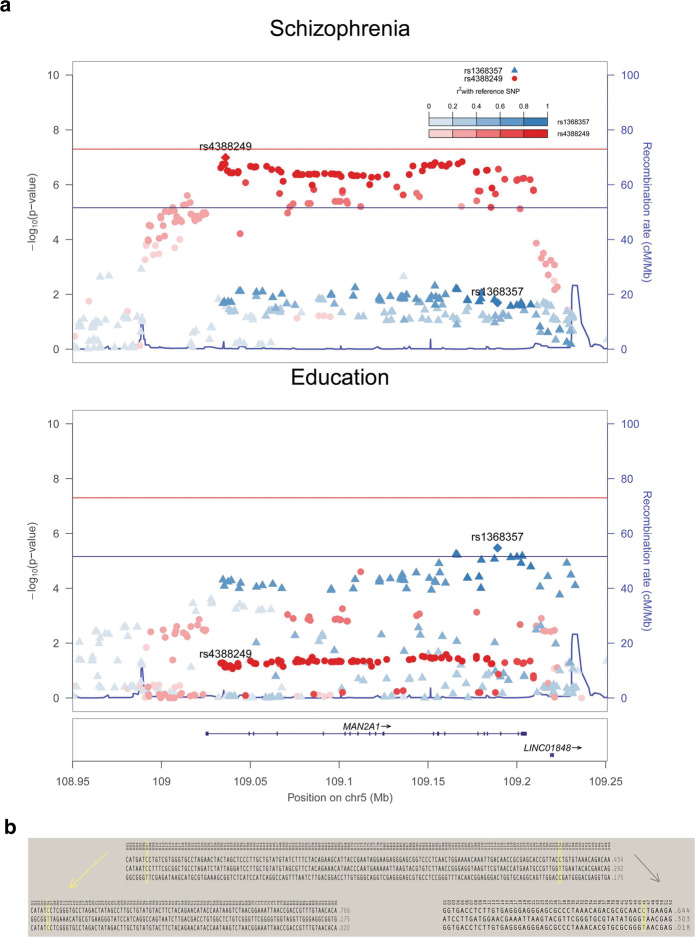
Regional plots and haploblocks for the *MAN2A1* gene. **a** Regional plots for association with schizophrenia and educational attainment. The markers are labelled according to their LD with the reference SNPs. Red colours correspond to LD values between the markers and rs4388249; blue colours correspond to LD values between the markers and rs1368357. **b** Haplotypes for the region (from the Norwegian+German cohort described above sample [91]). In the upper plot, all the SNPs are displayed. Three haplotypes have a frequency >10% (top) and the SNPs rs4388249 (number 7) and rs1368357 (number 134) are highlighted in yellow (2B1). The risk allele for SCZ, rs4388249_T, is present on the 3^rd^ haplotype, while the allele associated with higher education, rs1368357_T, is present on the 2^nd^ haplotype. The lower plots represent the LD block split into the LD cluster for rs4388249 (2B2), and LD cluster for rs1368357 (2B3), which shows how the sets of clusters are in high LD and overlap within the same block.

We further explored the 23 genes that demonstrated genetic overlap across traits in the gene score-based analysis. We observed that in most cases there were two sets of markers that positionally overlapped within an LD block defined by two groups of markers in high r^2^ LD (r^2^ > 0.8). Since this could reflect an association to different haplotypes, we performed haplotype analyses within haploview [73] and, as an example, we display the haplotype analysis for *MAN2A1* (chr5: 109.03–109.22 Mb, see Fig. 2a). The genotypes from the German and Norwegian samples were segregated into haplotypes within haploview [73]. In this region, the SNP rs4388249 which is associated with SCZ (p = 3.05 × 10^−8^, with the T allele being increased in cases, see Supplementary Table 7), is independent of the SNP rs1368357 (i.e. r^2^ < 0.2 with rs4388249) which is associated with educational attainment (p = 3.37 × 10^−6^, with T allele being associated with lower education, see Supplementary Table 7). At the haplotype level we observed that the T allele of rs4838249 is located on one haplotype, while the T allele for rs1368357 is located on another haplotype. Thus, the alleles that are associated with one or the other phenotypes are located on different haplotypes (see Fig. 2b). While we did not perform such deep screening of these associations we can expect that other association to independent signal will also reflect association at the haplotype level.

### Comparison between genetic overlaps at the gene level and at the SNP level

As the final step, we compared the genetic overlap between traits when considering either the entire gene or the SNPs as the candidate loci. The upper triangle of Supplementary Fig. 7 represents genes where independent SNPs were associated with pairs of traits, while the lower triangle represents genes in which the same SNP (or SNPs in LD with r^2^ > 0.2) was associated with pairs of traits. We observed a higher number of overlaps for the pairs SCZ/gF, SCZ/educational attainment, SCZ/BPD, and SCZ/neuroticism. This might reflect a higher genetic overlap between these traits, but it may also partly be due to the fact that SCZ, gF and educational attainment are the studies with the largest sample sizes, and hence have the highest power to detect association.

For most of the trait pairs, there is more pleiotropy at the SNP level than at the gene level, but for a few pairs, some pleiotropy is observed at the gene level that is not observed at the SNP level.

## Discussion

In this study, we have identified genes which harbour multiple associations with a spectrum of mental disorders and other phenotypes that have been suggested to act as endophenotypes. We observed either pleiotropy between different traits at the gene level, pleiotropy at the SNP level, or allelic heterogeneity within a trait for one positional locus. Overall, we observed more pleiotropy at the SNP level (scenario III) then at the gene level. We also observe more overlap for group of cognitive and personality traits and their overlap with group of psychiatric traits and brain volume traits.

There were 48 genes with at least one pair of independent markers associated with at least two traits, i.e. pleiotropy at the gene level which has not been reported before. We also report 157 genes where the pleiotropy was at the SNP level most of which have been identified by classical pleiotropy analyses.

The variety of genetic overlaps that was observed reflects the complexity of the genomic landscape (genetic architecture and LD maps). Several genes show clear pleiotropy at the gene level. This provides important information on novel specific genes that have been implicated in certain disorder but which have not been systematically reported as genetically overlapping across traits. This is probably because they do not overlap at the marker level and hence are not identified in the classical pleiotropy studies reported so far for psychiatric disorders. While the pleiotropy at the SNP level between psychiatric traits is well established [7, 9, 13, 15], and can be used to characterize more genetic architecture and functional mechanisms in mental disorders, the current findings show that it is also important to consider the pleiotropy at the gene level for further functional studies.

In Supplementary Table 6, we present the biological functions and relevance of all genes with pleiotropy at the gene level. An example is the gene *CLU*, which is the third most significant genetic risk factor for the development of late-onset Alzheimer’s disease [74, 75]. It encodes the clusterin/apolipoprotein J which has been shown to alter the aggregation and toxicity of Aβ peptides and promote their clearance across the brain-blood barrier [76, 77]. *CLU* has also been previously associated with SCZ [1, 78], we now show that these two associations are independent as the two signals of association are not in LD (r^2^ < 0.2). In some studies it was suggested that oxidative stress, neuroinflammation, neurovascular endotheliopathy and disruption of the brain-blood barrier may lead to the cognitive and behavioural symptoms of SCZ via several processes including interactions between brain-innate and peripheral adaptive immunity [79].

There are many possible explanations for the independent associations. We observed that the independent associations could be due to either association in different parts of a gene or to different genes within a gene group, but they can also be due to different haplotypes within the same part of the gene. We have detailed in the results how further scrutiny of such overlap can show which of these signals are due to association with different haplotypes on the *MAN2A1* gene. In a quick overview of these associations, we identified several other potential haplotype association, but this would warrant haplotype analyses for each of them which we do not present here. In the case of haplotype association, it is possible that sets of alleles on one haplotypes are actually in linkage with other variants that are rare or imperfectly tagged by the set of markers typed in the GWAS. It has been speculated that some GWAS associations are due to so-called synthetic association to rare variants that are imperfectly tagged in GWASs [80, 81]. Capturing these variants at the genome level is challenging, but the genes we identified here are potential candidates to investigate for such synthetic rare variants. Thus, it would be efficient to prioritize these genes for deep genotyping or sequencing. One could also select individuals for deep sequencing based on their haplotypes in order to increase the chances of identifying such synthetic variants.

Some of the signals that we report could be due to rare variants that cannot be identified by GWAS, but it is also possible that the pleiotropy at the gene level reflects either association with different isoforms of the genes or association with different functional variants in the gene, especially if the associations are not overlapping. For instance, we report pleiotropy for the *RBFOX1* gene which is known to be mainly expressed in the brain (Supplementary Table 6) and to have a causal role in autism spectrum disorder [82]. There are five described and twelve potential isoforms for *RBFOX1*, and two of them (Rbfox1_N and Rbfox1_C) regulate two different sets of genes that are involved in the transmission of nerve impulses and synaptic transmission, but have quite different targets and exemplify the functional consequences of alternative splicing for this RNA binding protein [83]. In future studies, it would be interesting to finely map which isoforms or functional variants are associated with the different independent associations.

While performing analyses to identify pleiotropy at the gene level we also identified allelic heterogeneity, i.e. several independent associations to the same trait within a genomic locus. Recently, allelic heterogeneity has been reported more often in GWASs, as a result of using tools in the data analysis pipeline such as conditional analysis or pruning to check the number of independent signals in the GWAS (e.g. coronary artery disease [84], educational attainment [29], and schizophrenia [24, 85]). In Mendelian genetic studies, allelic heterogeneity has been demonstrated for the *BRCA1/BRCA2* genes, and it has been shown that the different mutations can be associated with different penetrance, presentation and severity of the symptoms [21–23]. In an example of a mixed case, one trait (SCZ) [1] had allelic heterogeneity associations with the *CACNA1C* locus, and one of these associations overlapped with BPD [50].

Several of the genes in which we identified pleiotropy are some of the largest genes in the genome (e.g. *RBFOX1, MACROD2, LRP1B*, etc). The median size of genes with pleiotropy at the gene level identified in this study is 95 kb, which is higher than the average gene (ca. 15 kb). However, this could be expected since genes associated with mental disorders tend to be big. For comparison, the median size of the genes that were identified at the step of conditional regression (the 1401 genes) is 65 kb. Thus, there is a slight increase of representation of larger genes in the ones showing pleiotropy. This increase might represent a statistical bias but it is also expected that the larger the genes, the higher will be the chance that variants will arise in the genes that could influence traits in a pleiotropic manner and therefore gene-based pleiotropy should arise more often in larger genes. In addition, larger genes tend to have more transcript variants which also can have different associations to different traits and therefore show more often pleiotropy at the gene level.

By conducting a literature review for the genes where we report gene-based pleiotropy, we found evidence that for 19 of these 43 genes or gene-groups, rare variants, microdeletion or CNV have been identified for intellectual disabilities, mental retardation or other psychiatric traits (see Supplementary table 6 for more details). However, there is no existing method or database to evaluate how significant this is. Our study was limited to testing overlap between GWASs, but it would certainly deliver even more evidence to implicate genes if we could systematically incorporate several layers of genetic evidence across related phenotypes, ranging from GWAS of complex traits to single-nucleotide variants in Mendelian traits or syndromes. Therefore, it is important to carry out more systematic annotation of different sources of evidence to uncover specific genes that are involved across psychiatric disorders. It is especially relevant to identify genes that are common to several traits and which could be the best target of new drugs.

### Limitations

We performed our analysis based on publicly available summary statistics from different GWAS consortiums. Thus, when we observed LD-linked signals, it is possible that what we identified as a pleiotropy scenario may instead be due to overlapping cohorts (within the meta-analysis). This is indeed a usual concern in analyses when samples are combined, which can induce a bias in over-representation of some populations. However, here we do not combine samples, but we compare them and try to reduce the overlap where it was possible. That overlap is especially relevant within the cohorts used for the analysis of brain volume traits and cognitive and personality traits (Supplementary Figs. 8, 9). We tested overlapping signals across the traits from ENIGMA, which used the same cohort for each phenotype, and noticed low overlap across the eight ENIGMA GWASs. Still, we observed several overlaps at the gene level between traits that share many cohorts, e.g. gF and educational attainment. Therefore, we should be cautious about calling these overlaps as independent for these studies since the “independent” signals might just be an artefact due to imperfect tagging that would have different LD in the two different meta-analyses and our reference data. In order to reduce genetic overlap, we excluded overlapping cohorts from the summary statistics, where it was possible to obtain data, such as the summary statistics obtained from brain volumes do not have any overlap with the PGC data for mental disorders and have only a minor overlap with the CHARGE data.

We had to limit our annotations to genes since we do not currently have robust systematic methods to perform annotation based on regulatory regions. It is well documented that in some cases the associations appear to be located within one gene, but they are actually due to effects on the regulation of a nearby gene [86]. As an example, the gene *GLIS3*, which has been implicated in diabetes and does not seem to have a major role in the brain, is actually located next to *SLC1A1* (encoding the high-affinity glutamate transporter) which has been associated with obsessive-compulsive disorder and psychosis [87, 88].

In addition, we should emphasize that we were stricter with gene-based analysis and performed two steps correction, which may have an effect on the observed lower number of genes with pleiotropy at the gene level than at the SNP level and thus the higher pleiotropy observed in the later might be due to differences in significance thresholds.

Another limitation in our study is due to the disparity in the sample size for the different GWAS that we tested as well as the difference in trait heritability which both most likely affect the power to identify the different pleiotropy in the traits. This is for instance reflected in the observation that we could identify more pleiotropy for the biggest GWAS (e.g. schizophrenia) while we could not identify pleiotropy in some of the smaller ones (e.g. brain structure traits). So our results must be interpreted with that in mind and these analyses will need to be repeated as the data available for different GWAS will grow.

Finally, another limitation in the study design is that for the cojo-GCTA step we used Norwegian and German merged sample as a representation of mixed European population structure. It is a population proxy that should reflect well the population structures of cohorts included into consortium meta-analysis (since it overlaps with most), still as a proxy could bring some noise into initial signal selection and affect on detection of haplotypes.

## Conclusions

Pleiotropy at the SNP level has previously been demonstrated between psychiatric disorders. In this study, we have identified genes with at least two independent associations to different traits, i.e. gene-based pleiotropy. This type of analysis brings additional information to implicate certain genes across different disorders. This is especially crucial when considering functional analyses of candidate genes, or when considering which genes should be used as drug targets. For instance, we show here that several genes implicated in SCZ are also implicated in Alzheimer’s disease. It will be crucial to understand this relationship in order to avoid targeting genetic pathways in SCZ that could increase the risk of developing Alzheimer’s disease. We also identified several genes which would require deep sequencing in order to identify rare variants or to determine if the independent signals are due to different functional variants.

## Supplementary information

Supplementary Figure 1

Supplementary Material

Supplementary Figure 2

Supplementary Figure 3

Supplementary Figure 4

Supplementary Figure 5

Supplementary Figure 6

Supplementary Figure 7

Supplementary Figure 8

Supplementary Figure 9

Supplementary Table 1

Supplementary Table 2

Supplementary Table 3

Supplementary Table 4

Supplementary Table 5

Supplementary Table 6

Supplementary Table 7

## Data Availability

The following analysis programs were used: GCTA [25]; PLINK [59]; LDSnpR [58]; and Haploview [89]. For plotting, we used LocusZoom [90] and the R-package gplots. All the tools are publicly available. Brown score was calculated with –set-screen flag in PLINK [59].
