# Supplementary figures and images for: Identification of pleiotropy at the gene level between psychiatric disorders and related traits
Source: Transl Psychiatry. 2021 Jul 29;11:410. doi: 10.1038/s41398-021-01530-4 (PMC8322263; doi:10.1038/s41398-021-01530-4)

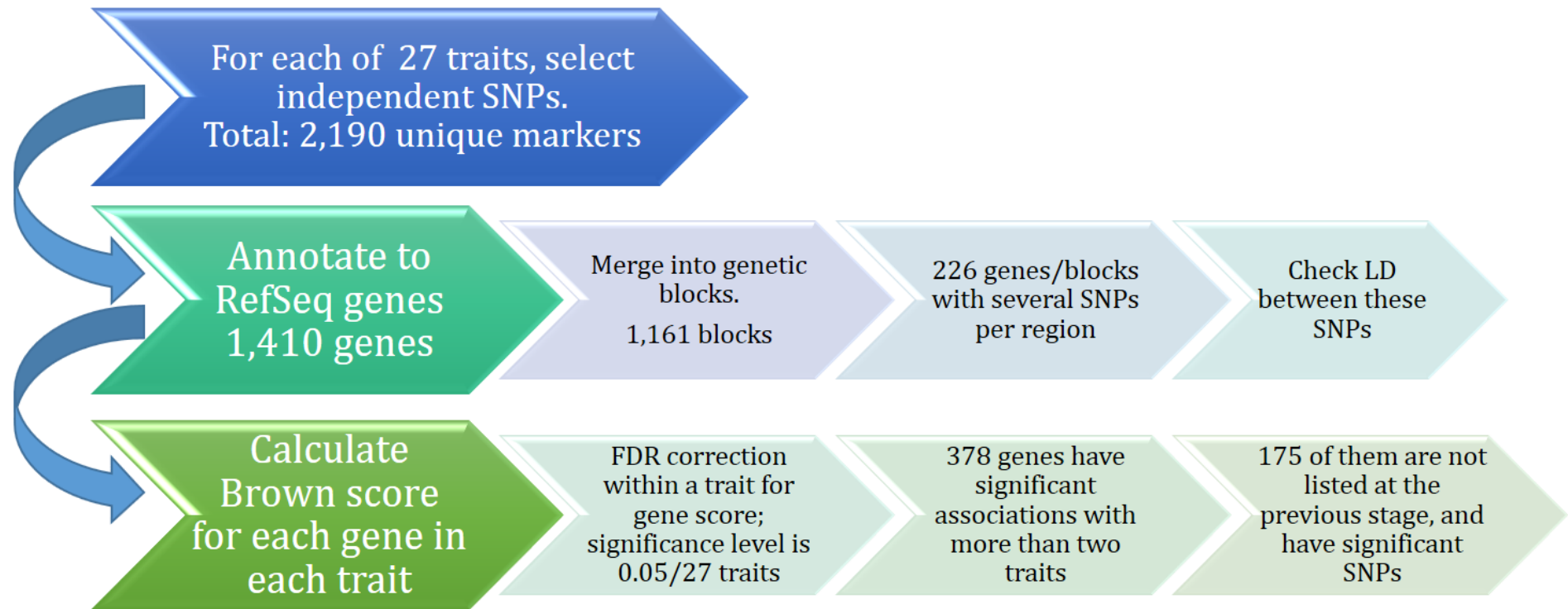

Supplement: Supplementary file 1 — Supplementary Figure 1 [file 41398_2021_1530_MOESM1_ESM.pdf]

## Education

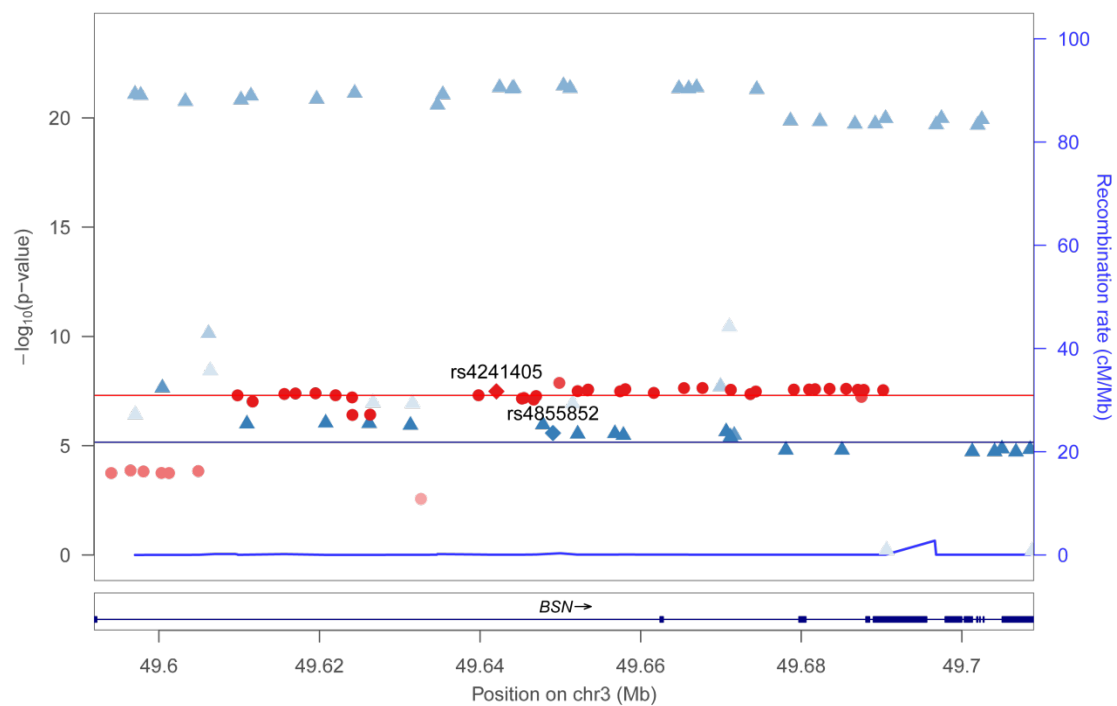

A.

## gF

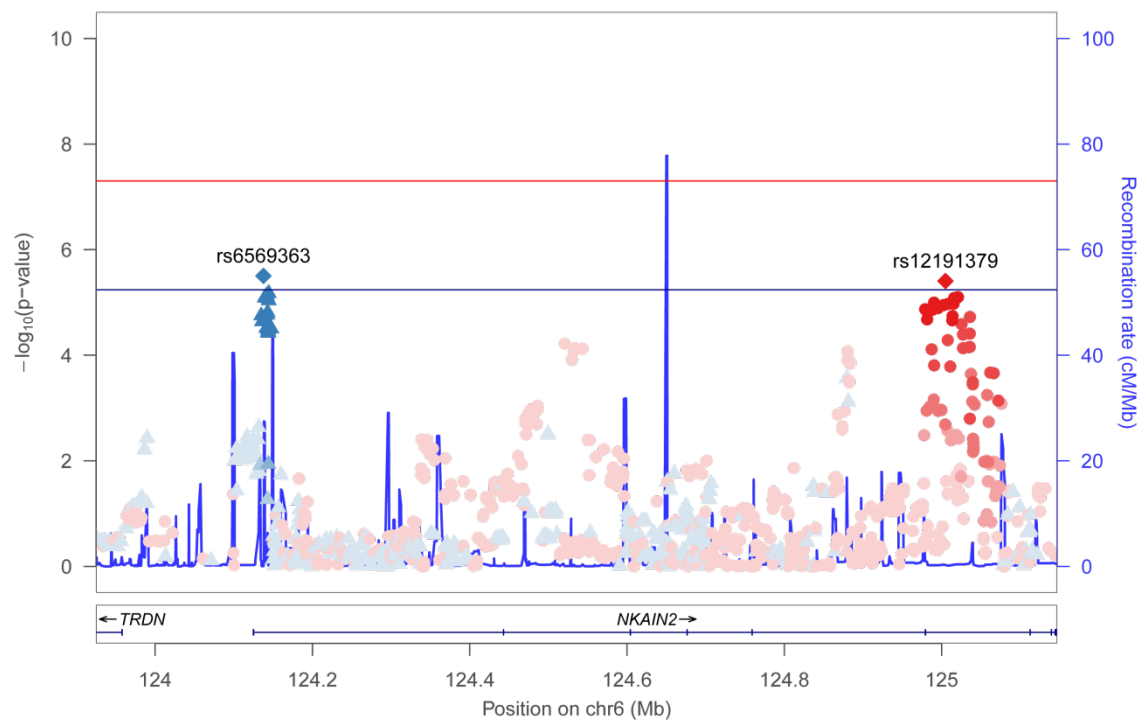

B.

## Bipolar disorder

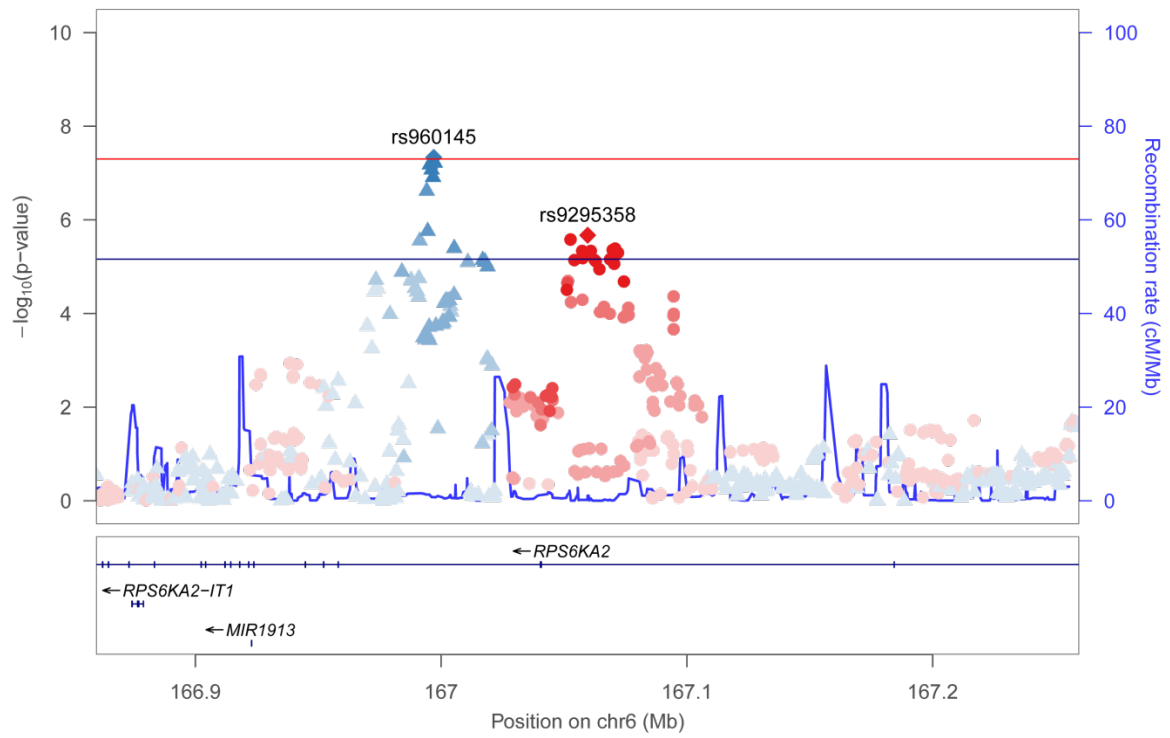

C.

## gF GWAS

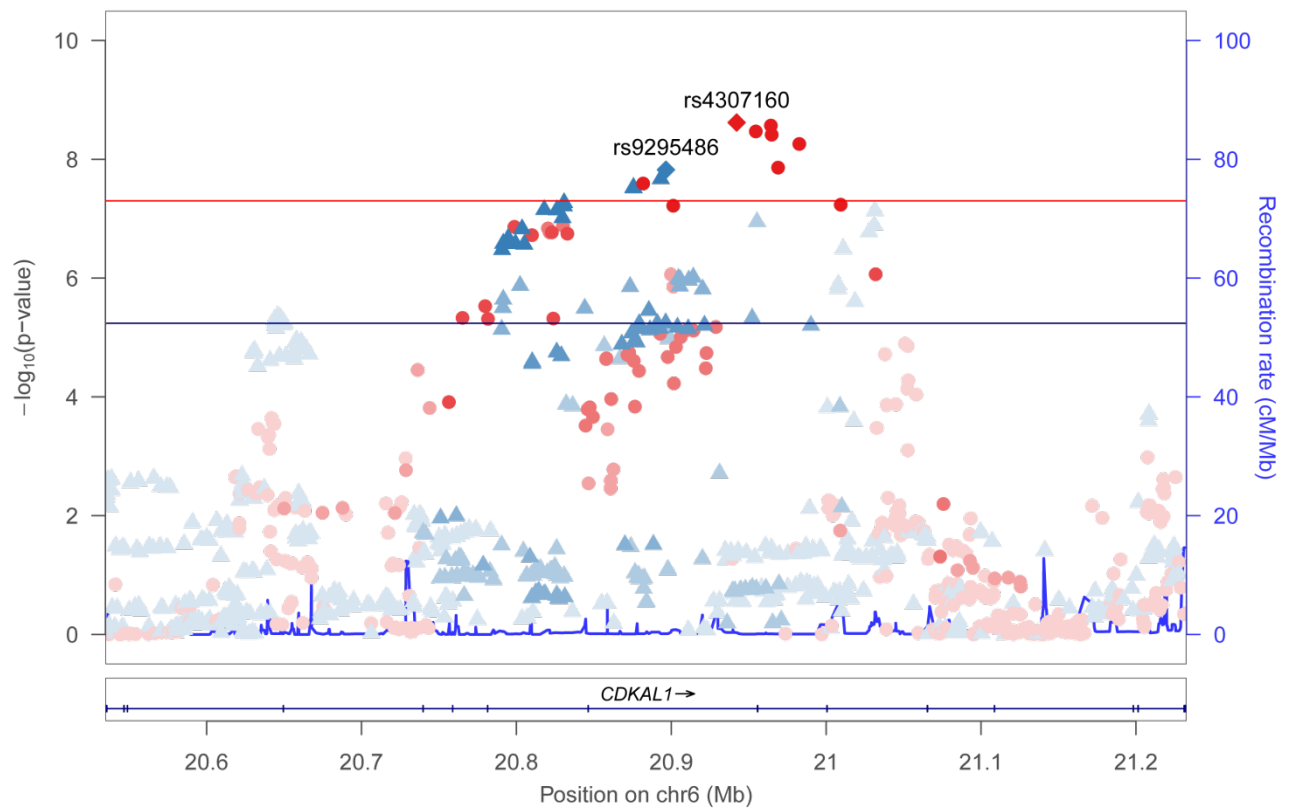

D.

## Education

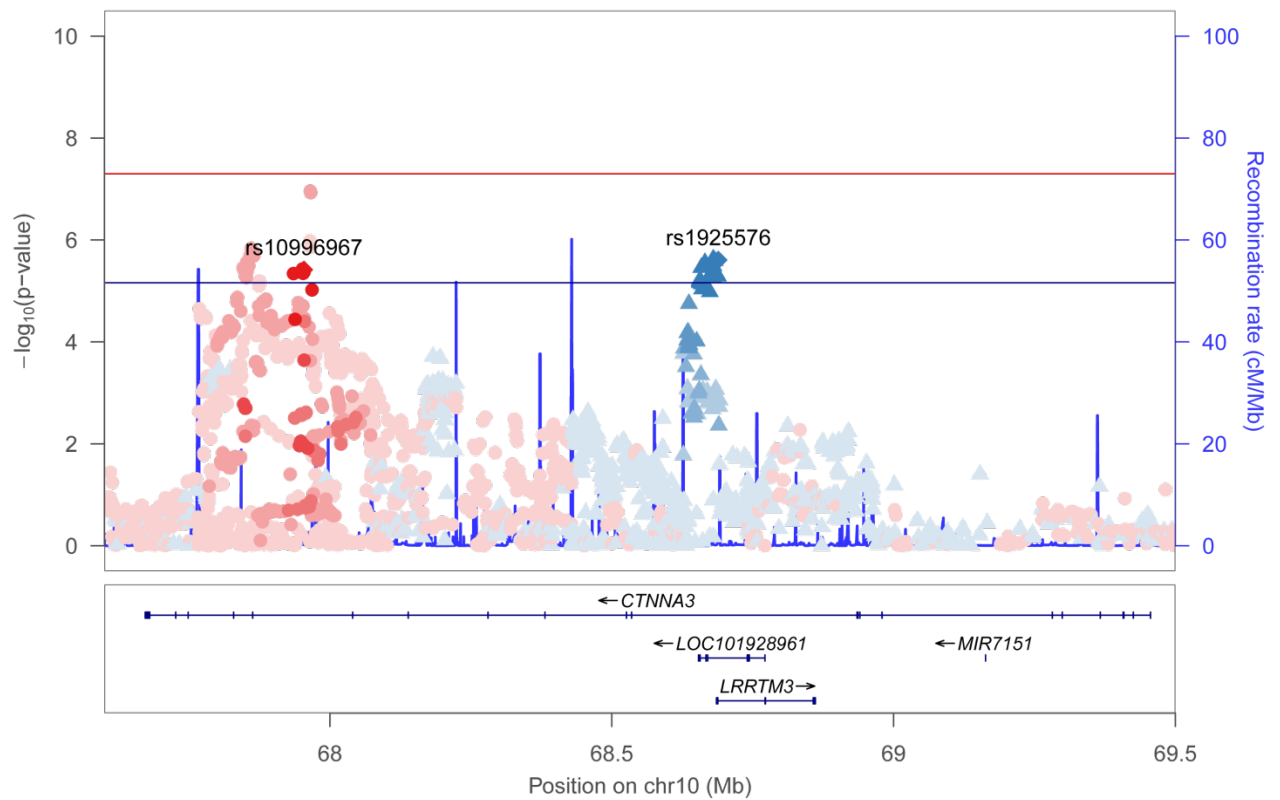

E.

## Alzheimer

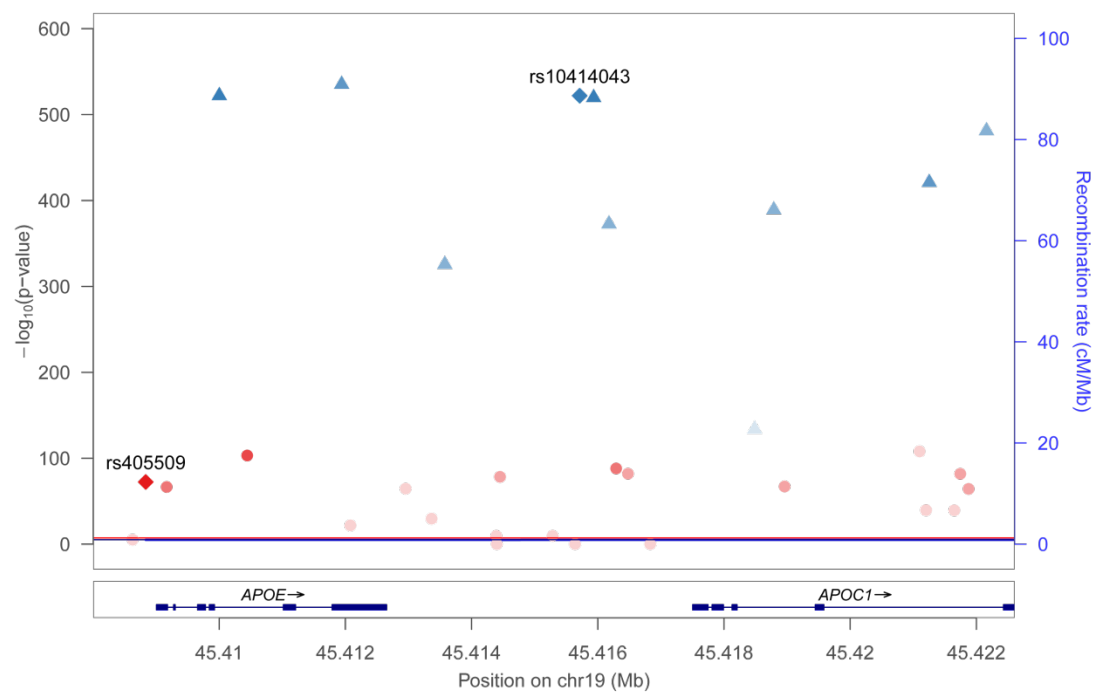

F.

Supplement: Supplementary file 4 — Supplementary Figure 3 [file 41398_2021_1530_MOESM4_ESM.pdf]

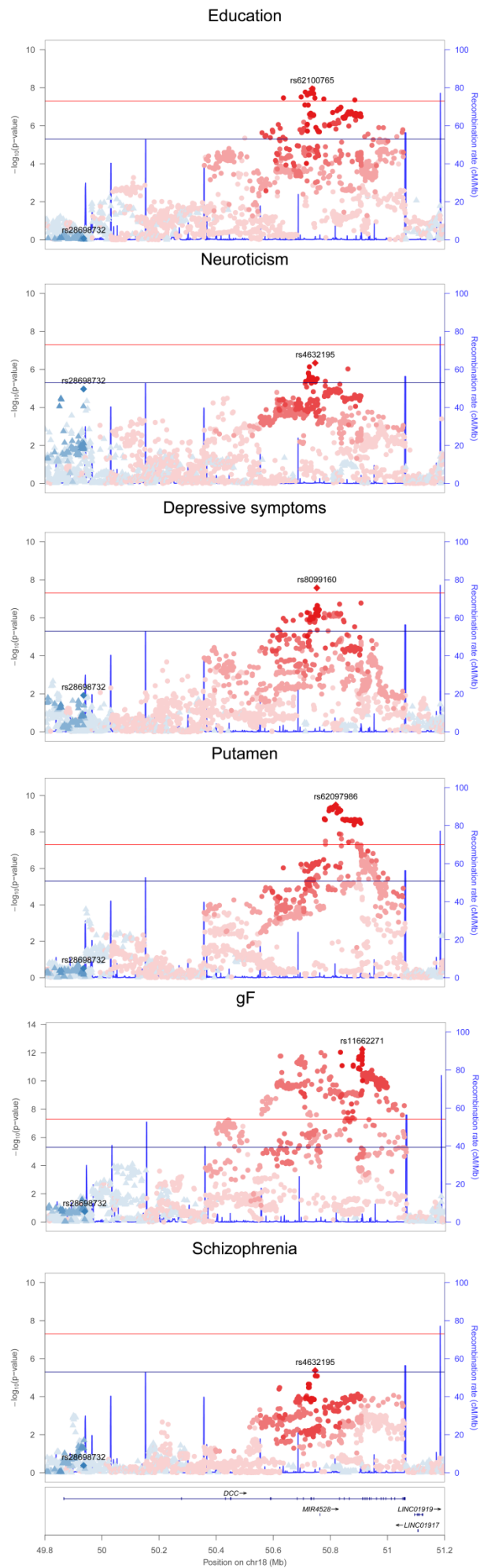

A.

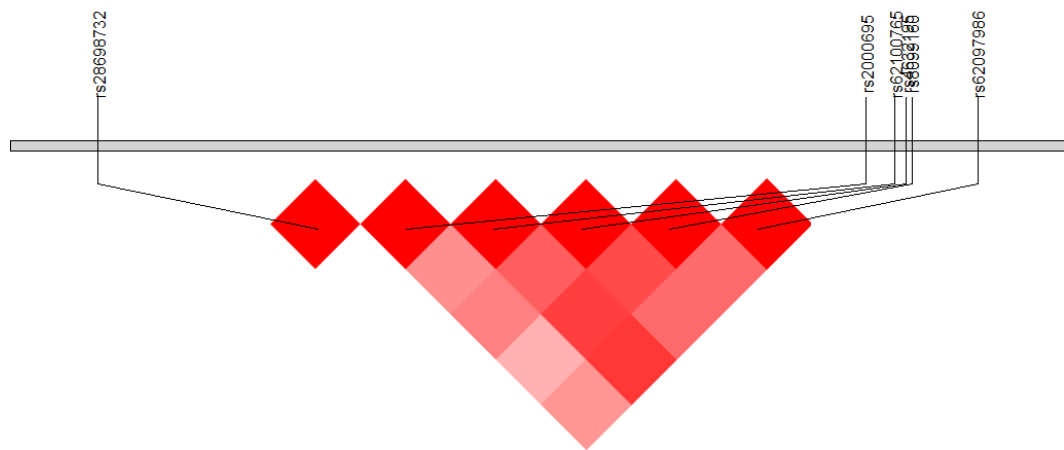

B

Supplement: Supplementary file 5 — Supplementary Figure 4 [file 41398_2021_1530_MOESM5_ESM.pdf]
