# Supplementary material for: Identification of pleiotropy at the gene level between psychiatric disorders and related traits
Source: Transl Psychiatry. 2021 Jul 29;11:410. doi: 10.1038/s41398-021-01530-4 (PMC8322263; doi:10.1038/s41398-021-01530-4)
Supplement: Supplementary file 2 — Supplementary Material [file 41398_2021_1530_MOESM2_ESM.docx]

# Supplementary Material

Identification of pleiotropy at the gene level between psychiatric disorders and related traits

Tatiana Polushina, Niladri Banerjee, Sudheer Giddaluru, Francesco Bettella, Thomas Espeseth, Astri J. Lundervold, Srdjan Djurovic, Sven Cichon, Per Hoffmann, Markus M. Nöthen, NeuroCHARGE Working Group, Vidar M. Steen, Ole A. Andreassen, Stéphanie Le Hellard.

**Supplementary Methods page 2**

**Supplementary Figure Legends page 4**

**List of Supplementary Tables page 5**

**Supplementary Table 7 page 5**

**Supplementary References page 6**

# Supplementary Methods

Schizophrenia (SCZ), Bipolar disorder (BPD), Autism Spectrum Disorder (ASD), Major Depressive Disorder (MDD), Attention deficit hyperactivity disorder (ADHD), Anorexia, Anxiety and Loneliness from the Psychiatric Genomics Consortium (PGC)

Summary statistics from SCZ GWAS^1^, Autism GWAS^2^, MDD GWAS^3^, anorexia nervosa GWAS^4–6^, ADHD GWAS^7^, BPD GWAS^8^ and anxiety GWAS from ANGST (Anxiety Neuro Genetics Study)^9^ were downloaded from <https://www.med.unc.edu/pgc/results-and-downloads>. The data consist of summary statistics for 9,444,231; 9,499,590; 1,235,110; 13,694,841; 5,837,346; 13,414,632; and 6,330,995 variants, respectively.

GWAS results for loneliness were loaded from the same web-site^10^. Here, we used a summary association obtained with linear regression analysis where a continuous phenotype was obtained by summing the scores from all three questions, thus yielding a score between 3 (least lonely) and 9 (most lonely). Summary data for 5,768,558 markers was provided.

Alzheimer’s disease

Summary statistics from the IGAP (International Genomics of Alzheimer’s Project) consortium GWAS^11^ were downloaded from <http://www.pasteur-lille.fr/en/recherche/u744/igap/igap_download.php>. IGAP is a large two-stage study based upon GWASs on individuals of European ancestry. For our analysis, we used the result of stage 1 with meta-analyses of four previously published GWAS datasets consisting of 17,008 Alzheimer’s disease cases and 37,154 controls (The European Alzheimer’s Disease Initiative – EADI; the Alzheimer Disease Genetics Consortium – ADGC; The Cohorts for Heart and Aging Research in Genomic Epidemiology consortium – CHARGE; The Genetic and Environmental Risk in AD consortium - GERAD) for 7,055,881 SNPs.

Subcortical volumes

Summary statistics from the ENIGMA (Enhancing NeuroImaging Genetics through Meta-Analysis) consortium for subcortical brain volumes^12^ were loaded from <http://enigma.ini.usc.edu/download-enigma-gwas-results/>. We analysed the summary meta-statistics after exclusion of PGC SCZ samples. The individual number of markers for each region can be found in Table 1, Supplementary Table 1.

Educational attainment, subjective well-being (SWB), depressive symptoms (DS), neuroticism

Summary statistics for the Educational attainment GWAS^13^ and for SWB, DS and Neuroticism GWAS^14^ were downloaded from http://www.thessgac.org/data. The data consist of summary statistics of 8,146,840; 2,268,674; 6,524,474; and 6,524,432 markers, respectively.

General cognitive function (gF)

Summary statistics of 12,871,897 SNPs associated with general cognitive function^15^ were downloaded from web site <https://www.ccace.ed.ac.uk/node/335>. We used open dataset summary results, where five cohort were removed: The Aging Gene-Environment Susceptibility – Reykjavik Study (AGES), The Atherosclerosis Risk in Communities Study (ARIC), The Cardiovascular Health Study (CHS), The Framingham Heart Study (FHS), and The Genetic Epidemiology Network of Arteriopathy (GENOA), summarizing General Cognitive Function for N = 282,014 from CHARGE consortium.

Extraversion, openness to experience, agreeableness and conscientiousness

Summary statistics for 4 personality traits, extraversion^16^, openness to experience, agreeableness and conscientiousness^17^, were loaded from <http://www.tweelingenregister.org/GPC/>. The numbers of markers in the studies are 6,941,603; 2,305,640; 2,305,461; and 2,305,682, respectively.

Aggressive behavior

The EArly Genetics and Lifecourse Epidemiology (EAGLE) consortium has published GWAS data for children's aggressive behavior^18^ on the web-site <http://www.tweelingenregister.org/EAGLE/>. The summary statistics contain 2,188,528 markers.

Overlapping cohorts

In order to address the question of genetic overlap, we compared signals from individual GWASs. However, here we were faced with the issue of a number of overlapping cohorts between studies and some similar measurements (gF, educational attainment). In our pipeline, we focus on comparisons between three groups of traits: psychiatric disorders, brain volumes and cognitive/personality traits. The summary statistics obtained from brain volumes do not have any overlap with the PGC data for mental disorders and have only a minor overlap with the CHARGE data. Cohort SHIP, which is included in the ENIGMA data, is also present in the Educational attainment, Neuroticism and Anxiety GWASs. Cohort NESDA is present in 7 different GWASs, but the overall overlap between brain volumes and cognitive/personality studies is low.

Within the ENIGMA dataset, all GWASs for different brain substructures were performed on the same individuals. However, when we compared the independent signals selected in cojo-GCTA using the threshold 10^-5^, the overlap within a 50kb window was very poor (**Supplementary Figure 8**).

Within the group of cognitive/personality traits, many cohorts were included in several studies (**Supplementary Figure 9**). Also, the number of years of education is a “proxy” for intelligence and social activity. However, from **Supplementary Figure 9**, overlapping genetic signals were observed for Educational attainment with Neuroticism, gF and Depressive symptoms. For further analysis, we kept all of these studies, but at the interpretation level we focused only on overlaps between groups of mental disorders, brain volumes and cognitive/personality tests and we considered these signals as representing the same signals due to the possibility of overlapping cohorts, similar measurements and some genetic overlaps.

The Current Depression symptoms GWAS is one of the cohorts included in the PGC MDD GWAS. Therefore, at the interpretation stage, we discussed possible overlaps between GWASs of cognitive/personality traits separately.

Repetitive markers

The list of independent signals (associated with a trait) contains 25 cases where exactly the same SNP was selected in two different traits. Six of them were not annotated to any gene. An additional eleven were the only signal annotated to the gene. The remaining 8 SNPs are listed in **Supplementary Tables 4 and 5**.

**Supplementary Figure Legends**

**Supplementary Figure 1.** Pipeline for identification of allelic heterogeneity. See separate image file.

**Supplementary Figure 2.** Regional plots for loci identified by marker-based analysis and corresponding to Scenario I. See separate image file.

**Supplementary Figure 3.** Regional plots for loci identified by marker-based analysis and corresponding to Scenario II. See separate image file.

**Supplementary Figure 4.** Regional plot for the *DCC* gene, one of the genes identified by marker-based analysis and corresponding to Scenario III. See separate image file.

(a) Association plots in the region chr18: 49.8 – 51.2 Mb for SCZ, Depression, Neuroticism, Educational attainment, Intelligence and Putamen studies. (b) Pairwise correlation r^2^ for tagged SNPs.

**Supplementary Figure 5.** Regional plots for loci identified by marker-based analysis and corresponding to mixed case scenarios. See separate image file.

**Supplementary Figure 6.** Regional plots for loci identified by gene-based analysis and corresponding to Scenario I. See separate image file.

**Supplementary Figure 7**. Comparison of the number of associations identified in Scenario I and Scenario III. See separate image file.

**Supplementary Figure 8.** Overlaps between independent signals selected in ENIGMA brain volumes GWASs within 50kb windows. See separate image file.

Each cell contains the number of regions within 50kb windows where signals were selected in two different GWASs using cojo-GCTA at the association level 10^-5^. Diagonal values are the total number of associated signals selected at the association level 10^-5^ in cojo-GCTA.

**Supplementary Figure 9.** Overlaps between cohorts included in cognitive/personality GWASs and independent signals within 50kb windows. See separate image file.

The upper triangle contains the number of regions within 50kb windows where signals were selected in two different traits using cojo-GCTA at the association level 10^-5^. The diagonal values are the numbers of associated signals selected at the association level 10^-5^ in cojo-GCTA.

**List of Supplementary Tables**

**Supplementary Table 1.** Summary of **GWAS SNPs**. Genetic build, source for allele frequency and total number of SNPs for each study and detailed number of SNPs obtained for each study after each QC step. See separate file.

**Supplementary Table 2.** Genes identified by marker-based and gene-based analyses, and corresponding to Scenario I. See separate file.

**Supplementary Table 3.** Genes or genetic blocks identified by marker-based analysis, and corresponding to Scenario II. See separate file.

**Supplementary Table 4.** Genes identified by marker-based and gene-based analyses, and corresponding to Scenario III. See separate file.

**Supplementary Table 5.** Genes identified by marker-based and gene-based analyses, and corresponding to mixed case scenarios. See separate file.

**Supplementary Table 6.** Overview of genes identified by gene-based analysis. See separate file.

**Supplementary Table 7**. Summary statistics for SNPs rs4388249 and rs1368357 from SCZ and Education GWASs.

| SNP | rs4388249 | rs1368357 |
| --- | --- | --- |
| Position | Chr5: 109,036,066 | Chr5:109,189,130 |
| SCZ GWAS |  |  |
| Allele | T/C | T/C |
| OR | 1.075 | 0.974 |
| P-value | 1.025x10^-7^ | 0.020 |
| Frequencies in cases/controls for some separate SCZ substudies |  |  |
| Danish, aarh | 0.1989/0.1712 | 0.3181/0.3541 |
| German, boco | 0.1940/0.1797 | 0.3248/0.3248 |
| Swedish, s234 | 0.2004/0.1828 | 0.3177/0.3308 |
| Norwegian, top8 | 0.1645/0.1849 | 0.3522/0.3364 |
| PEIC, WTCCC2, pewb | 0.1594/0.1493 | 0.3416/0.3244 |
| Education GWAS |  |  |
| Allele | T/C | T/C |
| Effective allele frequency | 0.160 | 0.347 |
| Beta | 0.006 | -0.012 |
| P-value | 0.063 | 3.375x10^-6^ |

**Supplementary References**

1 Ripke S, Neale BM, Corvin A, Walters JTR, Farh K-H, Holmans PA *et al.* Biological insights from 108 schizophrenia-associated genetic loci. *Nature* 2014; **511**: 421–427.

2 Autism Spectrum Disorder Working Group of the Psychiatry Genomics Consortium. Dataset: PGC-ASD summary statistics from a meta-analysis of 5,305 ASD-diagnosed cases and 5,305 pseudocontrols of European descent. 2015http://www.med.unc.edu/pgc/results-and-downloads.

3 Ripke S, Wray NR, Lewis CM, Hamilton SP, Weissman MM, Breen G *et al.* A mega-analysis of genome-wide association studies for major depressive disorder. *Mol Psychiatry* 2013; **18**: 497–511.

4 Boraska V, Franklin CS, Floyd JAB, Thornton LM, Huckins LM, Southam L *et al.* A genome-wide association study of anorexia nervosa. *Mol Psychiatry* 2014; **19**: 1085–1094.

5 Anorexia Working Group of the Psychiatric Genomics Consortium (PGC-AN). Anorexia Nervosa. 2016.

6 Wang K, Zhang H, Bloss CS, Duvvuri V, Kaye W, Schork NJ *et al.* A genome-wide association study on common SNPs and rare CNVs in anorexia nervosa. *Mol Psychiatry* 2011; **16**: 949–959.

7 Martin J, Walters RK, Demontis D, Mattheisen M, Lee SH, Robinson E *et al.* A Genetic Investigation of Sex Bias in the Prevalence of Attention-Deficit/Hyperactivity Disorder. *Biol Psychiatry* 2018; **83**: 1044–1053.

8 Stahl E, Breen G, Forstner AJ, McQuillin A, Ripke S, Trubetskoy V *et al.* Genome-wide association study identifies 30 loci associated with bipolar disorder. *Nat Genet* 2019; **51**: 793–803.

9 Otowa T, Hek K, Lee M, Byrne EM, Mirza SS, Nivard MG *et al.* Meta-analysis of genome-wide association studies of anxiety disorders. *Mol Psychiatry* 2016; **21**: 1391–1399.

10 Gao J, Davis LK, Hart AB, Sanchez-Roige S, Han L, Cacioppo JT *et al.* Genome-Wide Association Study of Loneliness Demonstrates a Role for Common Variation. Neuropsychopharmacology. 2017; **42**: 811–821.

11 Lambert J-C, Ibrahim-Verbaas CA, Harold D, Naj AC, Sims R, Bellenguez C *et al.* Meta-analysis of 74,046 individuals identifies 11 new susceptibility loci for Alzheimer’s disease. *Nat Genet* 2013; **45**: 1452–1458.

12 Hibar DP, Stein JL, Renteria ME, Arias-Vasquez A, Desrivieres S, Jahanshad N *et al.* Common genetic variants influence human subcortical brain structures. *Nature* 2015; **520**: 224–229.

13 Okbay A, Beauchamp JP, Fontana MA, Lee JJ, Pers TH, Rietveld CA *et al.* Genome-wide association study identifies 74 loci associated with educational attainment. *Nature* 2016; **533**: 539–542.

14 Okbay A, Baselmans BML, De Neve J-E, Turley P, Nivard MG, Fontana MA *et al.* Genetic variants associated with subjective well-being, depressive symptoms, and neuroticism identified through genome-wide analyses. *Nat Genet* 2016; **48**: 624–633.

15 Davies G, Lam M, Harris SE, Trampush JW, Luciano M, Hill WD *et al.* Study of 300,486 individuals identifies 148 independent genetic loci influencing general cognitive function. *Nat Commun* 2018; **9**: 2098.

16 Van den Berg SM, de Moor MHM, McGue M, Pettersson E, Terracciano A, Verweij KJH *et al.* Harmonization of Neuroticism and Extraversion phenotypes across inventories and cohorts in the Genetics of Personality Consortium: an application of Item Response Theory. *Behav Genet* 2014; **44**: 295–313.

17 De Moor MHM, Costa PT, Terracciano A, Krueger RF, de Geus EJC, Toshiko T *et al.* Meta-analysis of genome-wide association studies for personality. *Mol Psychiatry* 2012; **17**: 337–349.

18 Pappa I, Pourcain BS, Benke K, Cavadino A, Hakulinen C, Nivard MG *et al.* Neuropsychiatric Genetics A Genome-Wide Approach to Children ’ s Aggressive Behavior : The EAGLE consortium. *Am J Med Genet B Neuropsychiatr Genet* 2016; **171**: 562–572.
