# Supplementary material for: Identification of pleiotropy at the gene level between psychiatric disorders and related traits
Source: Transl Psychiatry. 2021 Jul 29;11:410. doi: 10.1038/s41398-021-01530-4 (PMC8322263; doi:10.1038/s41398-021-01530-4)
Supplement: Supplementary file 3 — Supplementary Figure 2 [file 41398_2021_1530_MOESM3_ESM.pdf]

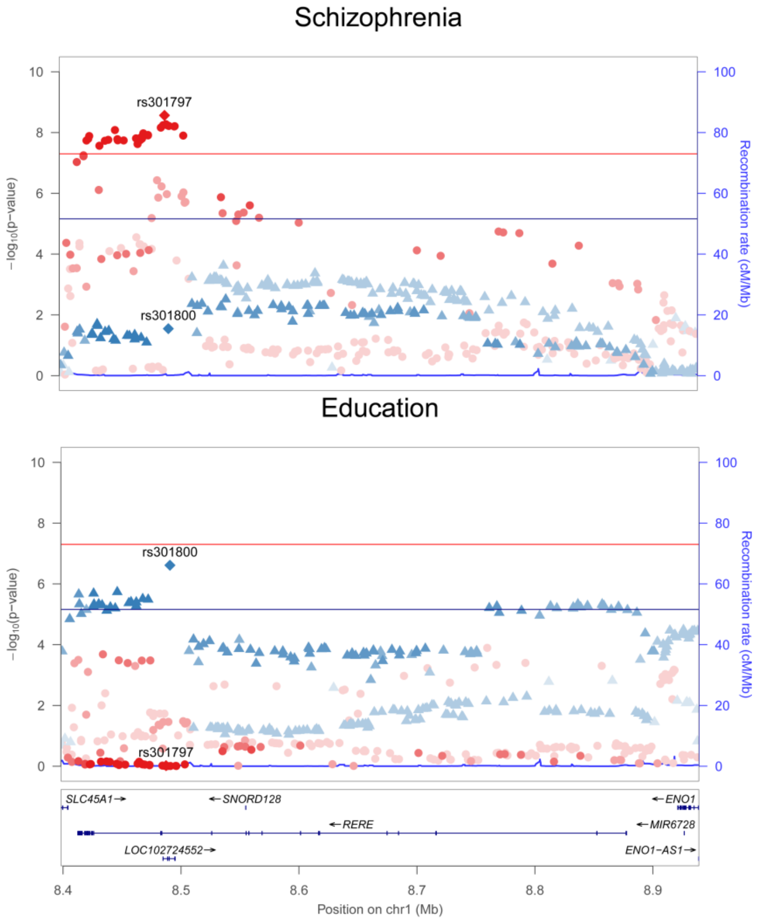
Schizophrenia
rs301797
rs301800
−log10(p−value)
Recombination rate (cM/Mb)
Education
rs301800
rs301797
SLC45A1→
←SNORD128
←ENO1
←RERE
←MIR6728
LOC102724552→
ENO1−AS1→
8.4
8.5
8.6
8.7
8.8
8.9
Position on chr1 (Mb)

A.

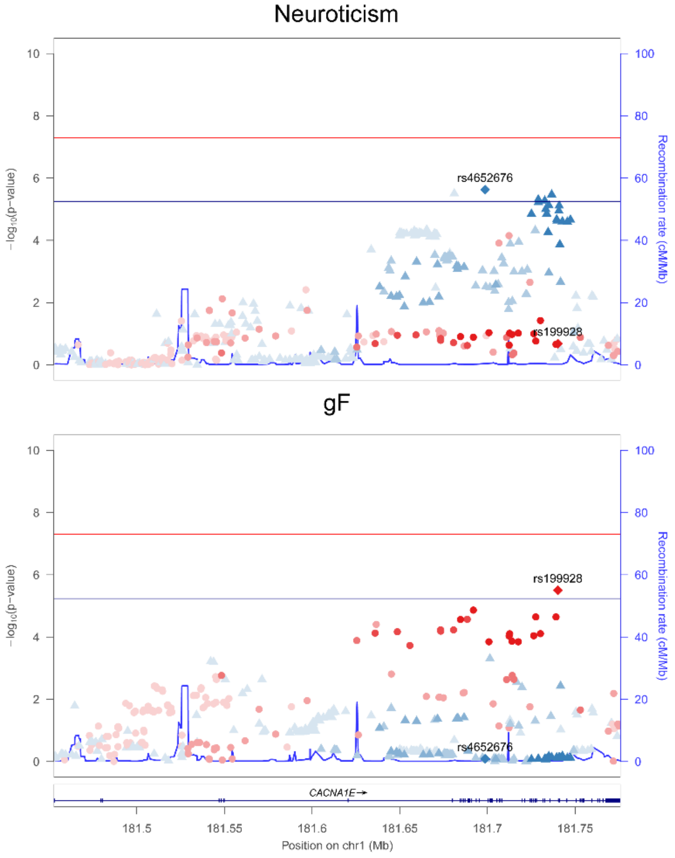
Neuroticism
rs4652676
rs199928
−log10(p−value)
Recombination rate (cM/Mb)
gF
rs199928
rs4652676
CACNA1E→
181.5
181.55
181.6
181.65
181.7
181.75
Position on chr1 (Mb)

B.

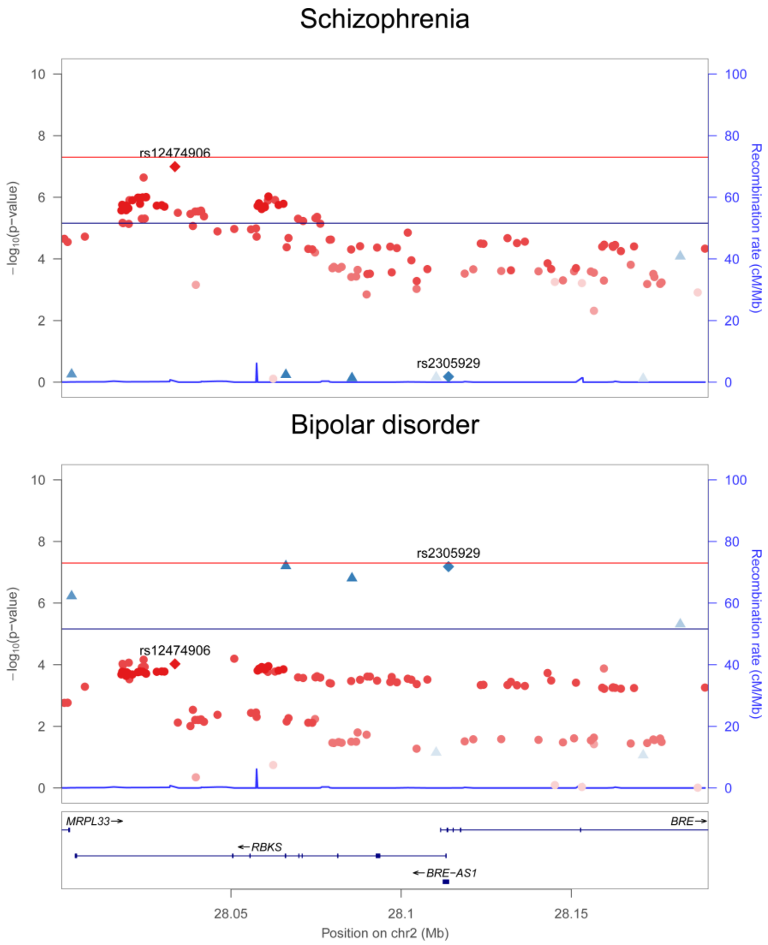
Schizophrenia
rs12474906
rs2305929
−log10(p−value)
Recombination rate (cM/Mb)
Bipolar disorder
rs2305929
rs12474906
−log10(p−value)
Recombination rate (cM/Mb)
MRPL33→
BRE→
←RBKS
←BRE−AS1
28.05
28.1
28.15
Position on chr2 (Mb)

C.

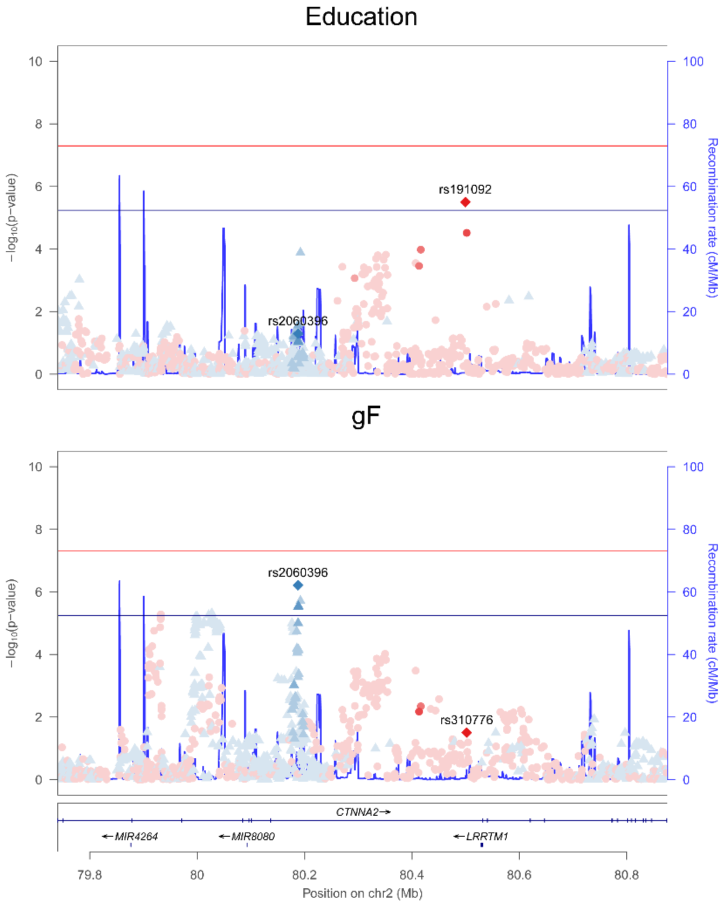
Education
rs191092
rs2060396
−log10(p−value)
Recombination rate (cM/Mb)
gF
rs2060396
rs310776
−log10(p−value)
Recombination rate (cM/Mb)
CTNNA2→
←MIR4264
←MIR8080
←LRRTM1
79.8
80
80.2
80.4
80.6
80.8
Position on chr2 (Mb)

D.

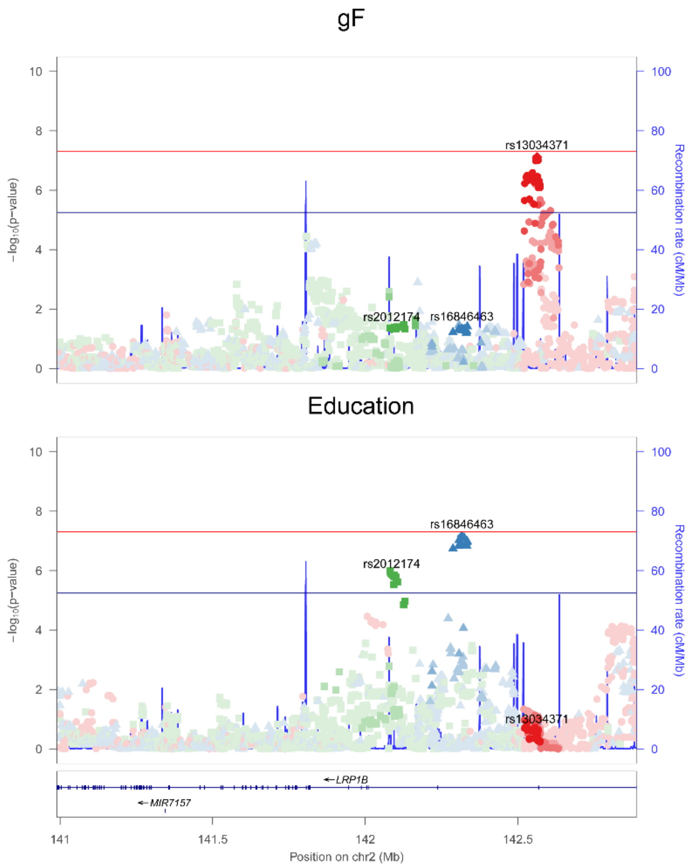
gF
rs13034371
rs2012174
rs16846463
Education
rs16846463
rs2012174
rs13034371
−log10(p−value)
Recombination rate (cM/Mb)
LRP1B
MIR7157
141
141.5
142
142.5
Position on chr2 (Mb)

E.

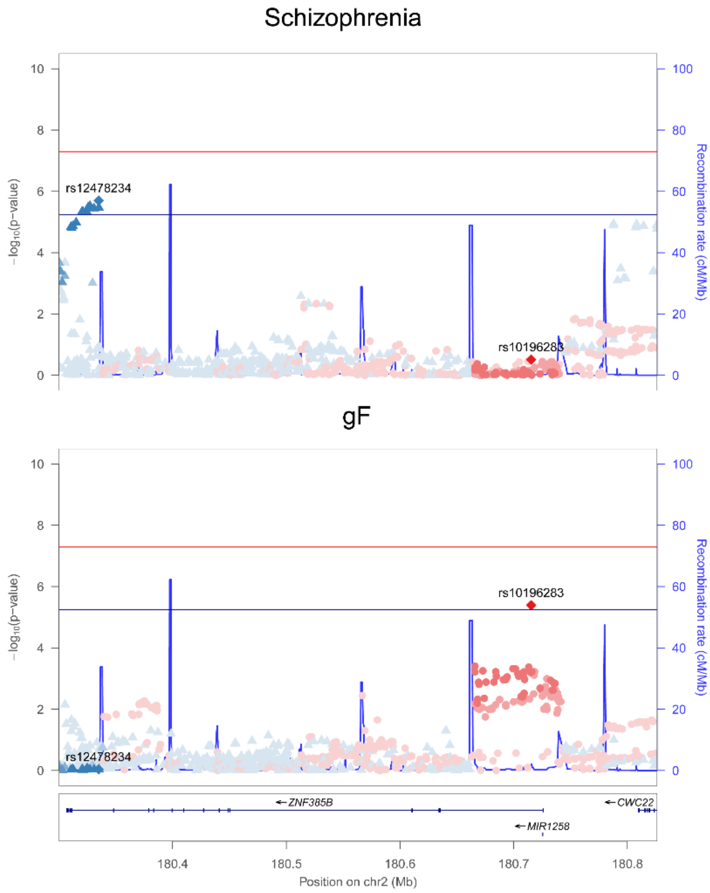
Schizophrenia
rs12478234
rs10196283
gF
rs10196283
rs12478234
−log10(p−value)
Recombination rate (cM/Mb)
ZNF385B
CWC22
MIR1258
180.4
180.5
180.6
180.7
180.8
Position on chr2 (Mb)

F.

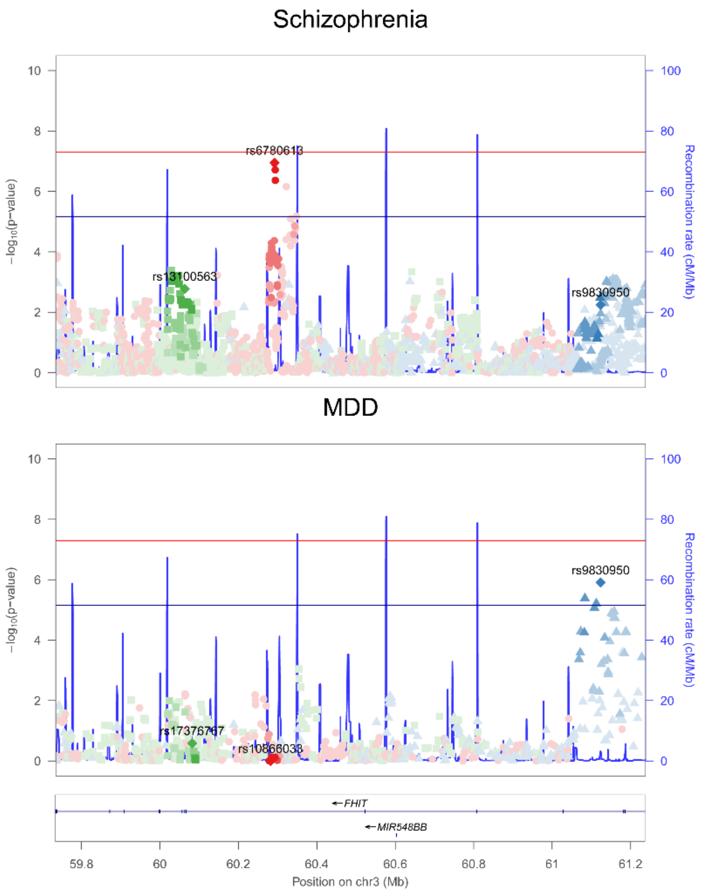
Schizophrenia
MDD
rs6780613
rs13100563
rs9830950
rs17376767
rs10866033
−log10(p−value)
Recombination rate (cM/Mb)
FHIT
MIR548BB
Position on chr3 (Mb)

G.

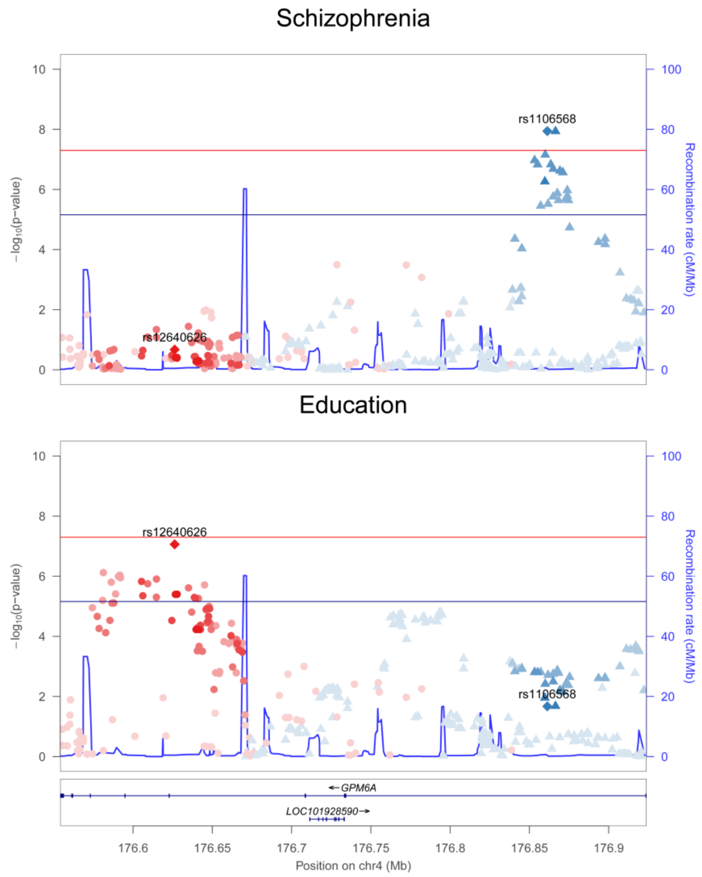
Schizophrenia
Education
rs1106568
rs12640626
−log10(p−value)
Recombination rate (cM/Mb)
GPM6A
LOC101928590
Position on chr4 (Mb)

H.

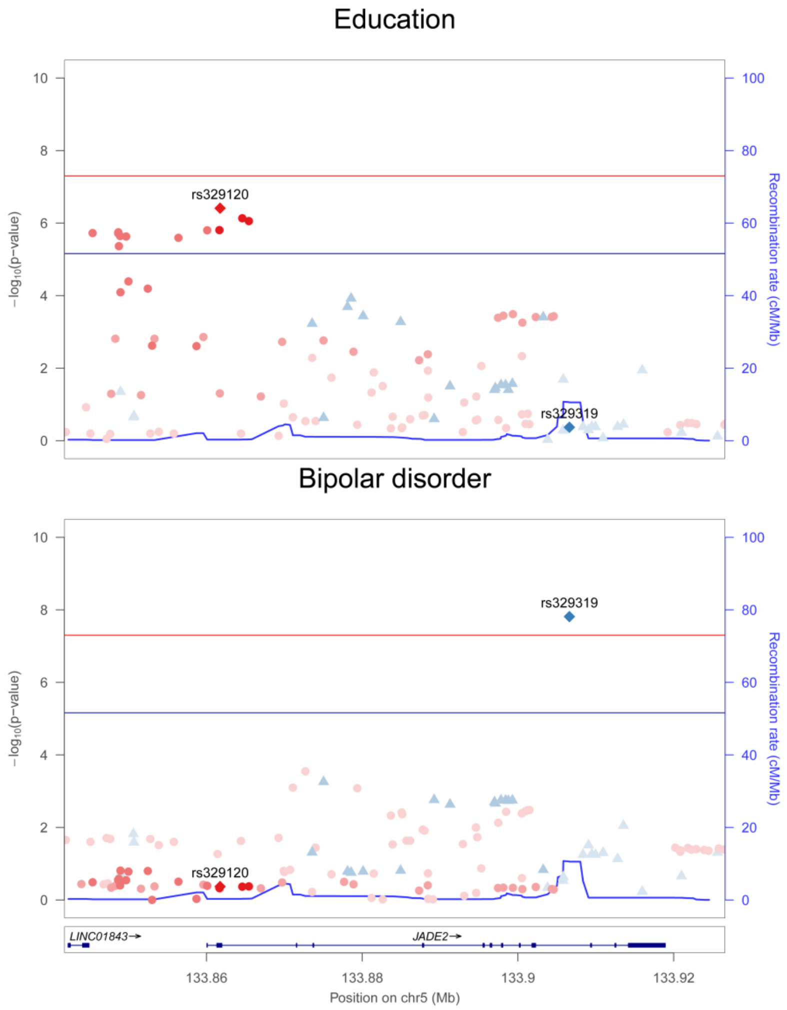
Education
rs329120
rs329319
−log10(p−value)
Recombination rate (cM/Mb)
Bipolar disorder
rs329319
rs329120
LINC01843→
JADE2→
133.86
133.88
133.9
133.92
Position on chr5 (Mb)

I.

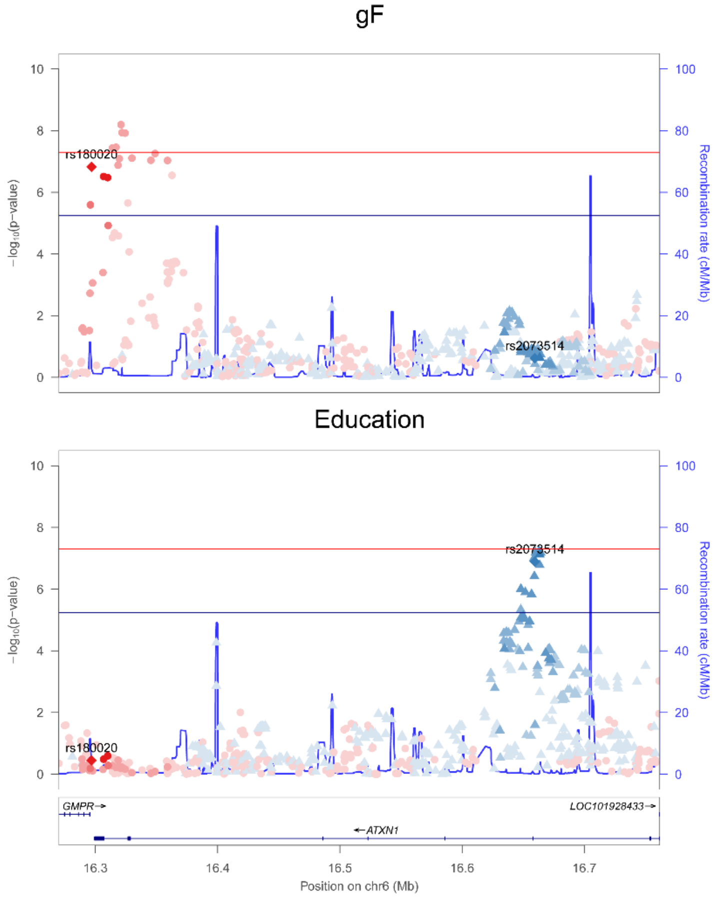
gF
rs180020
rs2073514
−log10(p−value)
Recombination rate (cM/Mb)
Education
rs2073514
rs180020
GMPR→
LOC101928433→
←ATXN1
16.3
16.4
16.5
16.6
16.7
Position on chr6 (Mb)

J.

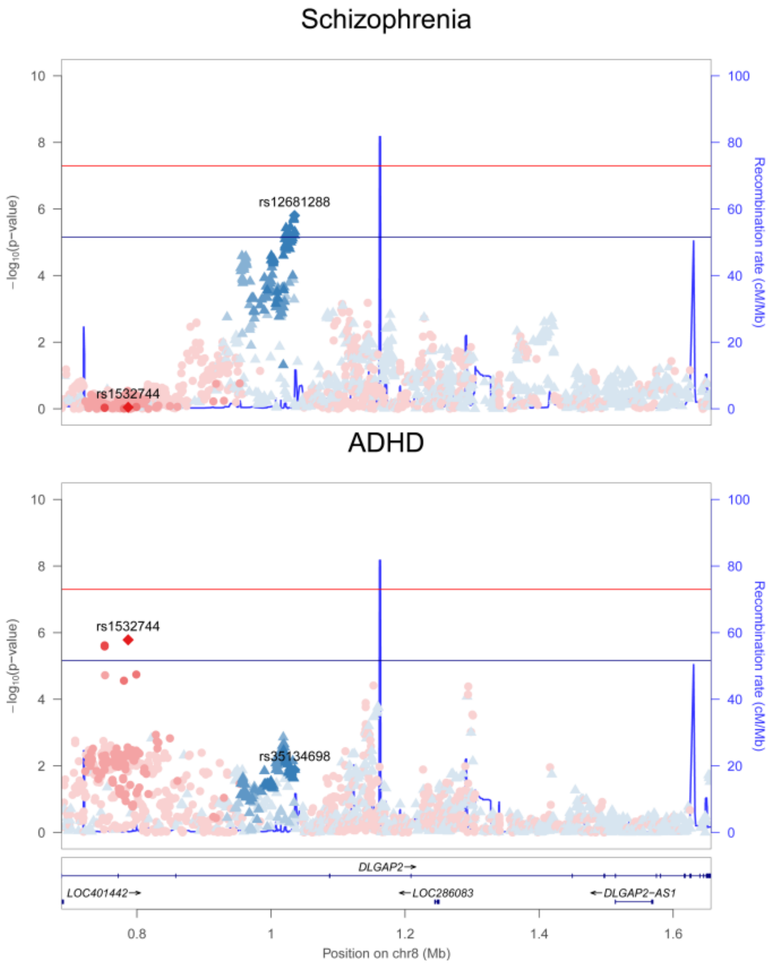

Schizophrenia
ADHD
rs12681288
rs1532744
rs35134698
−log10(p−value)
Recombination rate (cM/Mb)
DLGAP2
LOC401442
LOC286083
DLGAP2−AS1
0.8
1
1.2
1.4
1.6
Position on chr8 (Mb)

K.

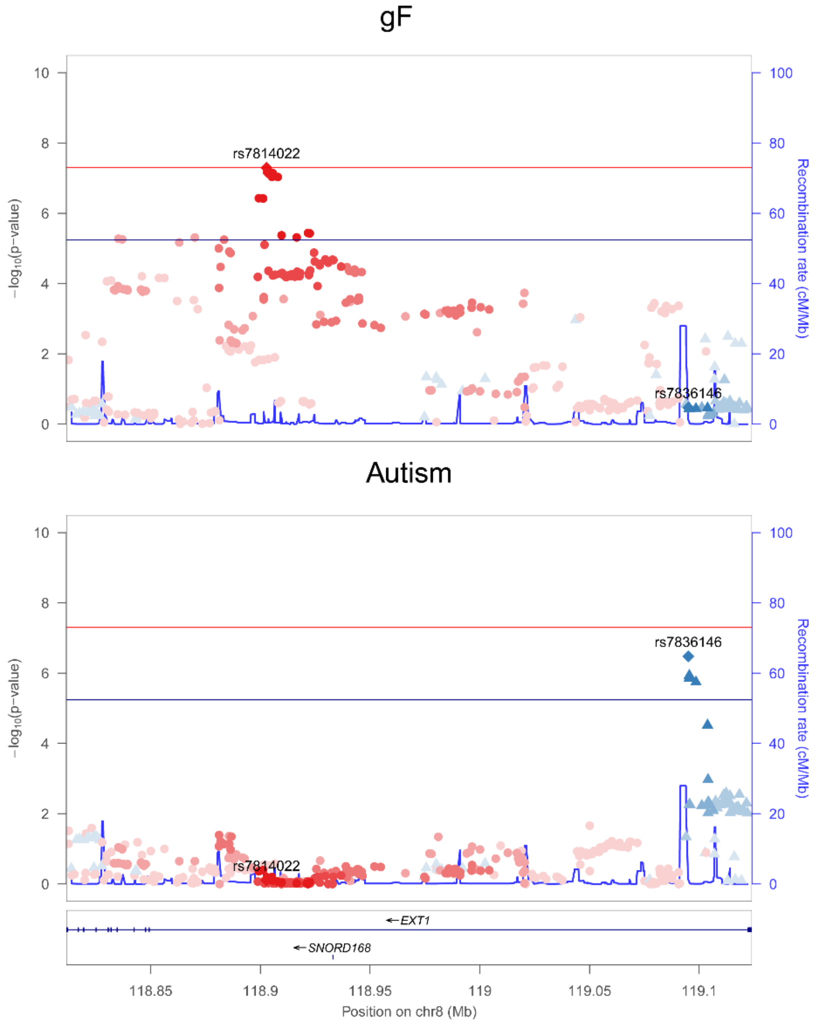

gF
Autism
rs7814022
rs7836146
−log10(p−value)
Recombination rate (cM/Mb)
EXT1
SNORD168
118.85
118.9
118.95
119
119.05
119.1
Position on chr8 (Mb)

L.

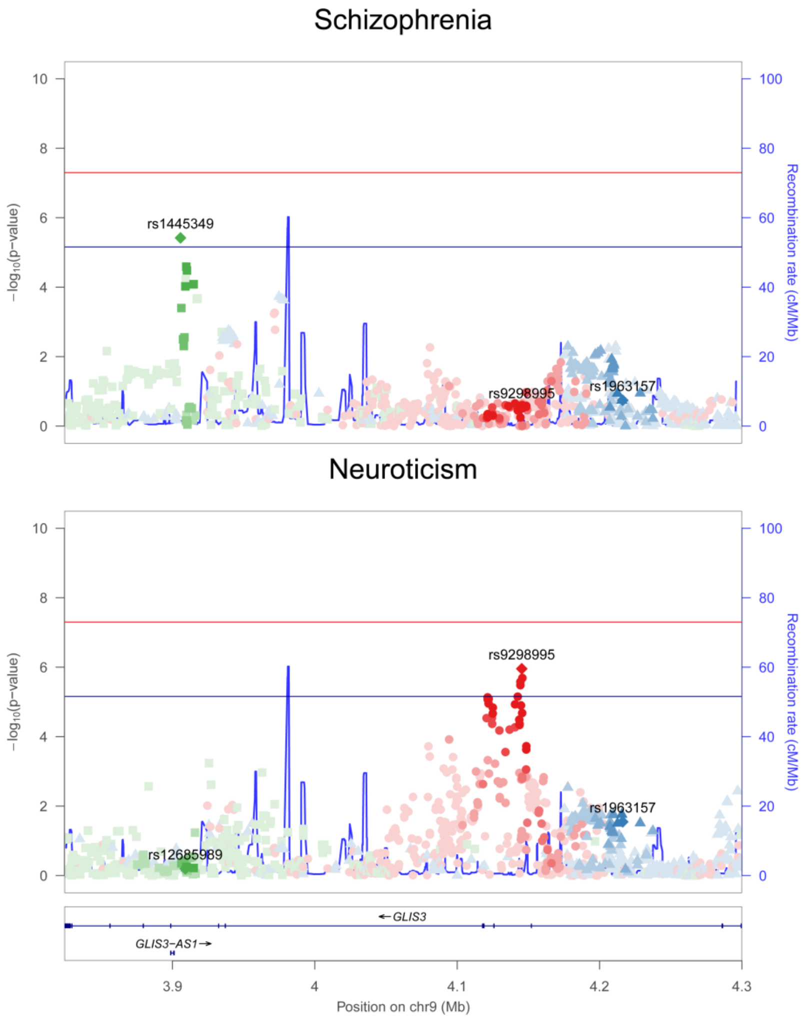
Schizophrenia
rs1445349
rs9298995
rs1963157
Neuroticism
rs9298995
rs1963157
rs12685989
−log10(p−value)
Recombination rate (cM/Mb)
←GLIS3
GLIS3−AS1→
3.9
4
4.1
4.2
4.3
Position on chr9 (Mb)

M.

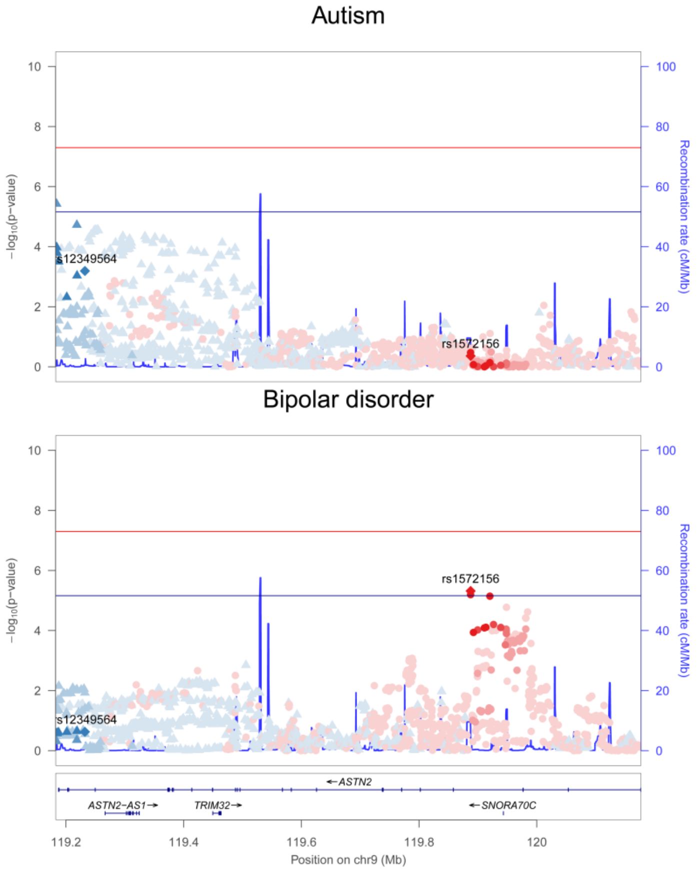
Autism
rs12349564
rs1572156
Bipolar disorder
rs1572156
rs12349564
−log10(p−value)
Recombination rate (cM/Mb)
←ASTN2
ASTN2−AS1→
TRIM32→
←SNORA70C
119.2
119.4
119.6
119.8
120
Position on chr9 (Mb)

N.

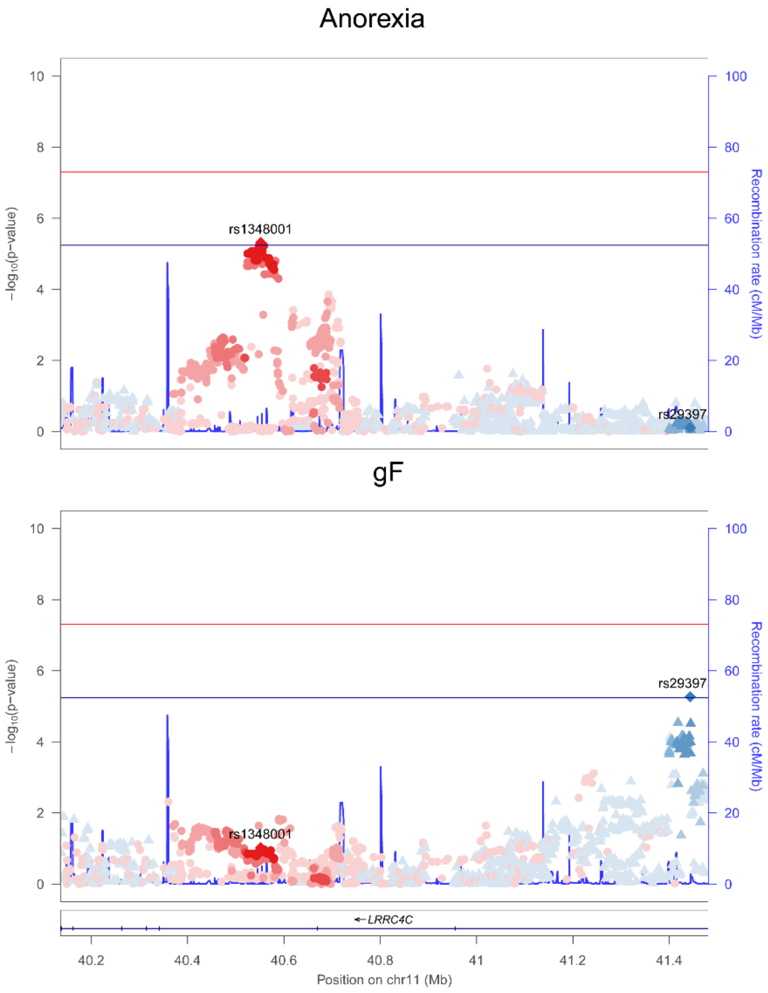
Anorexia
rs1348001
rs29397
gF
rs29397
rs1348001
LRRC4C
−log10(p-value)
Recombination rate (cM/Mb)
Position on chr11 (Mb)

O.

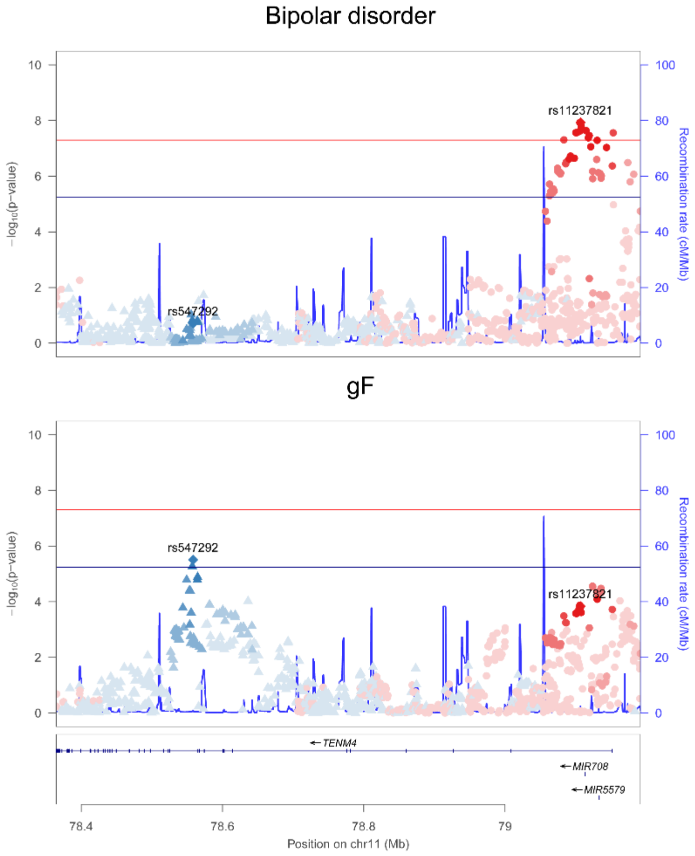
Bipolar disorder
rs11237821
rs547292
gF
rs547292
rs11237821
TENM4
MIR708
MIR5579
−log10(p-value)
Recombination rate (cM/Mb)
Position on chr11 (Mb)

P.

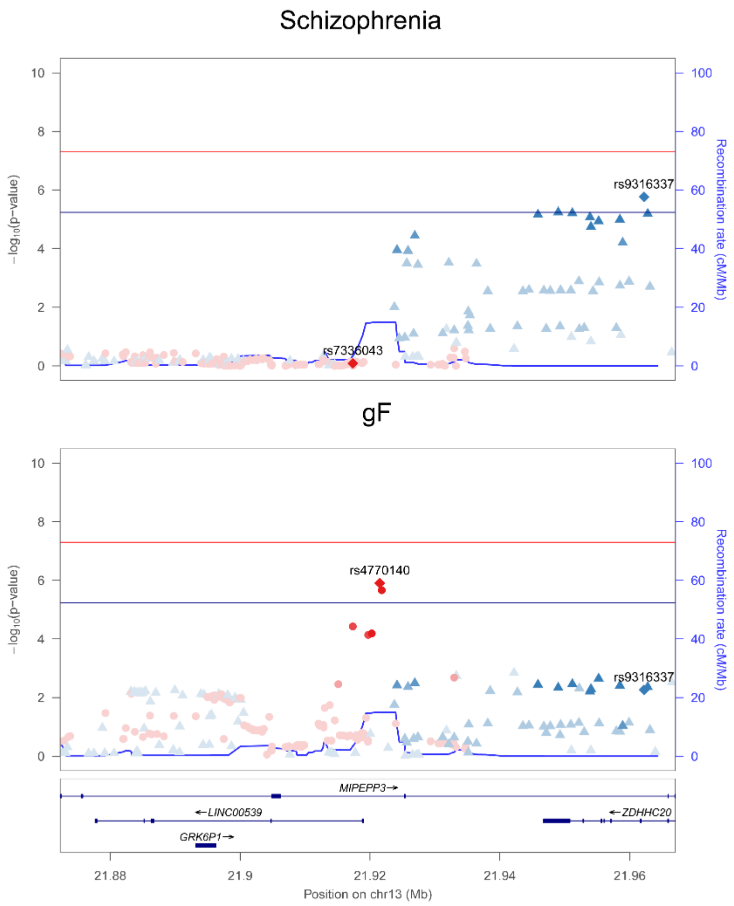
Schizophrenia
−log10(p−value)
Recombination rate (cM/Mb)
rs9316337
rs7336043
gF
rs4770140
rs9316337
MIPEPP3→
←LINC00539
←ZDHHC20
GRK6P1→
21.88
21.9
21.92
21.94
21.96
Position on chr13 (Mb)

Q.

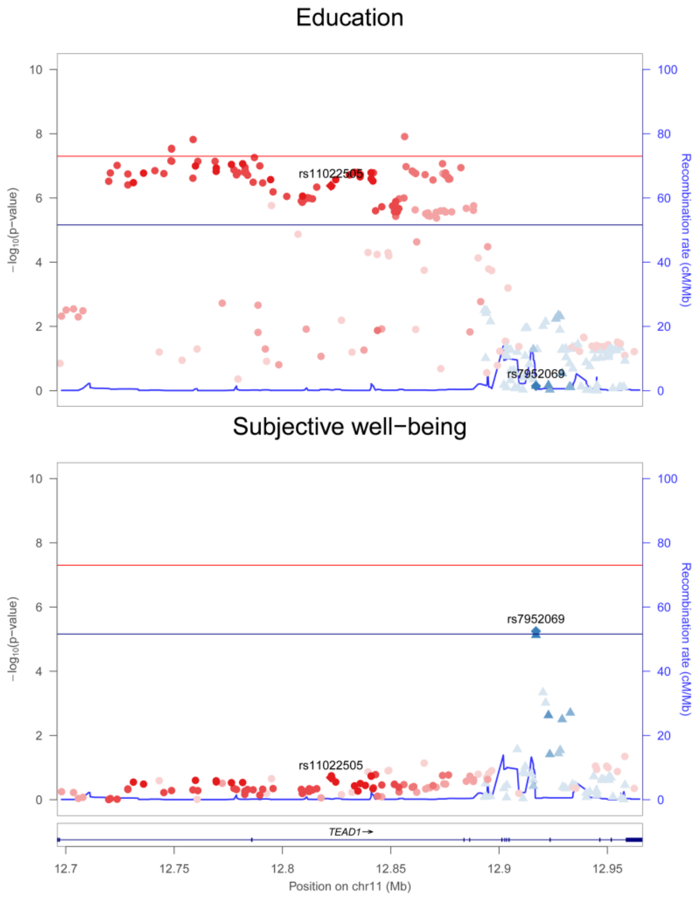
Education
−log10(p−value)
Recombination rate (cM/Mb)
rs11022505
rs7952069
Subjective well−being
rs7952069
rs11022505
TEAD1→
12.7
12.75
12.8
12.85
12.9
12.95
Position on chr11 (Mb)

R.

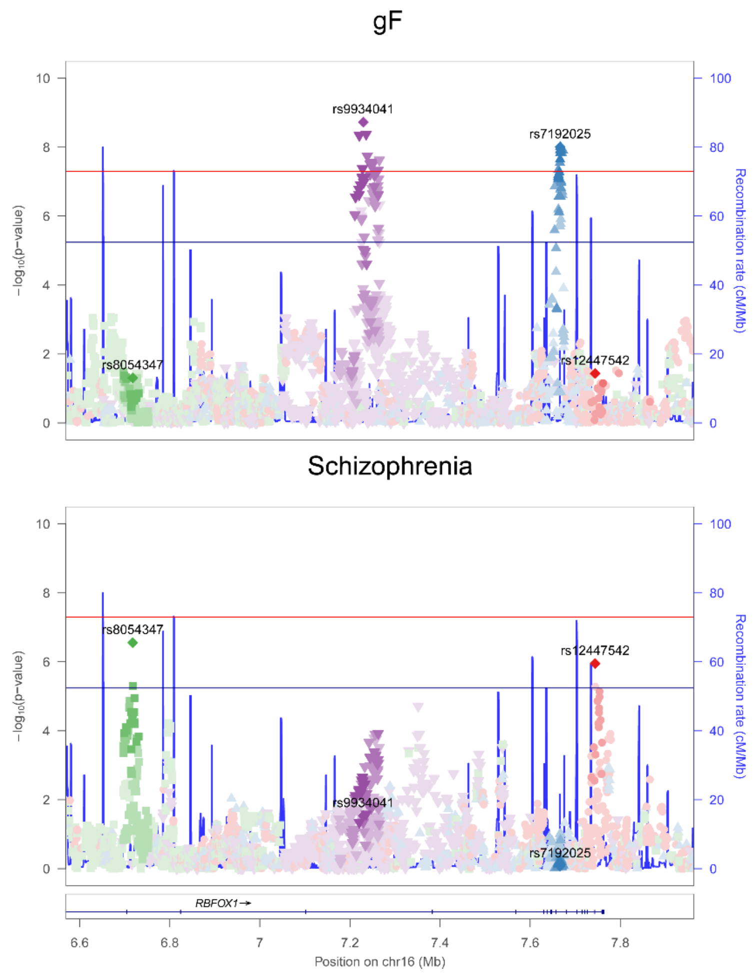
gF
rs9934041
rs7192025
rs8054347
rs12447542
Schizophrenia
rs8054347
rs12447542
rs9934041
rs7192025
RBFOX1
−log10(p−value)
Recombination rate (cM/Mb)
Position on chr16 (Mb)

S.

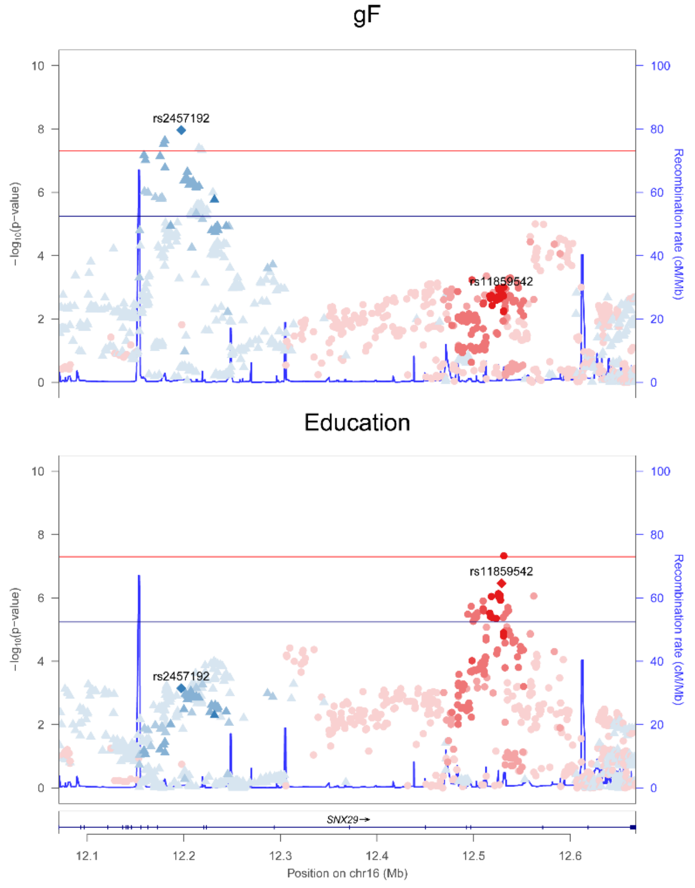
gF
rs2457192
rs11859542
Education
rs11859542
rs2457192
SNX29
−log10(p−value)
Recombination rate (cM/Mb)
Position on chr16 (Mb)

T.

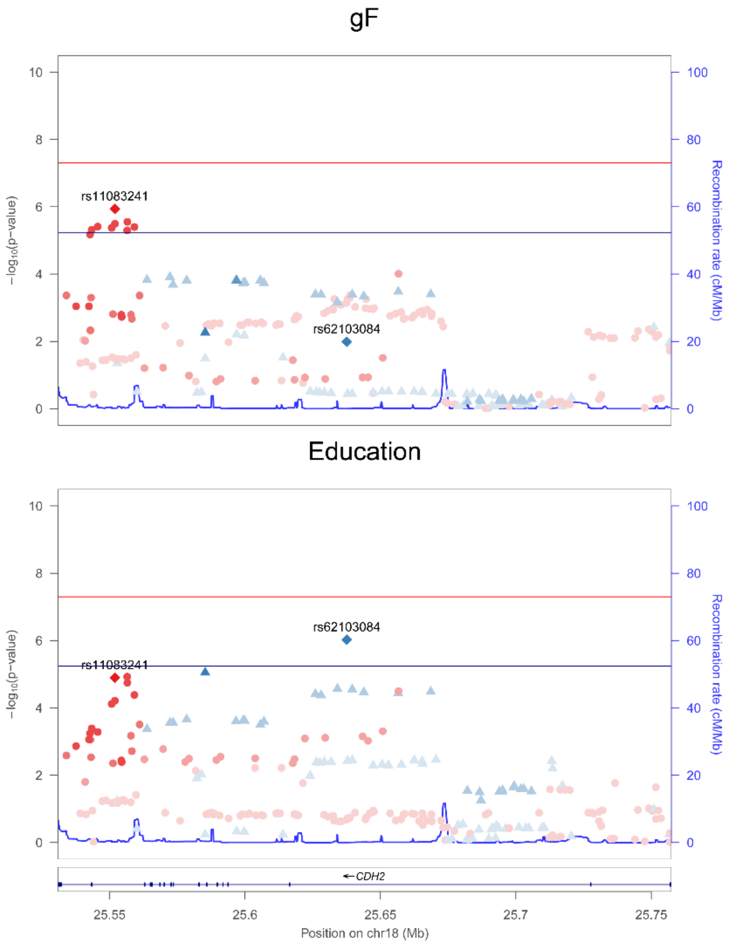
gF
Education
rs11083241
rs62103084
−log10(p−value)
Recombination rate (cM/Mb)
←CDH2
25.55
25.6
25.65
25.7
25.75
Position on chr18 (Mb)

U.

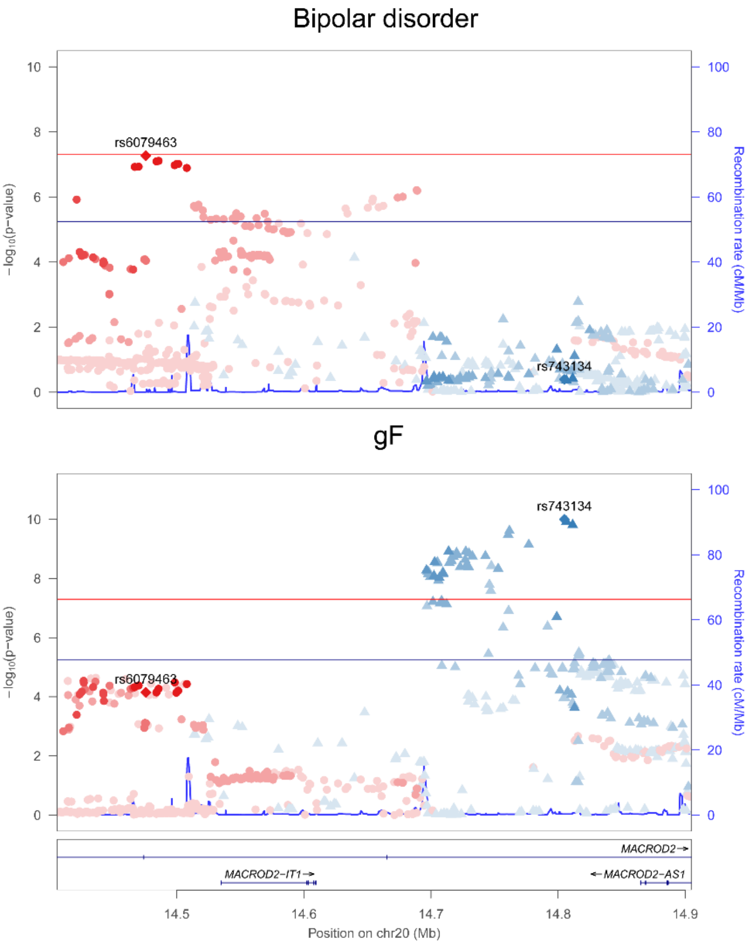
Bipolar disorder
gF
rs6079463
rs743134
−log10(p−value)
Recombination rate (cM/Mb)
MACROD2→
MACROD2−IT1→
←MACROD2−AS1
14.5
14.6
14.7
14.8
14.9
Position on chr20 (Mb)

V.

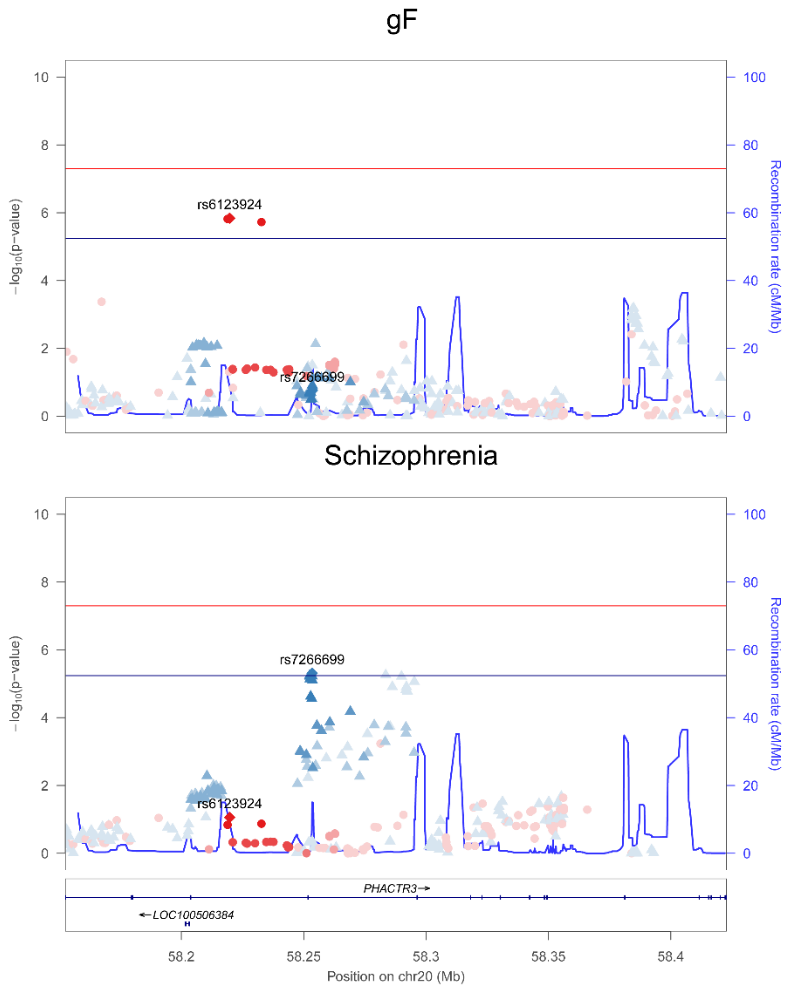
gF
Schizophrenia
rs6123924
rs7266699
−log10(p−value)
Recombination rate (cM/Mb)
0
2
4
6
8
10
20
40
60
80
100
PHACTR3→
←LOC100506384
58.2
58.25
58.3
58.35
58.4
Position on chr20 (Mb)

W.

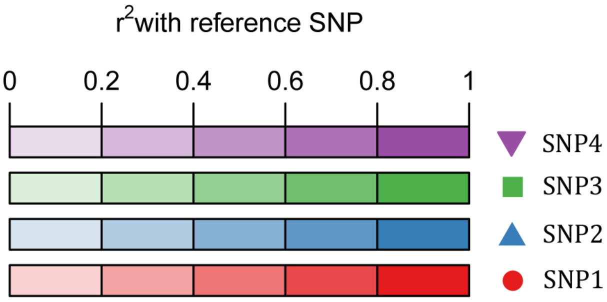
r2with reference SNP
0
0.2
0.4
0.6
0.8
1
SNP4
SNP3
SNP2
SNP1

X.
