# Supplementary material for: Identification of pleiotropy at the gene level between psychiatric disorders and related traits
Source: Transl Psychiatry. 2021 Jul 29;11:410. doi: 10.1038/s41398-021-01530-4 (PMC8322263; doi:10.1038/s41398-021-01530-4)
Supplement: Supplementary file 6 — Supplementary Figure 5 [file 41398_2021_1530_MOESM6_ESM.pdf]

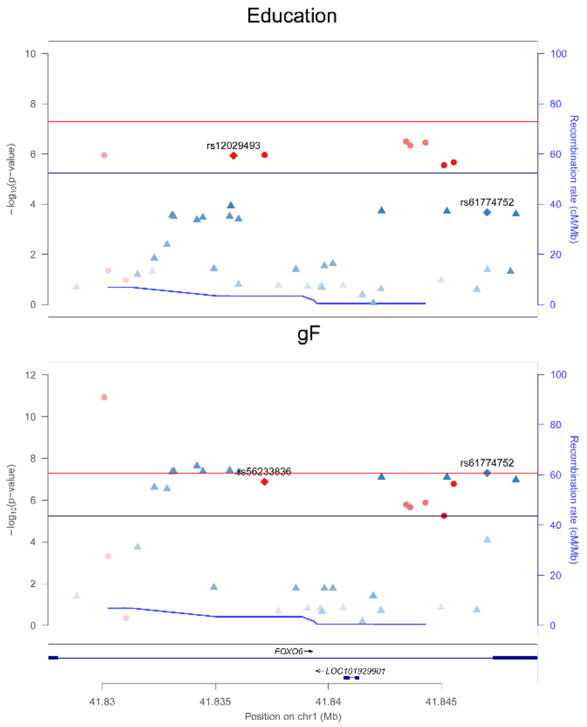
Education
gF
rs12029493
rs61774752
rs56233836
−log10(p−value)
Recombination rate (cM/Mb)
FOXO6
LOC101929901
41.83
41.835
41.84
41.845
Position on chr1 (Mb)

A.

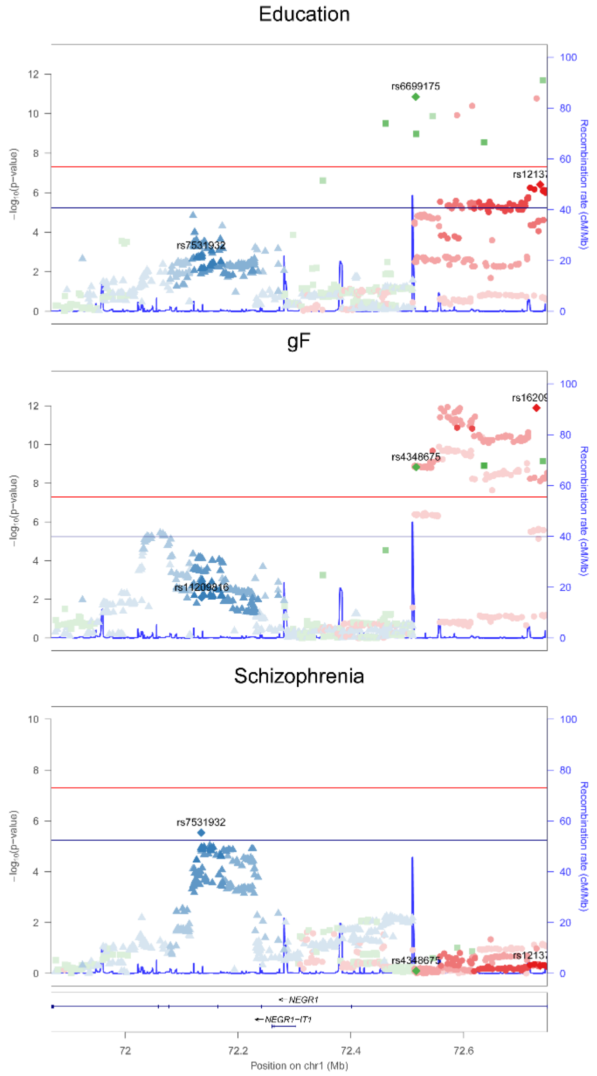
Education
gF
Schizophrenia
rs6699175
rs7531932
rs4348675
rs11209916
−log10(p−value)
Recombination rate (cM/Mb)
NEGR1
NEGR1−IT1
72
72.2
72.4
72.6
Position on chr1 (Mb)

B.

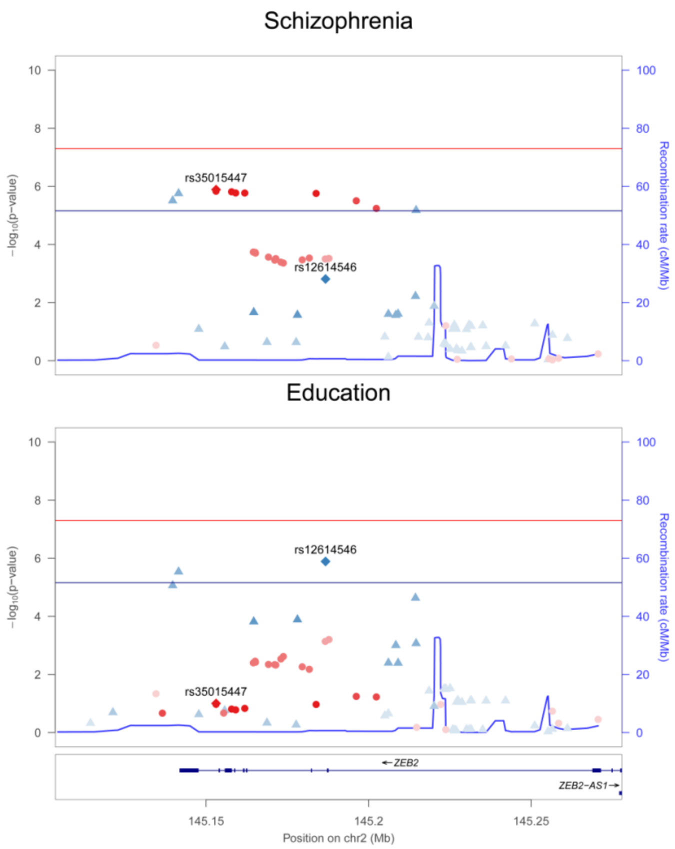
Schizophrenia
Education
rs35015447
rs12614546
−log10(p−value)
Recombination rate (cM/Mb)
ZEB2
ZEB2−AS1
145.15
145.2
145.25
Position on chr2 (Mb)

C.

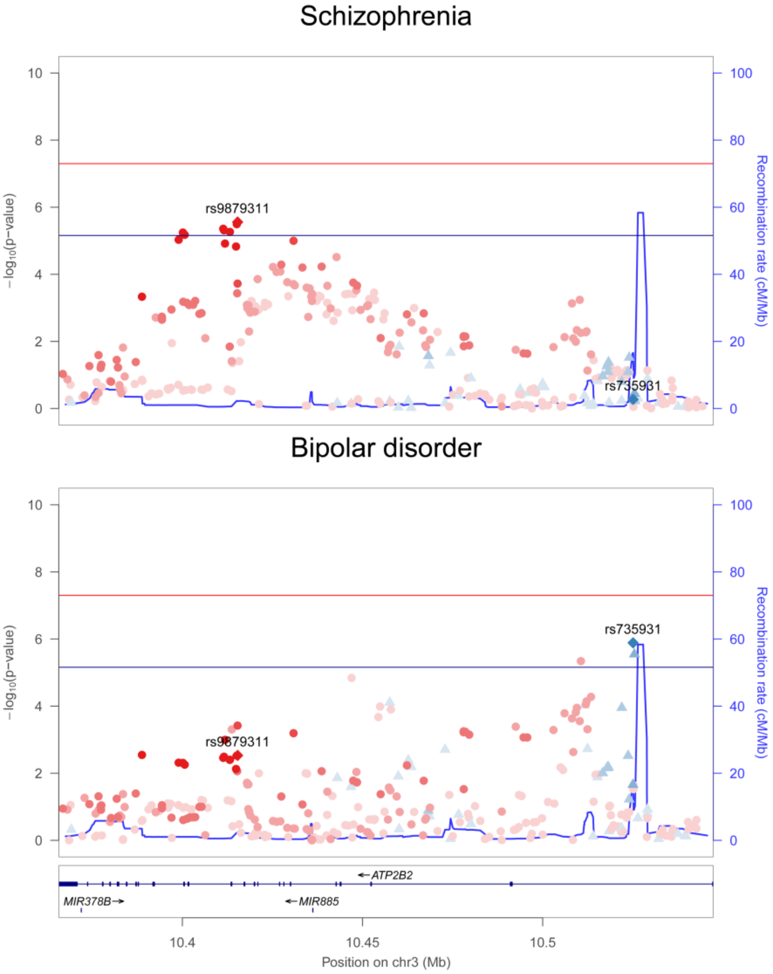
Schizophrenia
Bipolar disorder
rs9879311
rs735931
−log10(p−value)
Recombination rate (cM/Mb)
ATP2B2
MIR378B
MIR885
10.4
10.45
10.5
Position on chr3 (Mb)

D.

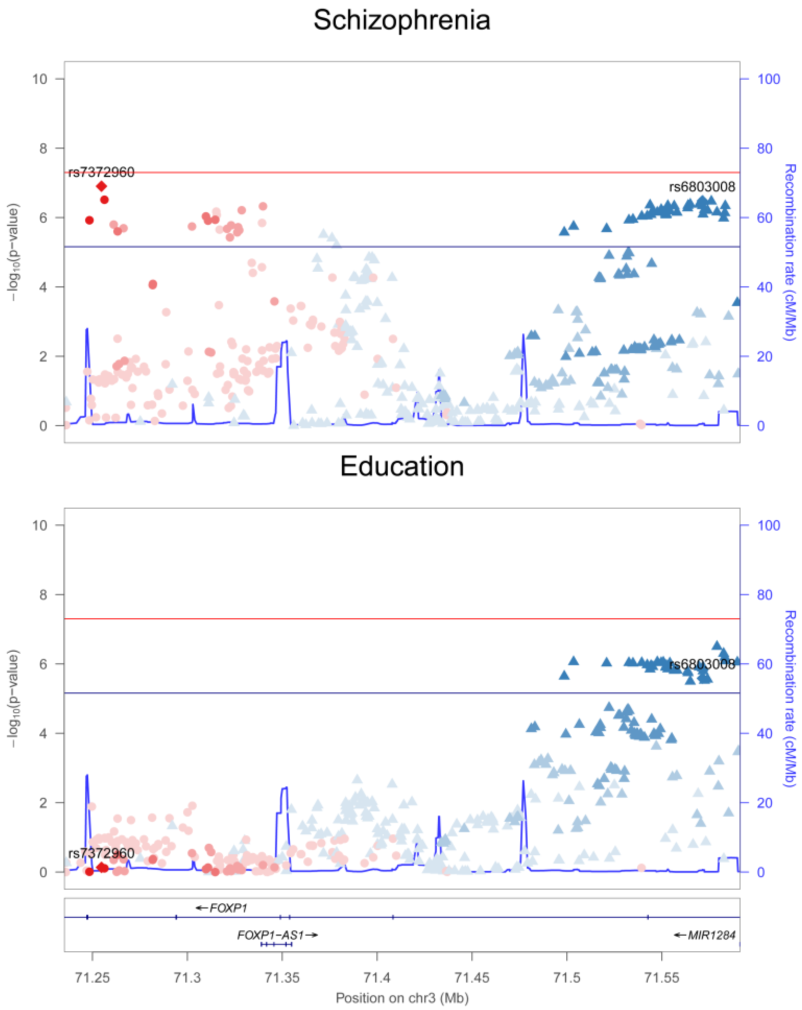
Schizophrenia
Education
rs7372960
rs6803008
−log10(p-value)
Recombination rate (cM/Mb)
FOXP1
FOXP1−AS1
MIR1284
71.25
71.3
71.35
71.4
71.45
71.5
71.55
Position on chr3 (Mb)

E.

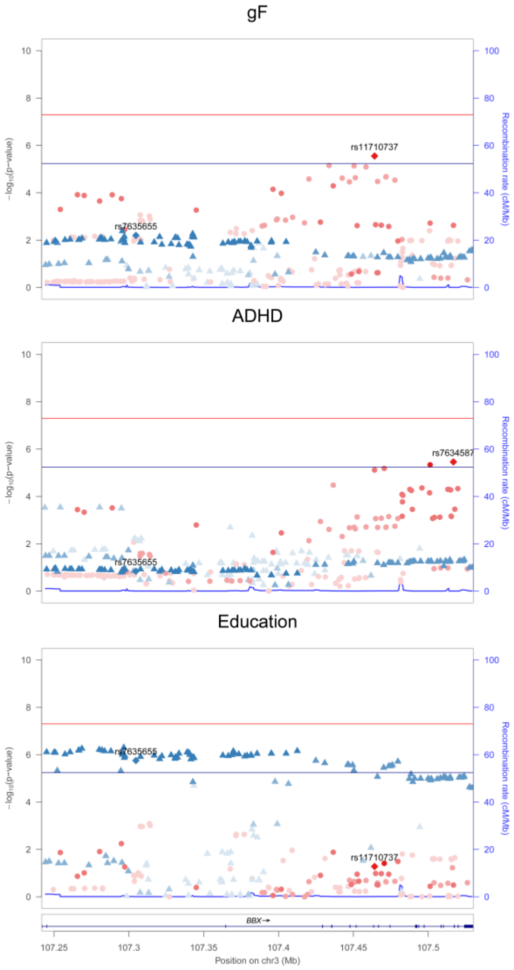
gF
ADHD
Education
rs11710737
rs7635655
rs7634587
−log10(p-value)
Recombination rate (cM/Mb)
BBX
107.25
107.3
107.35
107.4
107.45
107.5
Position on chr3 (Mb)

F.

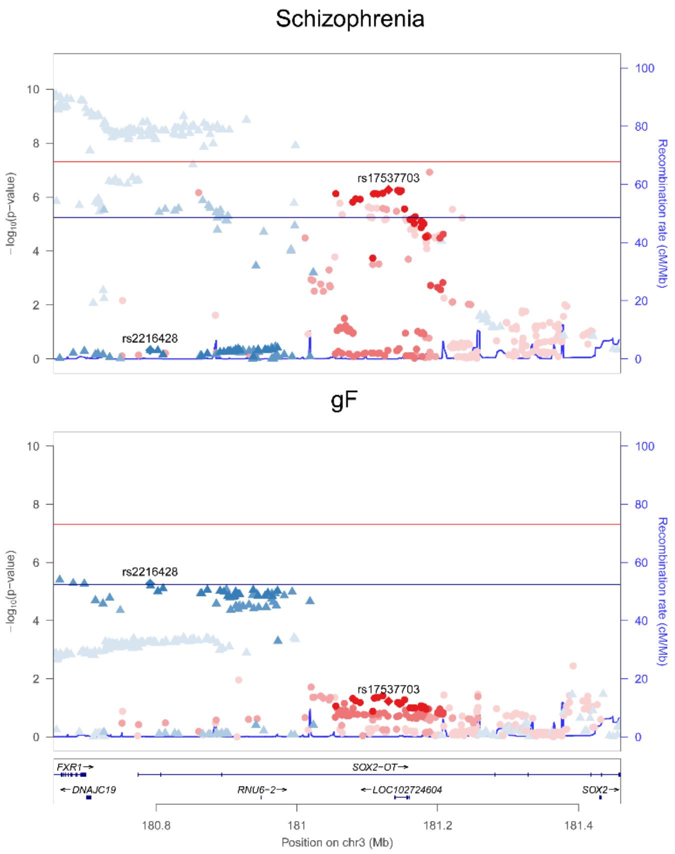
Schizophrenia
gF
rs17537703
rs2216428
−log10(p−value)
Recombination rate (cM/Mb)
FXR1→
SOX2−OT→
←DNAJC19
RNU6−2→
←LOC102724604
SOX2→
180.8
181
181.2
181.4
Position on chr3 (Mb)

G.

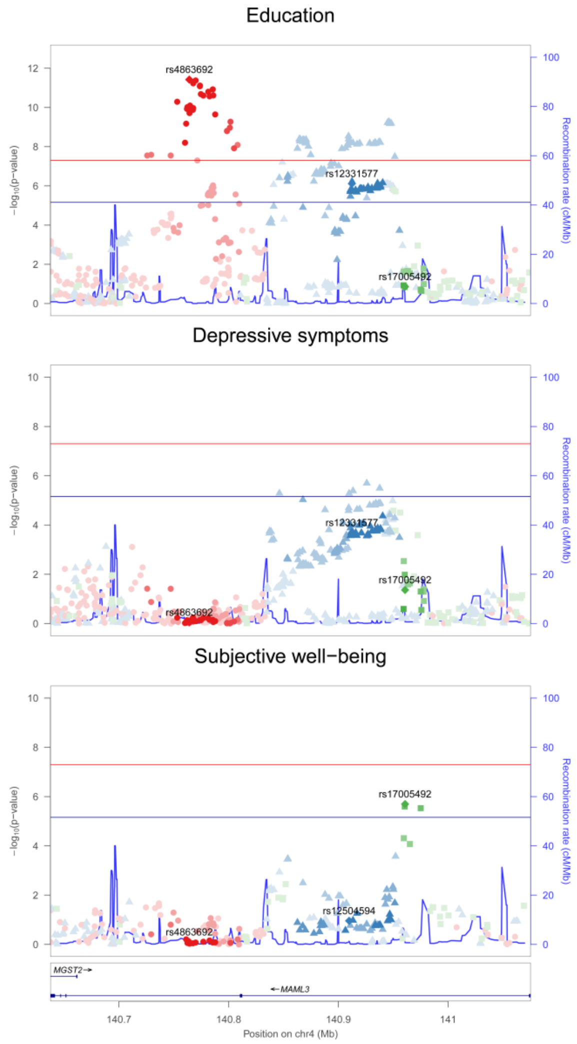
Education
Depressive symptoms
Subjective well−being
rs4863692
rs12331577
rs17005492
rs12504594
−log10(p−value)
Recombination rate (cM/Mb)
MGST2→
←MAML3
140.7
140.8
140.9
141
Position on chr4 (Mb)

H.

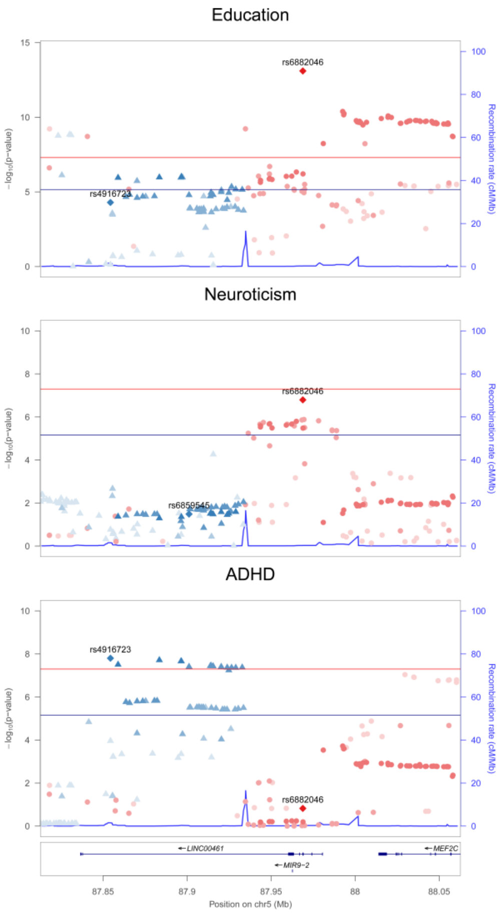

Education
rs6882046
rs4916723
−log10(p-value)
Recombination rate (cM/Mb)
Neuroticism
rs6882046
rs6859545
ADHD
rs4916723
rs6882046
LINC00461
MIR9-2
MEF2C
87.85
87.9
87.95
88
88.05
Position on chr5 (Mb)

I.

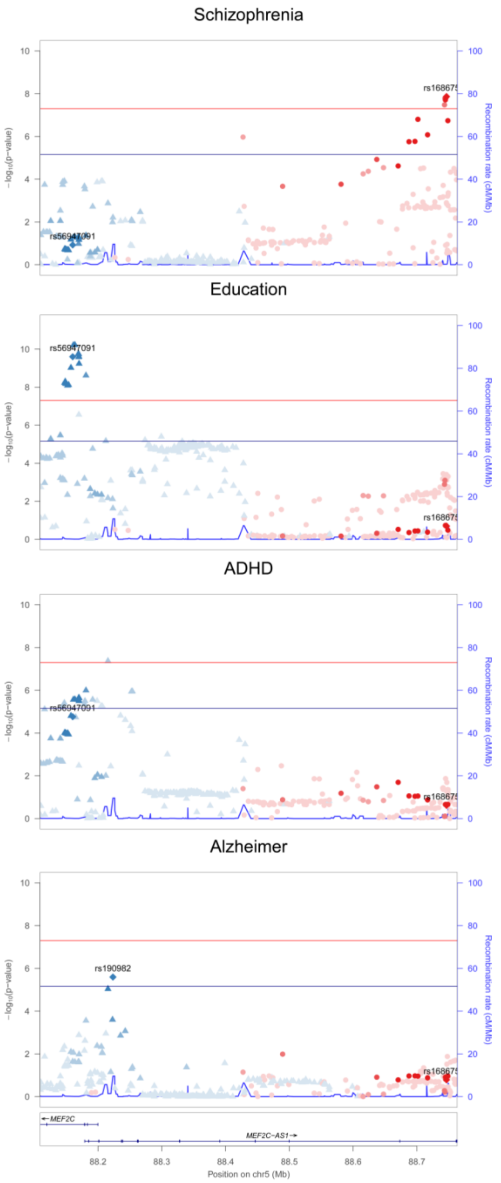
Schizophrenia
Education
ADHD
Alzheimer
rs56947091
rs190982
−log10(p-value)
Recombination rate (cM/Mb)
MEF2C
MEF2C-AS1
Position on chr5 (Mb)

J.

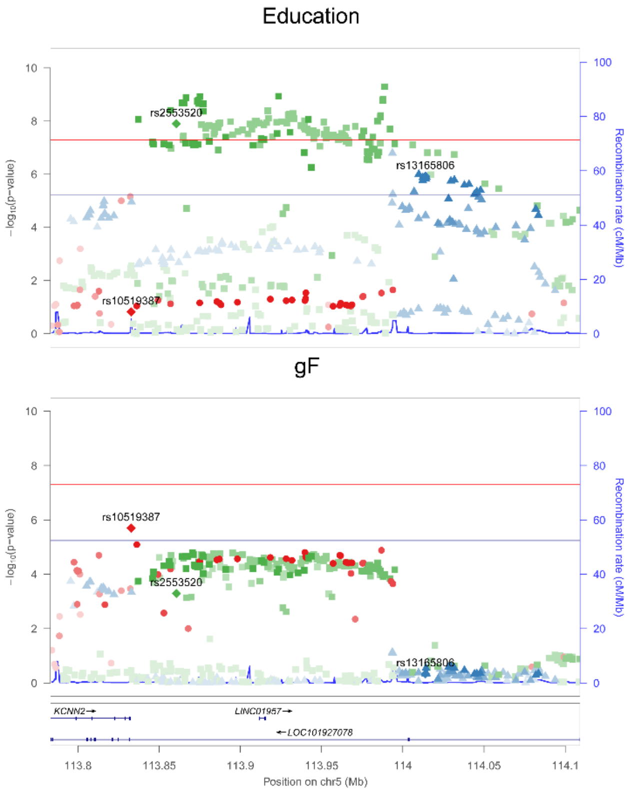
Education
gF
rs2553520
rs13165806
rs10519387
−log10(p-value)
Recombination rate (cM/Mb)
KCNN2
LINC01957
LOC101927078
Position on chr5 (Mb)

K.

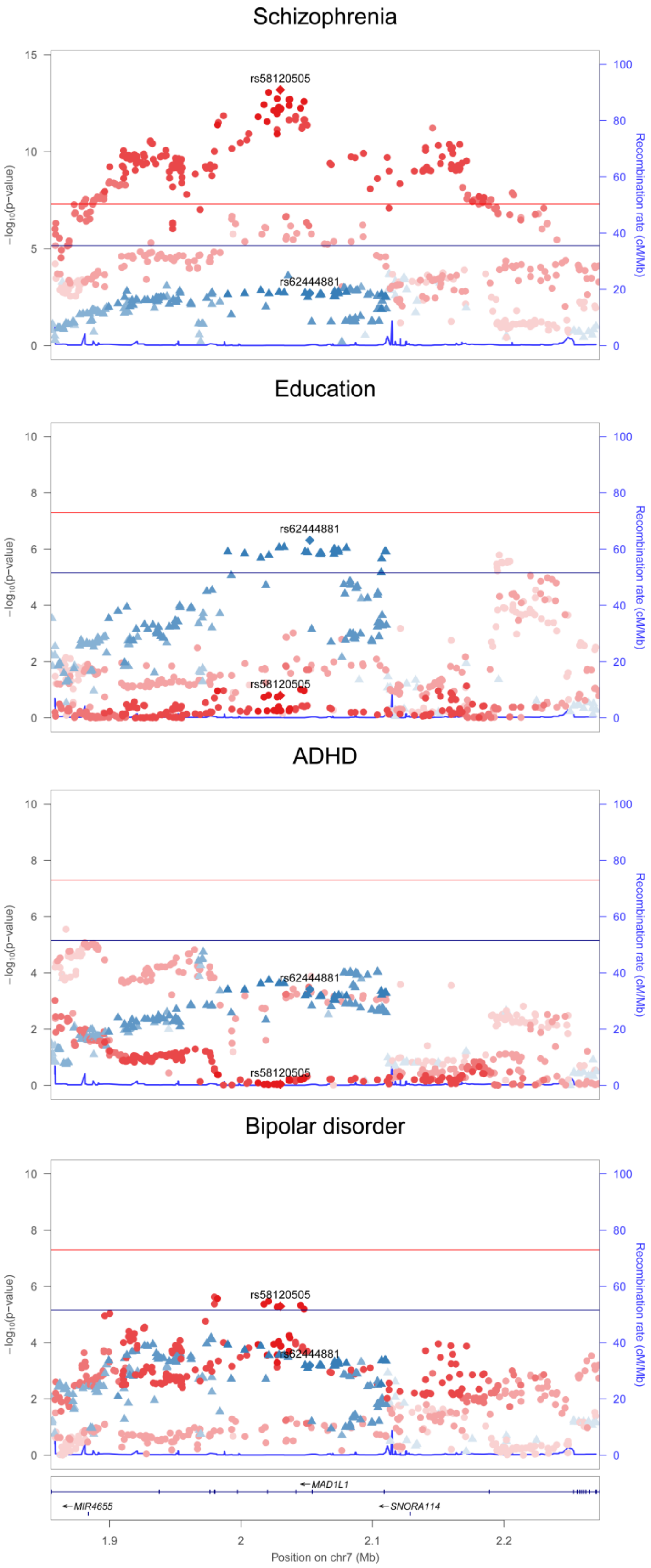
Schizophrenia
Education
ADHD
Bipolar disorder
rs58120505
rs62444881
−log10(p−value)
Recombination rate (cM/Mb)
MAD1L1
MIR4655
SNORA114
Position on chr7 (Mb)

L.

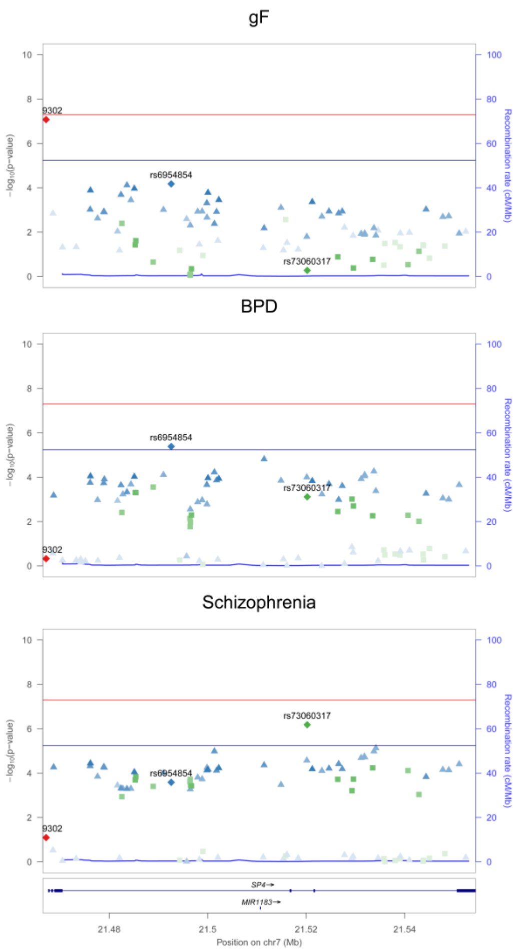
gF
BPD
Schizophrenia
rs6954854
rs73060317
9302
−log10(p-value)
Recombination rate (cM/Mb)
SP4
MIR1183
21.48
21.5
21.52
21.54
Position on chr7 (Mb)

M.

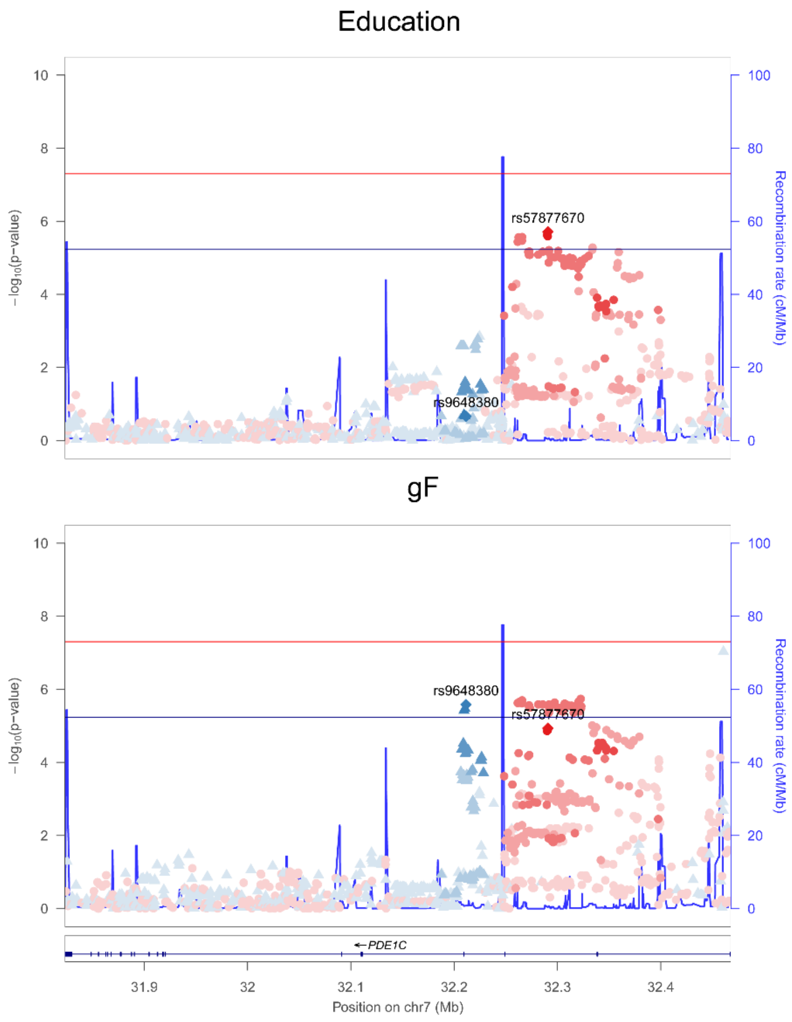
Education
gF
rs57877670
rs9648380
−log10(p-value)
Recombination rate (cM/Mb)
PDE1C
31.9
32
32.1
32.2
32.3
32.4
Position on chr7 (Mb)

N.

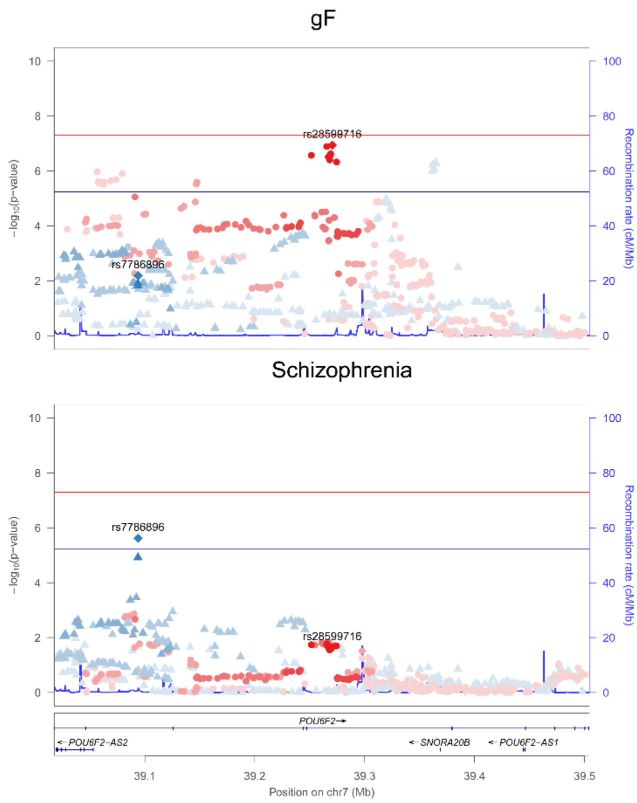
gF
rs28599716
rs7786896
−log10(p−value)
Recombination rate (cM/Mb)
Schizophrenia
rs7786896
rs28599716
POU6F2→
←POU6F2−AS2
←SNORA20B
←POU6F2−AS1
39.1
39.2
39.3
39.4
39.5
Position on chr7 (Mb)

O.

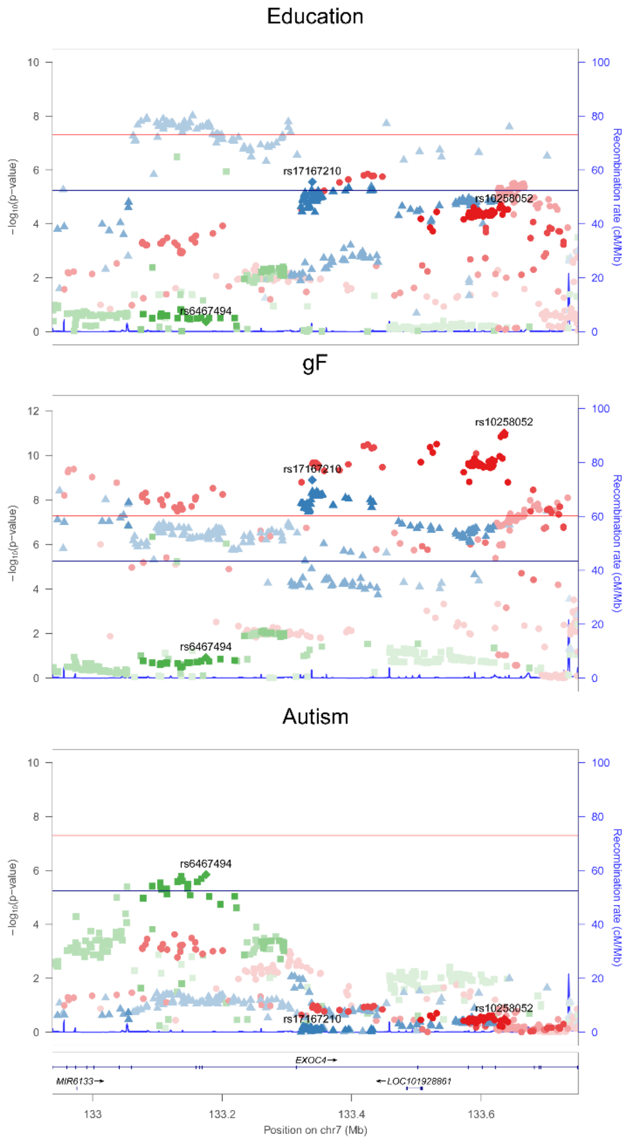
Education
rs17167210
rs10258052
rs6467494
−log10(p−value)
Recombination rate (cM/Mb)
gF
rs10258052
rs17167210
rs6467494
Autism
rs6467494
rs10258052
rs17167210
EXOC4→
MIR6133→
←LOC101928861
133
133.2
133.4
133.6
Position on chr7 (Mb)

P.

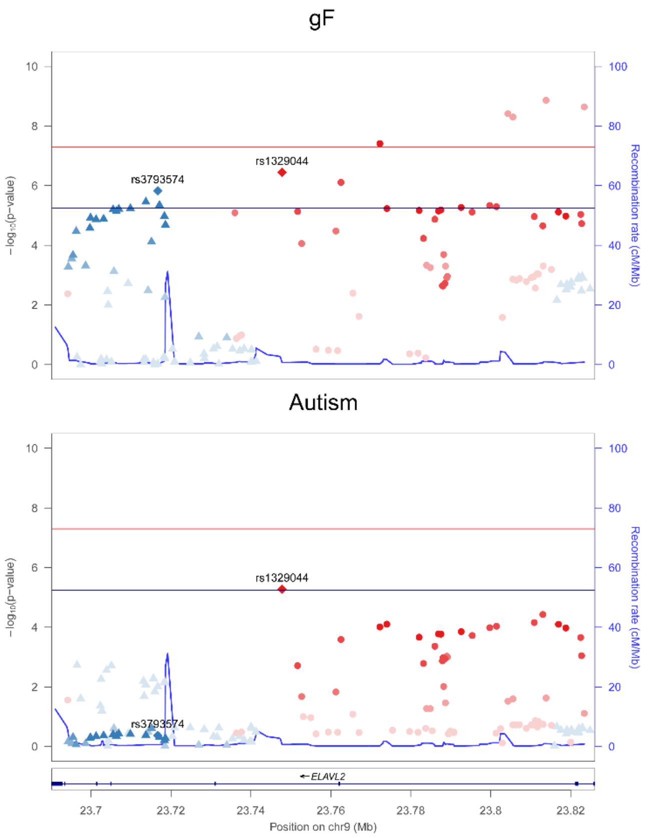
gF
rs1329044
rs3793574
Autism
rs1329044
rs3793574
−log10(p-value)
Recombination rate (cM/Mb)
ELAVL2
Position on chr9 (Mb)

Q.

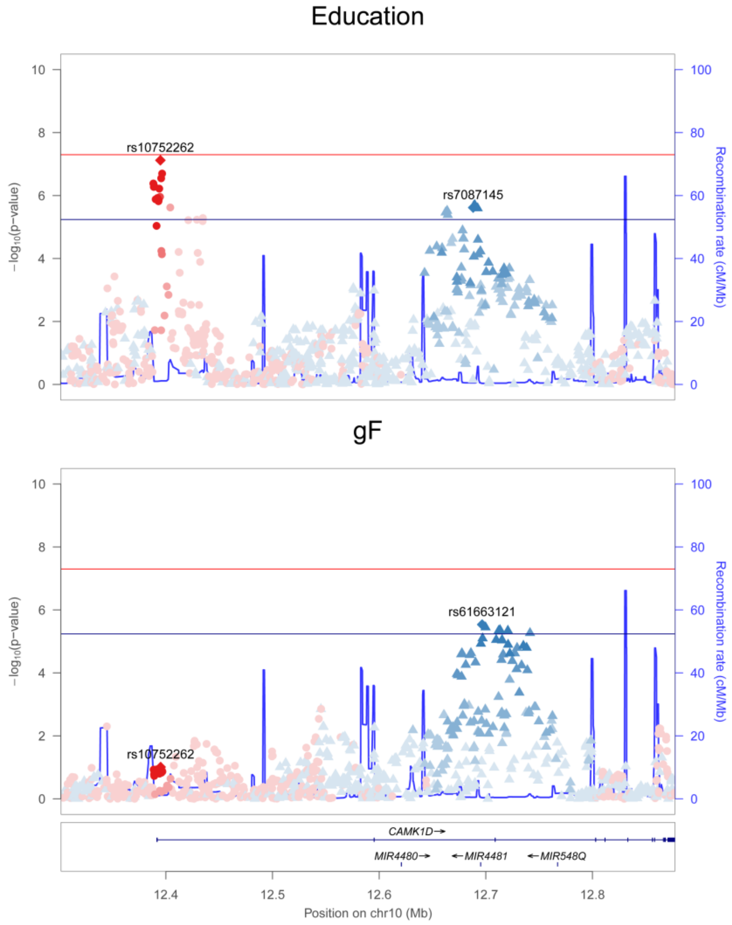
Education
rs10752262
rs7087145
gF
rs61663121
rs10752262
−log10(p-value)
Recombination rate (cM/Mb)
CAMK1D
MIR4480
MIR4481
MIR548Q
Position on chr10 (Mb)

R.

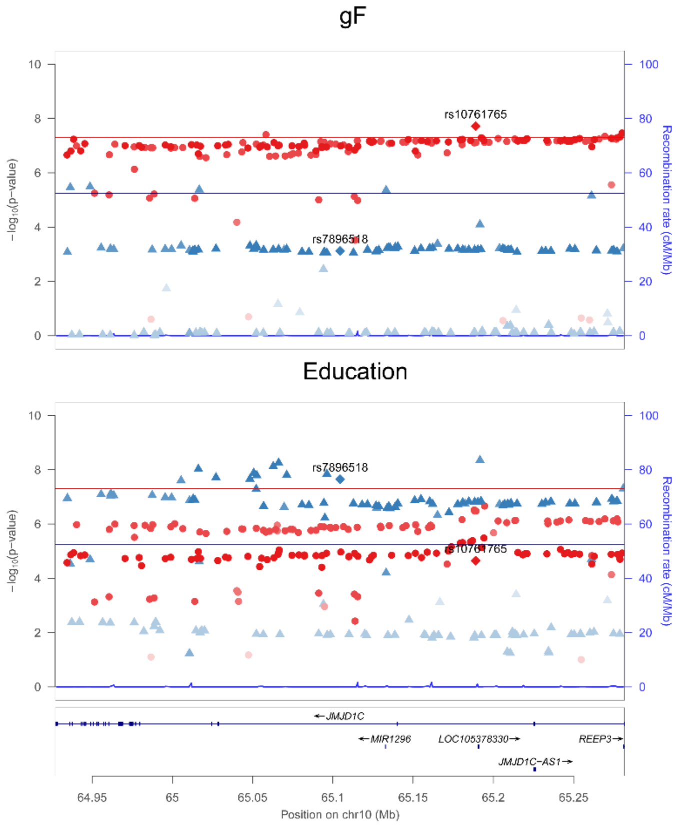
gF
rs10761765
rs7896518
−log10(p−value)
Recombination rate (cM/Mb)
Education
rs7896518
rs10761765
JMJD1C
MIR1296
LOC105378330
REEP3
JMJD1C−AS1
64.95
65
65.05
65.1
65.15
65.2
65.25
Position on chr10 (Mb)

S.

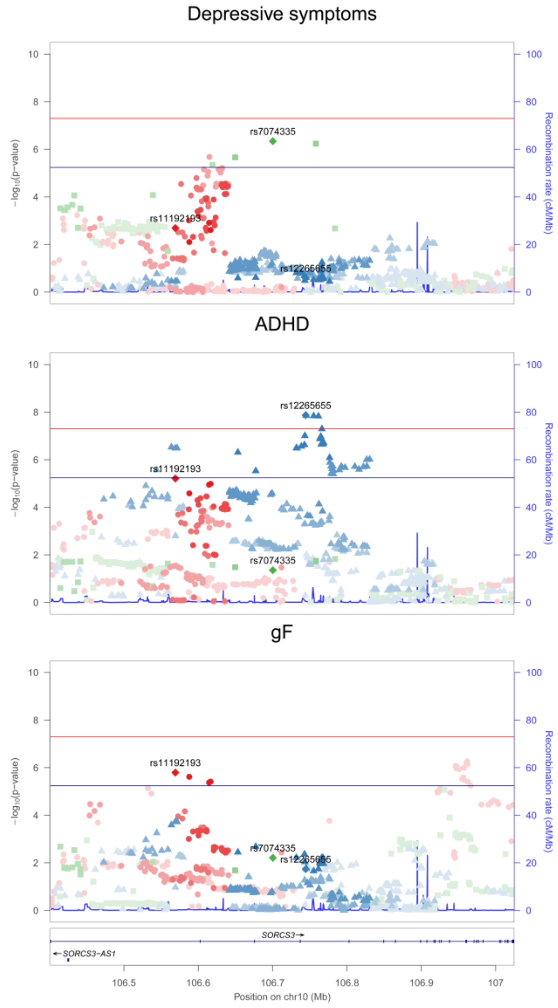
Depressive symptoms
rs7074335
rs11192193
rs12265655
−log10(p−value)
Recombination rate (cM/Mb)
ADHD
rs12265655
rs11192193
rs7074335
gF
rs11192193
rs7074335
rs12265655
SORCS3
SORCS3-AS1
106.5
106.6
106.7
106.8
106.9
107
Position on chr10 (Mb)

T.

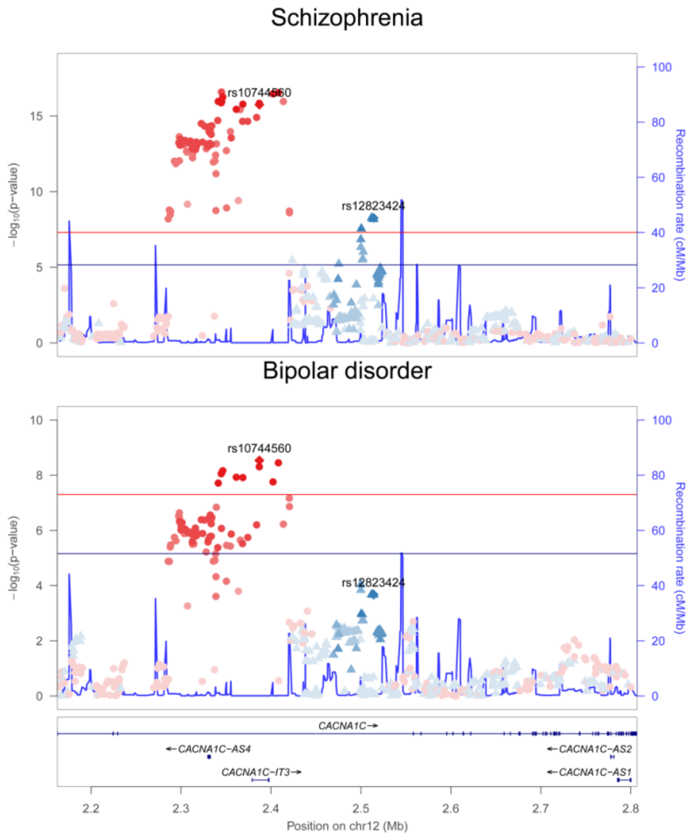
Schizophrenia
rs10744560
rs12823424
−log10(p−value)
Recombination rate (cM/Mb)
Bipolar disorder
rs10744560
rs12823424
−log10(p−value)
Recombination rate (cM/Mb)
CACNA1C→
←CACNA1C−AS4
CACNA1C−IT3→
←CACNA1C−AS2
←CACNA1C−AS1
Position on chr12 (Mb)

U.

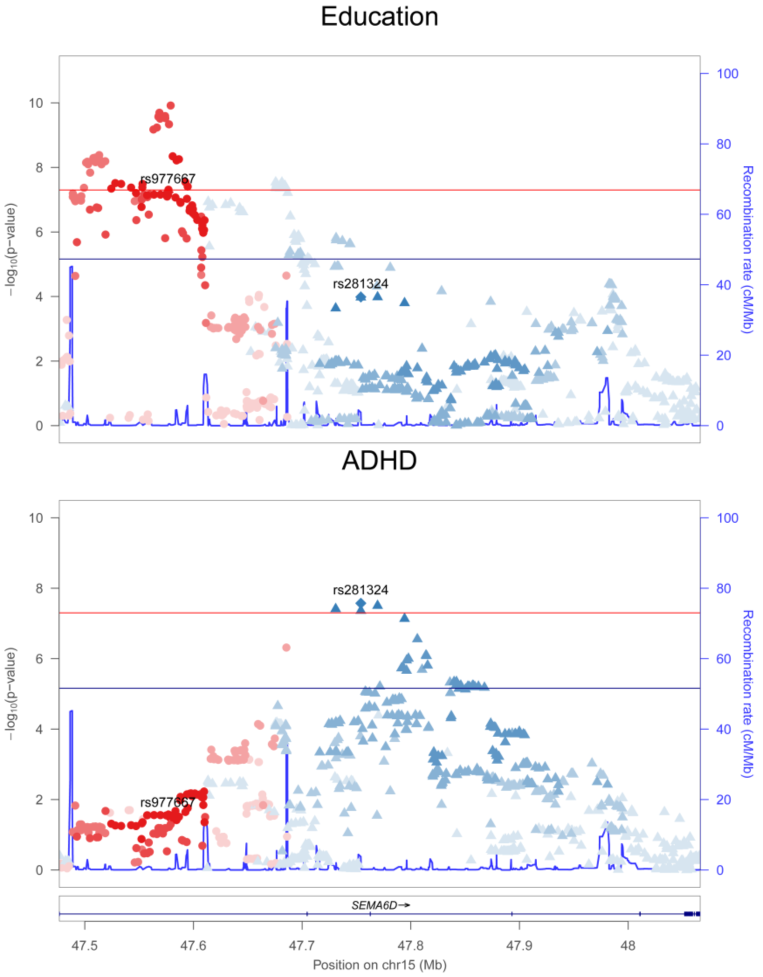
Education
rs977667
rs281324
−log10(p−value)
Recombination rate (cM/Mb)
ADHD
rs281324
rs977667
−log10(p−value)
Recombination rate (cM/Mb)
SEMA6D→
Position on chr15 (Mb)

V.

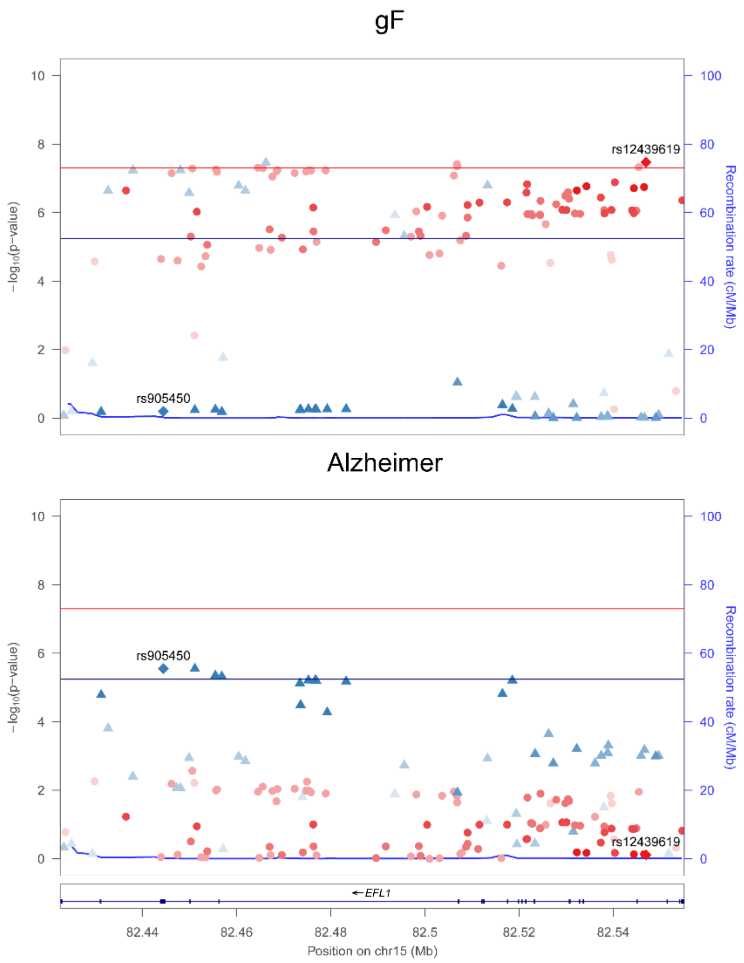

gF
10
8
6
4
2
0
−log10(p−value)
100
80
60
40
20
0
Recombination rate (cM/Mb)
rs12439619
rs905450
Alzheimer
10
8
6
4
2
0
−log10(p−value)
100
80
60
40
20
0
Recombination rate (cM/Mb)
rs905450
rs12439619
← EFL1
82.44
82.46
82.48
82.5
82.52
82.54
Position on chr15 (Mb)

W.
