# Supplementary material for: Identification of pleiotropy at the gene level between psychiatric disorders and related traits
Source: Transl Psychiatry. 2021 Jul 29;11:410. doi: 10.1038/s41398-021-01530-4 (PMC8322263; doi:10.1038/s41398-021-01530-4)
Supplement: Supplementary file 7 — Supplementary Figure 6 [file 41398_2021_1530_MOESM7_ESM.pdf]

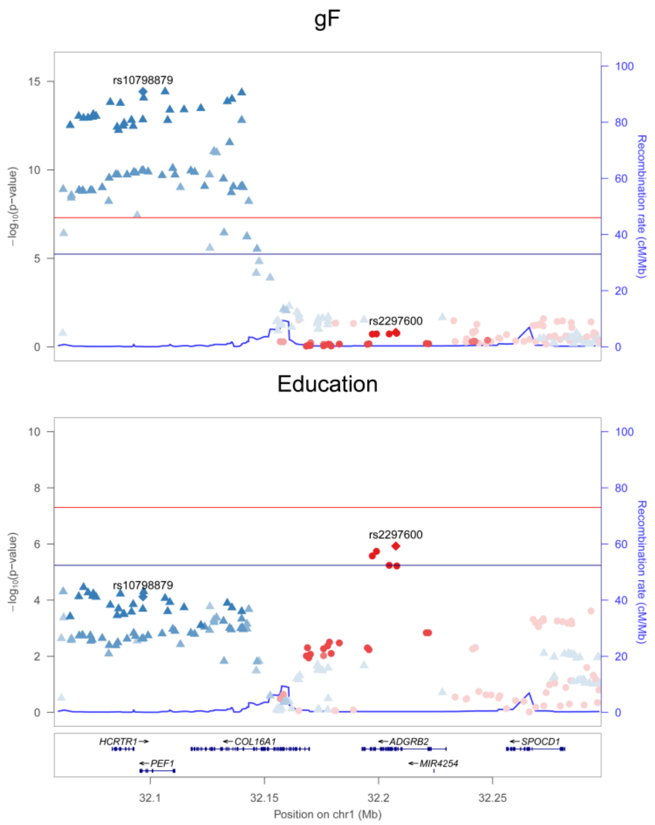
gF
rs10798879
rs2297600
Education
rs2297600
rs10798879
−log10(p−value)
Recombination rate (cM/Mb)
HCRTR1
COL16A1
ADGRB2
SPOCD1
PEF1
MIR4254
32.1
32.15
32.2
32.25
Position on chr1 (Mb)

A.

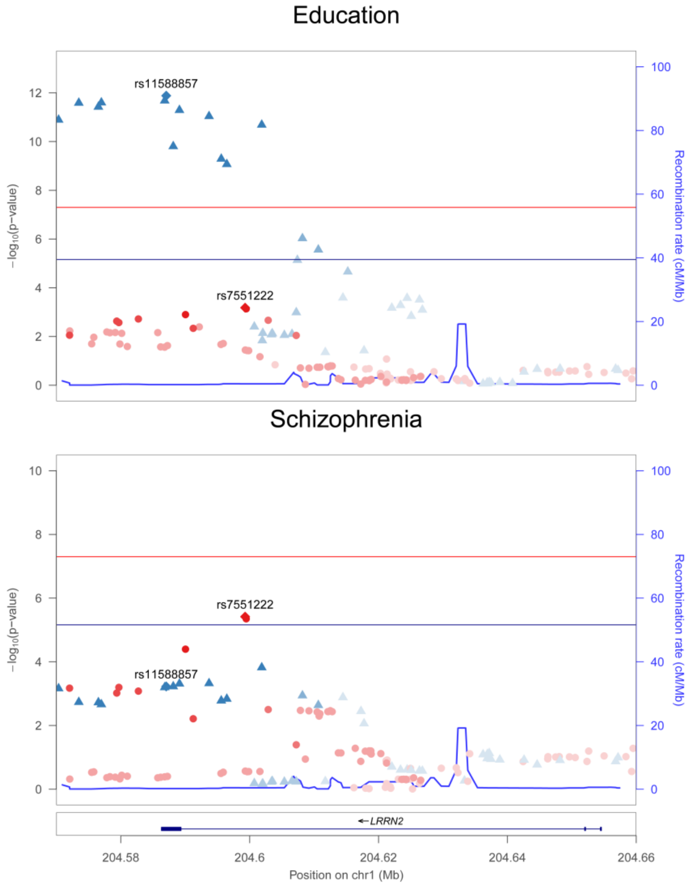
Education
rs11588857
rs7551222
Schizophrenia
rs7551222
rs11588857
−log10(p−value)
Recombination rate (cM/Mb)
LRRN2
204.58
204.6
204.62
204.64
204.66
Position on chr1 (Mb)

B.

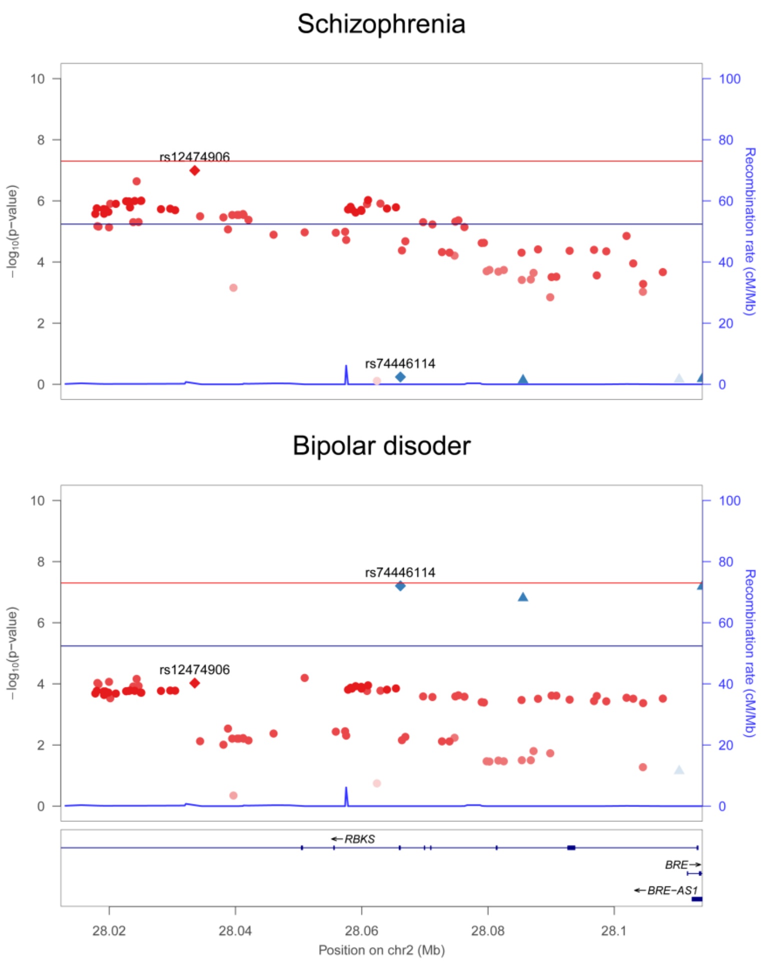
Schizophrenia
rs12474906
rs74446114
Bipolar disoder
rs74446114
rs12474906
−log10(p−value)
Recombination rate (cM/Mb)
RBKS
BRE
BRE−AS1
28.02
28.04
28.06
28.08
28.1
Position on chr2 (Mb)

C.

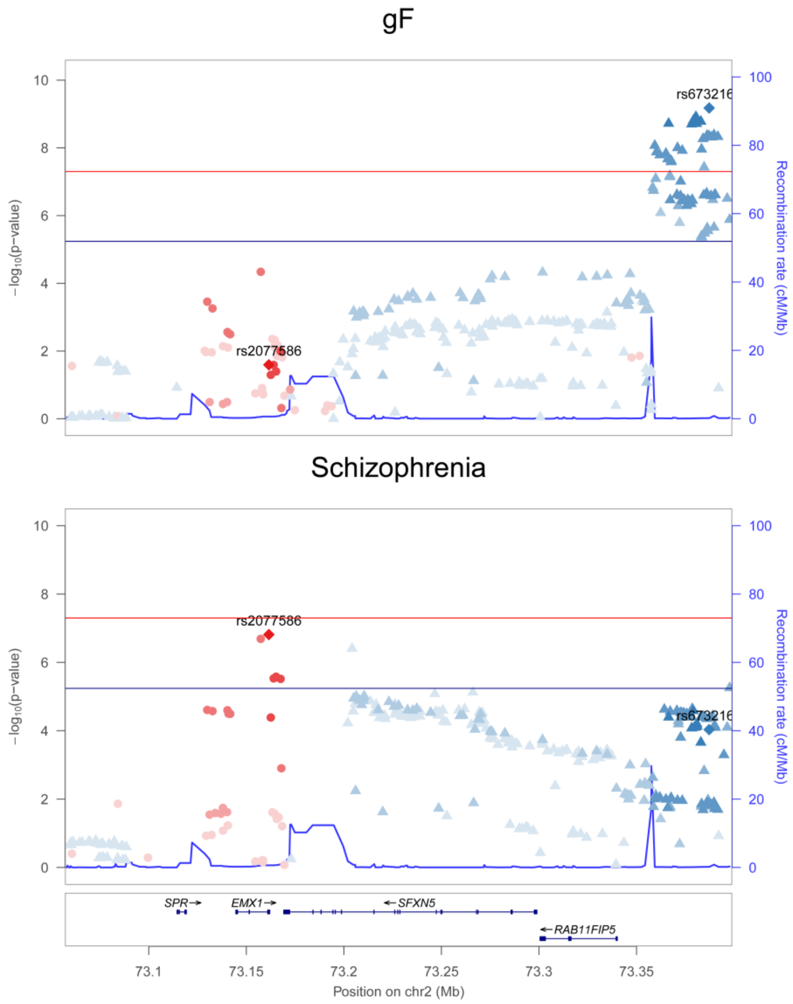
gF
rs673216
rs2077586
Schizophrenia
rs2077586
rs673216
−log10(p−value)
Recombination rate (cM/Mb)
SPR
EMX1
SFXN5
RAB11FIP5
73.1
73.15
73.2
73.25
73.3
73.35
Position on chr2 (Mb)

D.

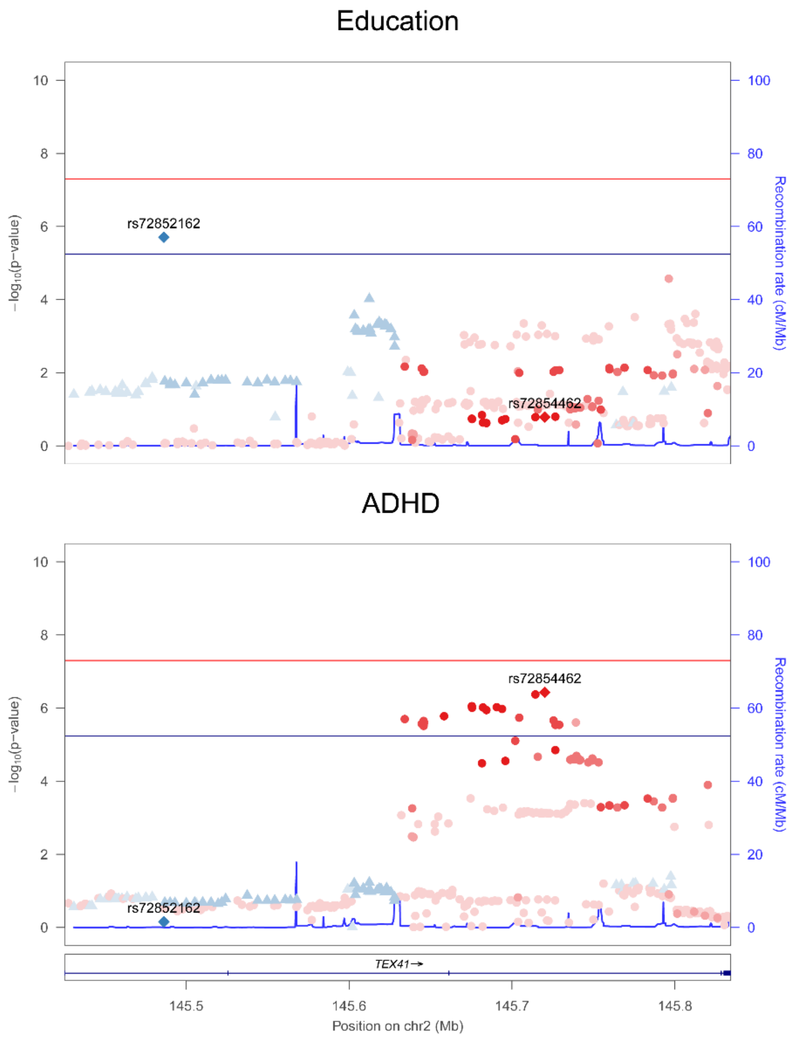
Education
rs72852162
rs72854462
ADHD
rs72854462
rs72852162
−log10(p-value)
Recombination rate (cM/Mb)
TEX41→
145.5
145.6
145.7
145.8
Position on chr2 (Mb)

E.

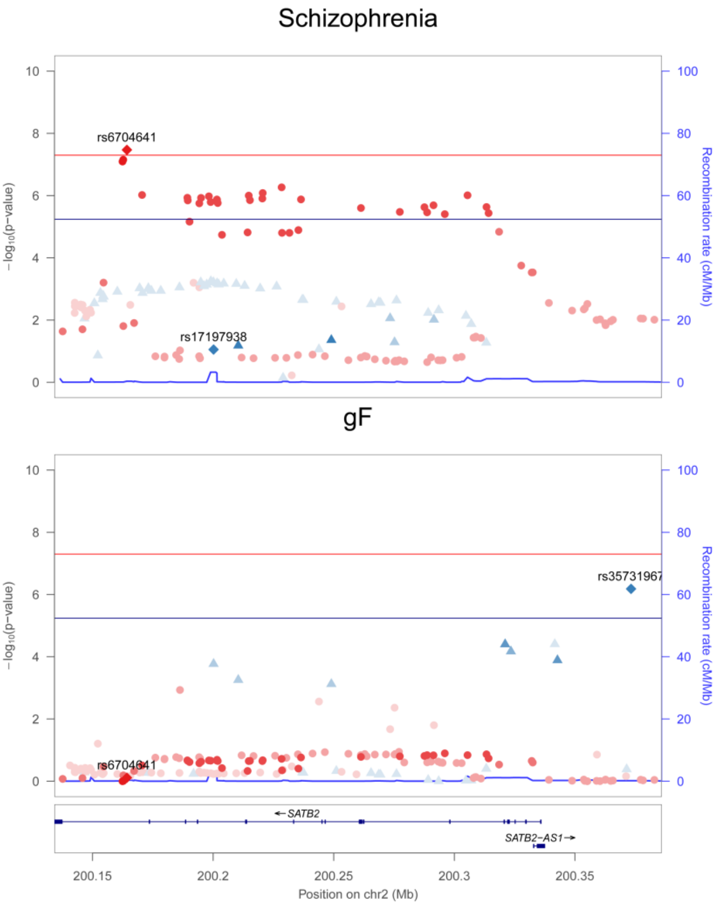
Schizophrenia
rs6704641
rs17197938
gF
rs35731967
rs6704641
−log10(p-value)
Recombination rate (cM/Mb)
←SATB2
SATB2−AS1→
200.15
200.2
200.25
200.3
200.35
Position on chr2 (Mb)

F.

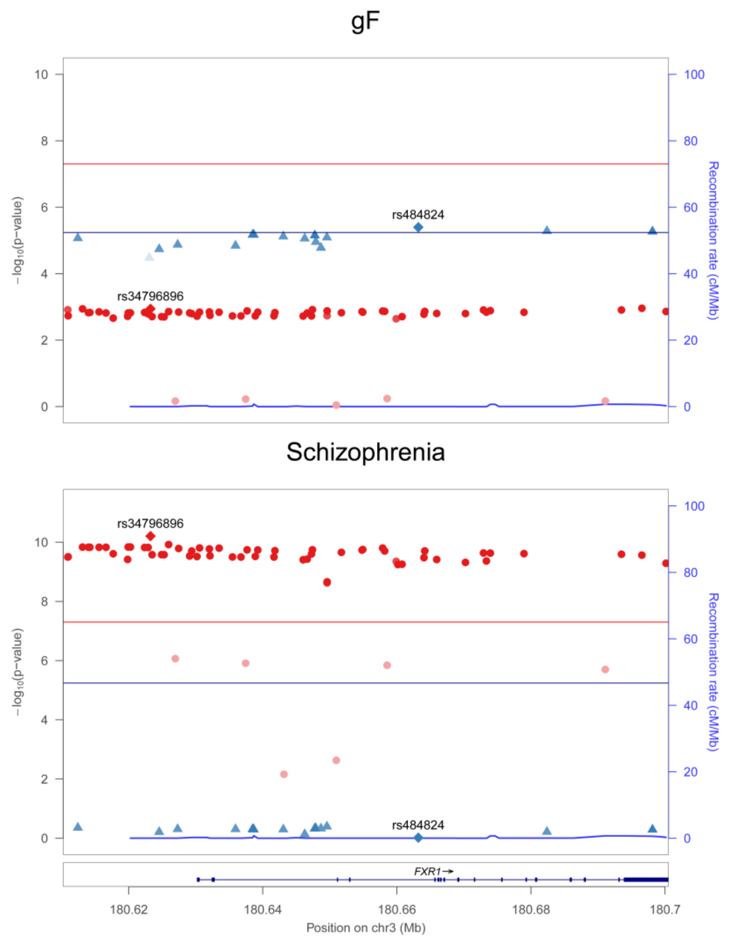
gF
rs484824
rs34796896
−log10(p−value)
Recombination rate (cM/Mb)
Schizophrenia
rs34796896
rs484824
FXR1→
180.62
180.64
180.66
180.68
180.7
Position on chr3 (Mb)

G.

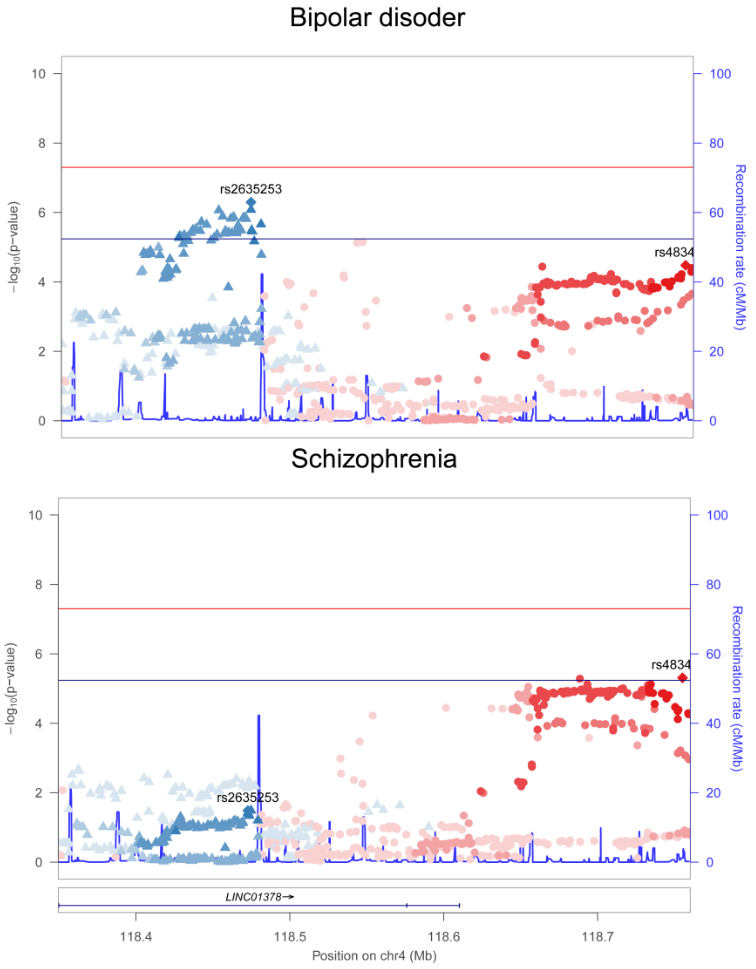
Bipolar disoder
rs2635253
rs4834
−log10(p−value)
Recombination rate (cM/Mb)
Schizophrenia
rs4834
rs2635253
LINC01378→
118.4
118.5
118.6
118.7
Position on chr4 (Mb)

H.

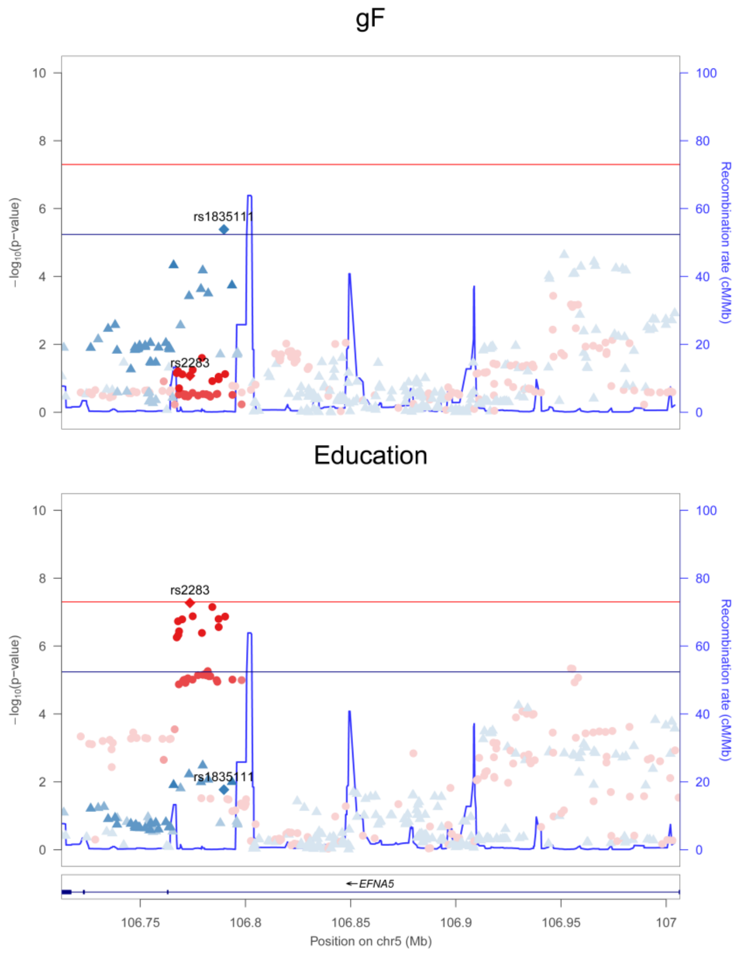
gF
rs1835111
rs2283
Education
rs2283
rs1835111
−log10(p−value)
Recombination rate (cM/Mb)
EFNA5
106.75
106.8
106.85
106.9
106.95
107
Position on chr5 (Mb)

I.

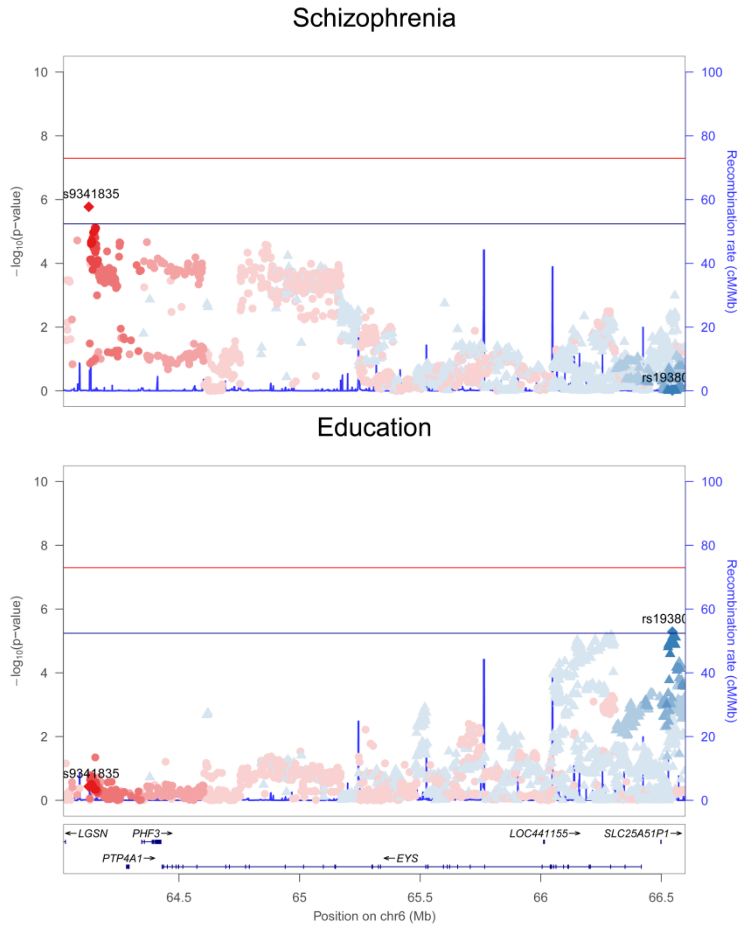
Schizophrenia
s9341835
Education
s9341835
−log10(p−value)
Recombination rate (cM/Mb)
LGSN
PHF3
PTP4A1
EYS
LOC441155
SLC25A51P1
64.5
65
65.5
66
66.5
Position on chr6 (Mb)

J.

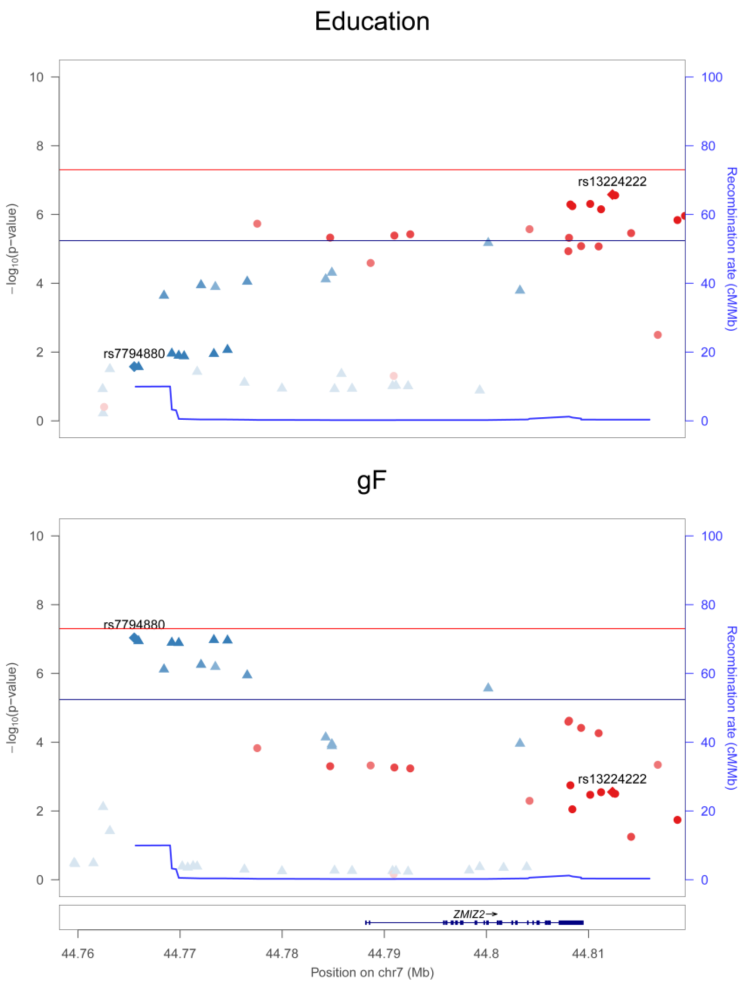
Education
gF
rs13224222
rs7794880
−log10(p−value)
Recombination rate (cM/Mb)
ZMIZ2
44.76
44.77
44.78
44.79
44.8
44.81
Position on chr7 (Mb)

K.

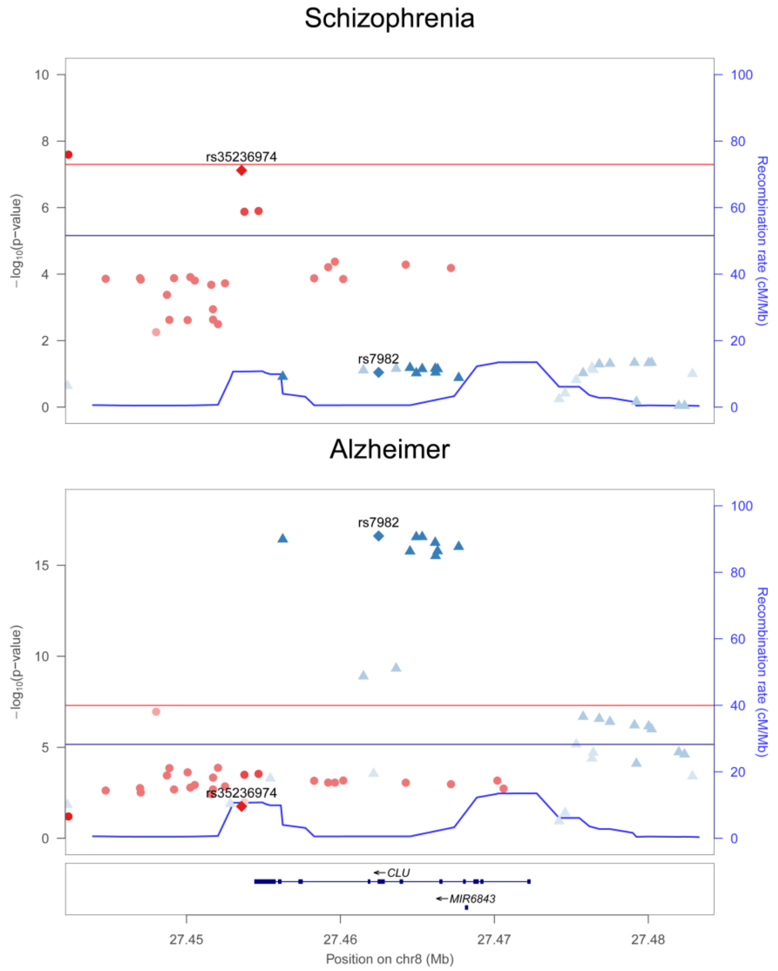
Schizophrenia
Alzheimer
rs35236974
rs7982
−log10(p−value)
Recombination rate (cM/Mb)
CLU
MIR6843
27.45
27.46
27.47
27.48
Position on chr8 (Mb)

L.

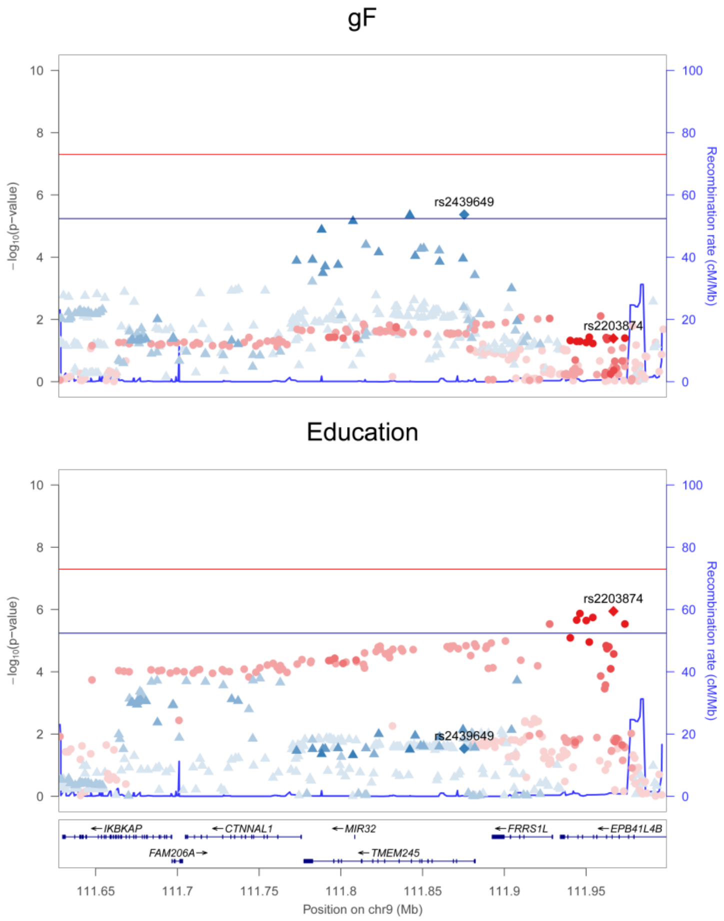
gF
Education
rs2439649
rs2203874
−log10(p−value)
Recombination rate (cM/Mb)
IKBKAP
CTNNAL1
MIR32
FRRS1L
EPB41L4B
FAM206A
TMEM245
111.65
111.7
111.75
111.8
111.85
111.9
111.95
Position on chr9 (Mb)

M.

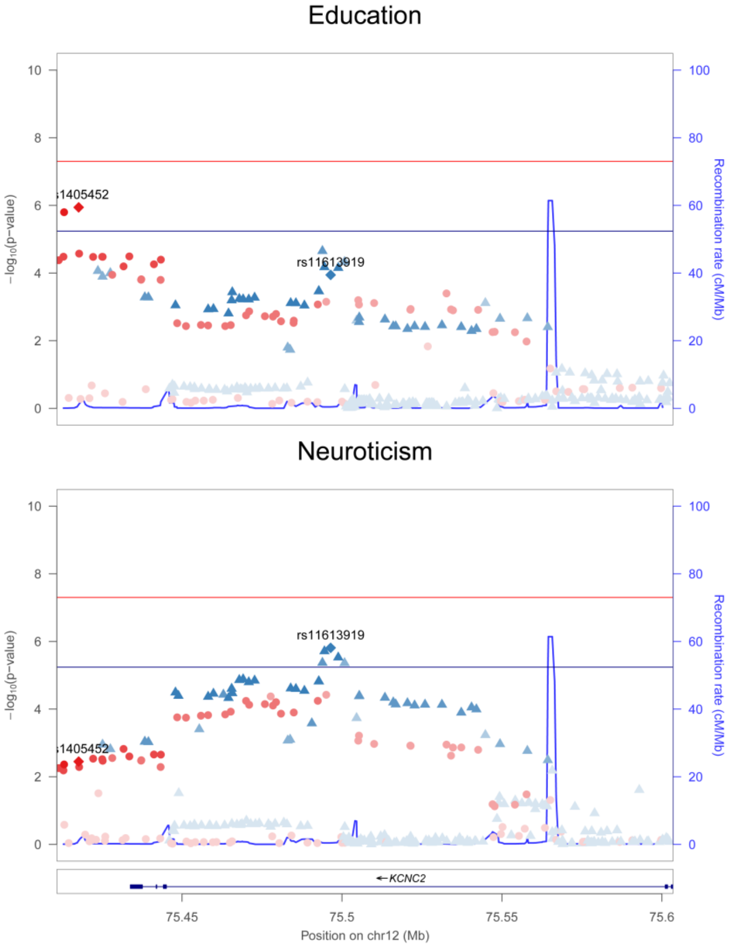
Education
Neuroticism
rs11613919
1405452
−log10(p−value)
Recombination rate (cM/Mb)
KCNC2
75.45
75.5
75.55
75.6
Position on chr12 (Mb)

N.

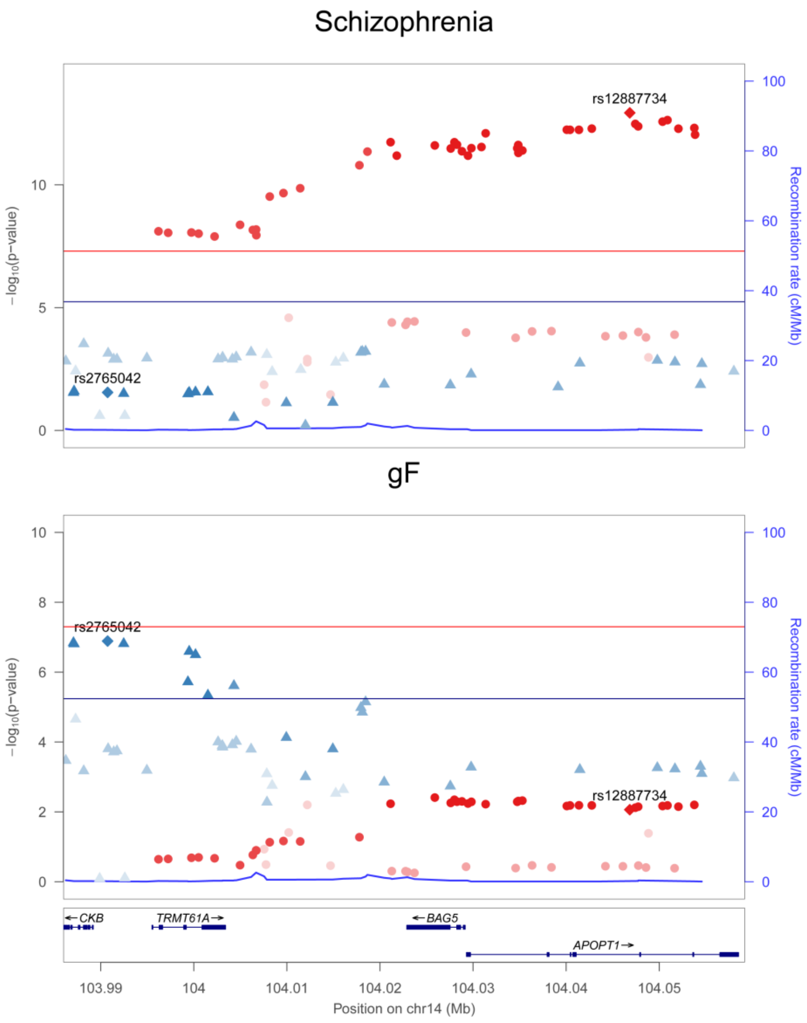
Schizophrenia
rs12887734
rs2765042
−log10(p-value)
Recombination rate (cM/Mb)
gF
rs2765042
rs12887734
CKB
TRMT61A
BAG5
APOPT1
103.99
104
104.01
104.02
104.03
104.04
104.05
Position on chr14 (Mb)

O.

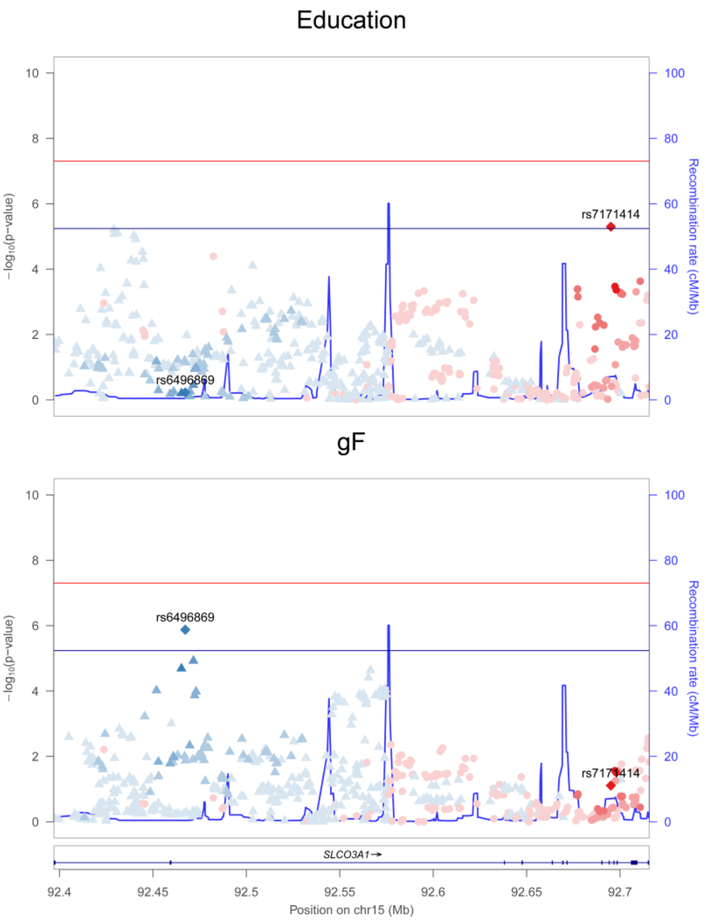
Education
rs7171414
rs6496869
−log10(p-value)
Recombination rate (cM/Mb)
gF
rs6496869
rs7171414
SLCO3A1
92.4
92.45
92.5
92.55
92.6
92.65
92.7
Position on chr15 (Mb)

P.

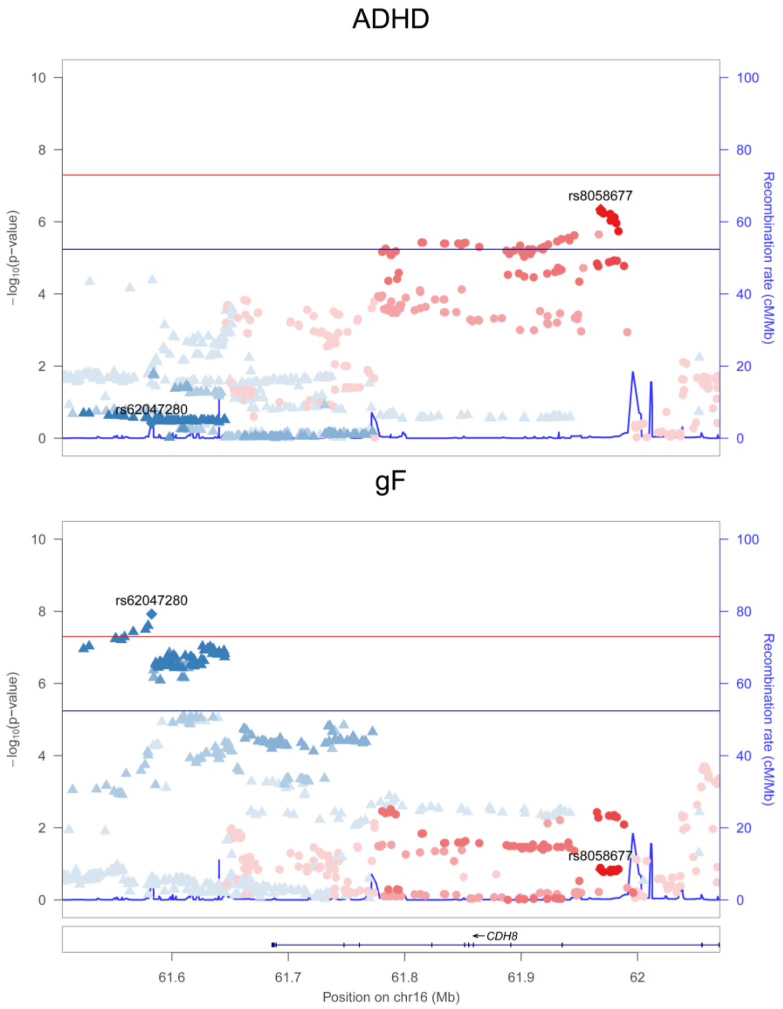
ADHD
gF
rs8058677
rs62047280
−log10(p−value)
Recombination rate (cM/Mb)
CDH8
61.6
61.7
61.8
61.9
62
Position on chr16 (Mb)

Q.

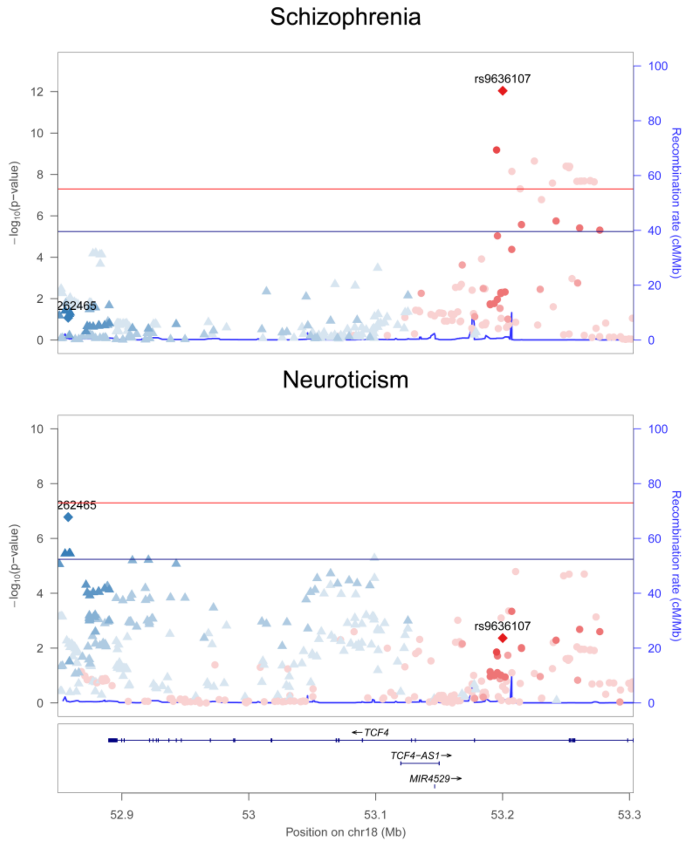
Schizophrenia
Neuroticism
rs9636107
262465
−log10(p−value)
Recombination rate (cM/Mb)
TCF4
TCF4−AS1
MIR4529
52.9
53
53.1
53.2
53.3
Position on chr18 (Mb)

R.

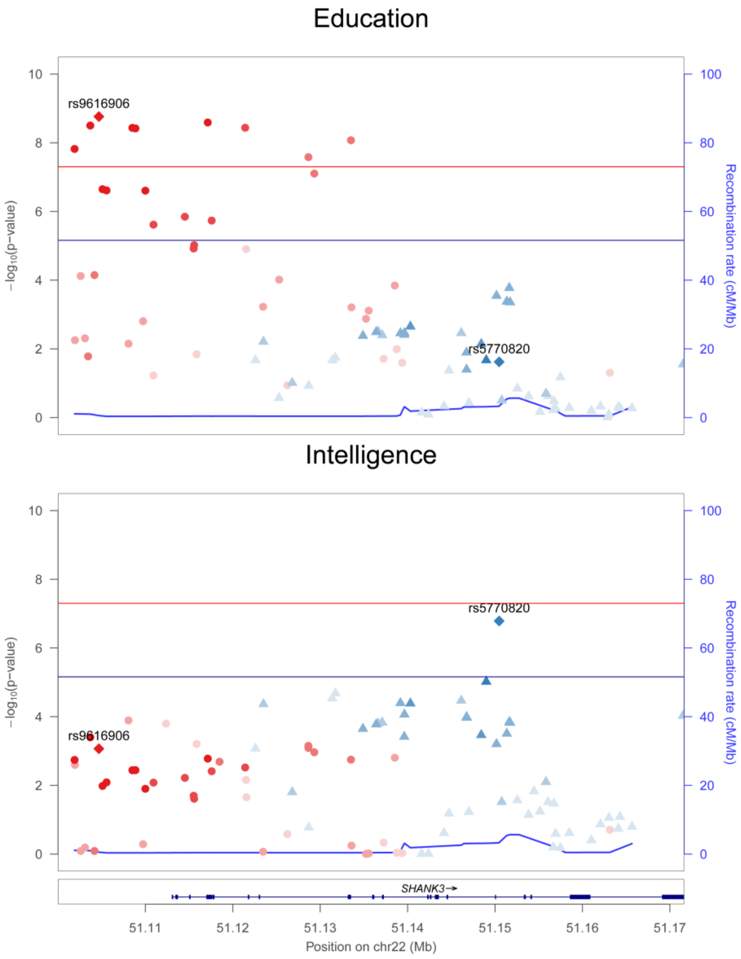

Education
rs9616906
rs5770820
−log10(p−value)
Recombination rate (cM/Mb)
Intelligence
rs5770820
rs9616906
SHANK3
51.11
51.12
51.13
51.14
51.15
51.16
51.17
Position on chr22 (Mb)

S.
