# Supplementary material for: Identification of pleiotropy at the gene level between psychiatric disorders and related traits
Source: Transl Psychiatry. 2021 Jul 29;11:410. doi: 10.1038/s41398-021-01530-4 (PMC8322263; doi:10.1038/s41398-021-01530-4)
Supplement: Supplementary file 8 — Supplementary Figure 7 [file 41398_2021_1530_MOESM8_ESM.pdf]

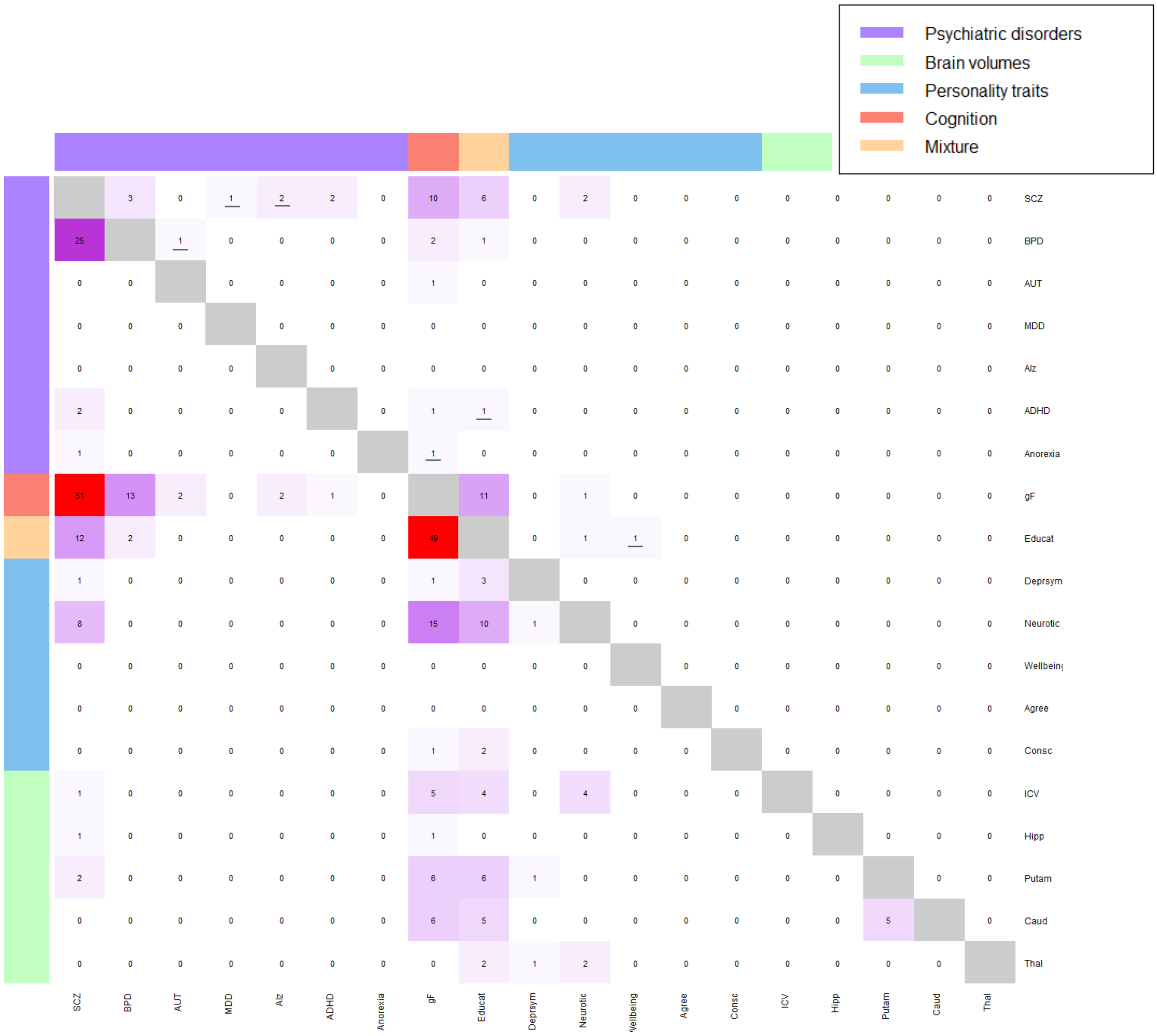

Number of genes with independent associations (Scenario I, upper triangle) and dependent associations (Scenario III, lower triangle) for each pair of traits. For a pair of traits, an underscore denotes that the gene-based overlap is bigger than the SNP-based overlap.

(*) Extraversion, anxiety, agreeableness, openness, aggression, loneliness, accumbens, pallidum and amygdala GWASs were excluded from the analysis, since their signals do not overlap with any other traits.

Alz – Alzheimer's disease, Intel – Intelligence, Educat – Educational attainment, Deprsym – Depressive symptoms, Neurotic – Neuroticism, Cons – Conscientiousness, ICV – Intracranial volume, Hipp – Hippocampus, Putam – Putamen, Caud – Caudate, Thal – Thalamus.
