# Supplementary material for: Identification of pleiotropy at the gene level between psychiatric disorders and related traits
Source: Transl Psychiatry. 2021 Jul 29;11:410. doi: 10.1038/s41398-021-01530-4 (PMC8322263; doi:10.1038/s41398-021-01530-4)
Supplement: Supplementary file 9 — Supplementary Figure 8 [file 41398_2021_1530_MOESM9_ESM.pdf]

	ICV	Hipp	Accum	Pal	Amyg	Putam	Caud	Thal
ICV	11	0	0	0	0	0	0	0
Hipp	0	10	0	0	0	0	0	0
Accum	0	0	8	0	0	0	0	0
Pal	0	0	0	8	0	1	0	0
Amyg	0	0	0	0	10	0	0	0
Putam	0	0	0	1	0	18	1	0
Caud	0	0	0	0	0	1	14	0
Thal	0	0	0	0	0	0	0	15
