# Supplementary material for: Identification of pleiotropy at the gene level between psychiatric disorders and related traits
Source: Transl Psychiatry. 2021 Jul 29;11:410. doi: 10.1038/s41398-021-01530-4 (PMC8322263; doi:10.1038/s41398-021-01530-4)
Supplement: Supplementary file 10 — Supplementary Figure 9 [file 41398_2021_1530_MOESM10_ESM.pdf]

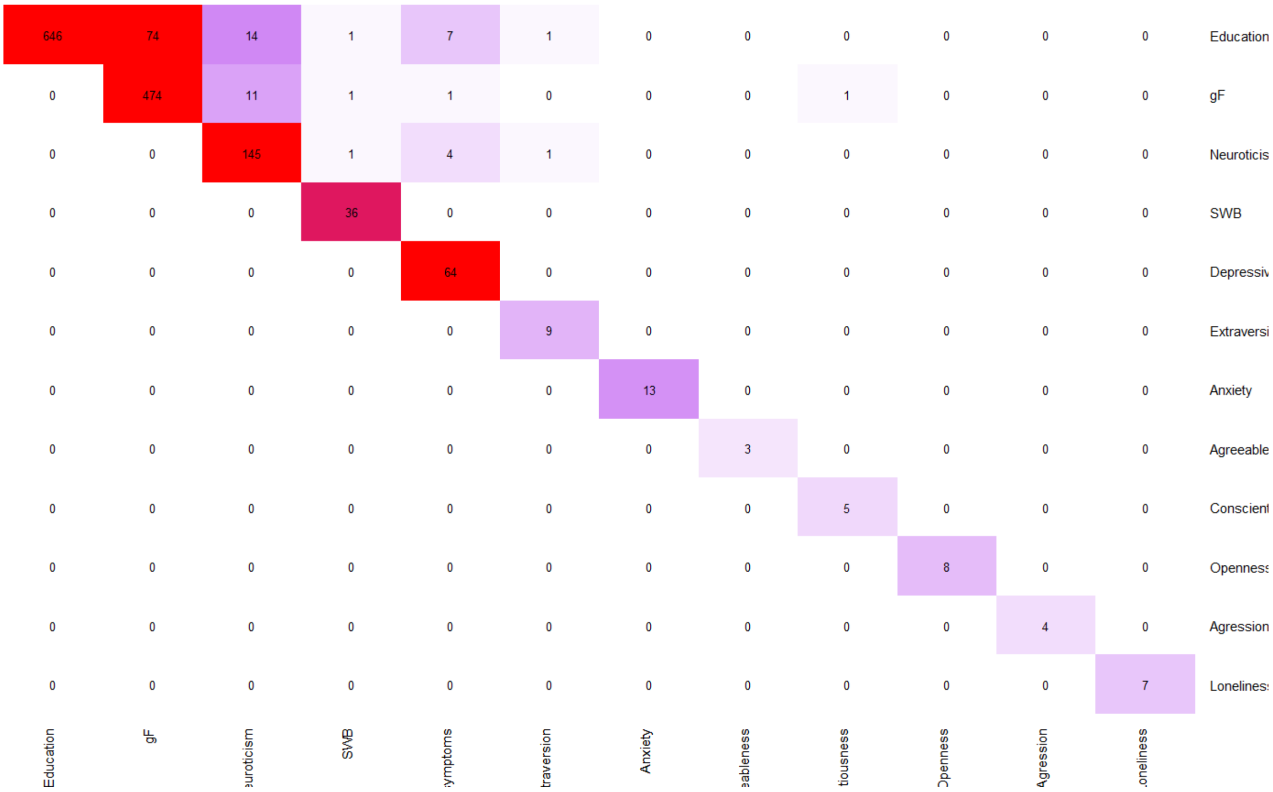
646 74 14 1 7 1 0 0 0 0 0 0 Education
0 474 11 1 1 0 0 0 1 0 0 0 gF
0 0 145 1 4 1 0 0 0 0 0 0 Neuroticis
0 0 0 36 0 0 0 0 0 0 0 0 SWB
0 0 0 0 64 0 0 0 0 0 0 0 Depressiv
0 0 0 0 0 9 0 0 0 0 0 0 Extraversi
0 0 0 0 0 0 13 0 0 0 0 0 Anxiety
0 0 0 0 0 0 0 3 0 0 0 0 Agreeable
0 0 0 0 0 0 0 0 5 0 0 0 Conscient
0 0 0 0 0 0 0 0 0 8 0 0 Openness
0 0 0 0 0 0 0 0 0 0 4 0 Agression
0 0 0 0 0 0 0 0 0 0 0 7 Lonelines
Education
gF
euroticism
SWB
ymptoms
traversion
Anxiety
eableness
tiousness
Openness
Agression
oneliness
