# Supplementary material for: Identification of pleiotropy at the gene level between psychiatric disorders and related traits
Source: Transl Psychiatry. 2021 Jul 29;11:410. doi: 10.1038/s41398-021-01530-4 (PMC8322263; doi:10.1038/s41398-021-01530-4)
Supplement: Supplementary file 11 — Supplementary Table 1 [file 41398_2021_1530_MOESM11_ESM.pdf]

Trait	Sample Size	Build	Frequency	Number of SNPs	Imputation score <0.9	Ambiguous markers	Indels	MAF<0.1	Final number of SNPs	below 10^{-5}	Independent markers <10-5 selected after conditional regression	PMID
**SCZ**	82,315	hg19	Calculated from 1000 genomes EUR	9,444,231	2,254,640	1,024,909	543.413	2,109,325	3,511,943	18.755	409	25056061
**Autism**	15,954	hg19	Frequency of A1 is provided	6,517,324	830.978	805.673	440.348	766.356	3,673,969	119	14	
**BPD**	51,710	hg19	Unaff_frq	13,414,632	5,976,560	1,061,507	530.623	2,385,153	3,460,789	3.665	135	31043756
**MDD**	18,759	*hg18*	CEU_frq	1,235,110	259.364	74.55	no data	168.062	733.133	16	5	22472876
**Alzheimer**	54,162	hg19	Calculated from 1000 genomes EUR	7,055,881	no data	1,051,453	no data	2,264,775	3,679,696	1.985	57	24162737
**ADHD**	55,374	hg19	Calculated from 1000 genomes EUR	8,047,420	691.02	1,029,646	5.727	2,725,753	3,595,274	1.929	77	29325848
**Anorexia**	14,477	hg19	Calculated from 1000 genomes EUR	13,694,841	6,129,145	1,077,505	538.548	2,230,624	3,508,450	170	10	24514567, 21079607
**ICV**	11,603	hg19	frq1 provided	8,700,908	no data	1,234,188	707.741	2,926,880	3,832,099	312	11	25607358
**Hippocampus**	11,603	hg19	frq1 provided	8,636,348	no data	1,222,741	717.675	2,846,989	3,848,943	89	10	25607358
**Accumbes**	11,603	hg19	frq1 provided	8,632,986	no data	1,222,296	717.334	2,842,016	3,851,340	17	8	25607358
**Palladium**	11,603	hg19	frq1 provided	8,630,495	no data	1,221,964	717.175	2,840,875	3,850,481	38	8	25607358
**Amygdala**	11,603	hg19	frq1 provided	8,612,778	no data	1,222,458	696.458	2,843,832	3,850,030	81	10	25607358
**Putamen**	11,603	hg19	frq1 provided	8,634,199	no data	1,222,436	717.435	2,844,739	3,849,589	837	18	25607358
**Caudate**	11,603	hg19	frq1 provided	8,636,889	no data	1,222,826	717.664	2,847,941	3,848,458	123	14	25607358
**Thalamus**	11,603	hg19	frq1 provided	8,641,328	no data	1,223,482	718.051	2,847,113	3,852,682	66	15	25607358
**Anxiety**	18,000	hg19	Freq_a1	6,330,995	no data	987.949	no data	1,295,732	4,047,314	76	13	26857599
**Education**	293,723	hg19	EAF from CEU, GBR and TSI	8,146,840	no data	1,254,949	no data	2,972,375	3,919,516	15.919	646	27225129
**Subjective well-being**	298,420	hg19	EAF	2,268,674	no data	350.114	no data	368.478	1,550,082	127	36	27089181
**Depressive symptoms**	161,460	hg19	EAF	6,524,474	no data	1,006,046	no data	1,635,133	3,883,295	470	64	27089181
**Neuroticism**	170,911	hg19	EAF	6,524,432	no data	1,006,044	no data	1,782,037	3,992,165	7.362	145	27089181
**Extraversion**	63,661	hg19	Maf from 1000 genomes, EUR ph1 v3	6,941,603	no data	1,080,685	no data	1,998,870	3,862,048	51	9	24828478
**Openness**	17,375	*hg18*	HapMap Maf	2,305,640	316.019	298.6	no data	301.157	1,367,494	25	8	21173776
**Agreebleness**	17,375	*hg18*	HapMap Maf	2,305,461	311.517	295.245	no data	299.274	1,378,560	9	3	21173776
**Conscientiousn[illegible]**	17,375	*hg18*	HapMap Maf	2,305,682	308.209	292.906	no data	296.836	1,367,566	85	5	21173776
**gF**	282,014	*hg19*	Calculated from 1000 genomes EUR	12,871,897	no data	1,922,572	no data	4,788,896	3,581,785	22.672	474	29844566
**Agression**	18,988	*hg18*	Calculated from 1000 genomes EUR	2,188,528	no data	338.219	no data	270.294	1,555,097	17	4	26087016
**Loneliness**	10,760	hg19	EUR_MAF provided	5,768,558	no data	872.296	no data	2,014,353	2,817,242	14	7	27629369

**Total # of SNPs**	**2 215**
**Unique SNPs**	**2 290**
**SNPs not in genes**	**775**
**Total genes**	**1 410**
**Genes with one SNP per gene**	**1 168**
**Genes with 2+ SNP per gene**	**242**
**Blocks with unique combination of SNPs**	**1 161**
**Blocks with one SNP**	**935**
**Blocks with 2+ SNPs**	**226**

Columns: Trait – name of the trait; Sample Size – sample size of the study; Build – Genome build in downloaded data; Frequency – column name of the frequency in the original data, if frequency was provided. If frequency was not provided, it was calculated based on EUR sample from 1000 Genomes; Number of SNPs – number of SNPs in the original study; Imputation score <0.9 – number of SNPs that were filtered out based on imputation score, if imputation score is provided in the original data; Ambiguous markers – number of filtered ambiguous markers; Indels – number of filtered indels, if data is avaliable; MAF<0.1 – number of SNPs filtered based on MAF 10%; Final number of SNPs – number of SNPs after filtering. Below 10-5 – Number of SNPs that passed threshold 10-5; Independent markers <10-5 selected after conditional
