# Supplementary material for: Identification of pleiotropy at the gene level between psychiatric disorders and related traits
Source: Transl Psychiatry. 2021 Jul 29;11:410. doi: 10.1038/s41398-021-01530-4 (PMC8322263; doi:10.1038/s41398-021-01530-4)
Supplement: Supplementary file 12 — Supplementary Table 2 [file 41398_2021_1530_MOESM12_ESM.pdf]

Gene	Coordinates	Trait 1	Trait 2
**SNP-based analysis**			
RERE; LOC102724552	chr1:8412463-8877699	SCZ, rs301797, p = 2.724e-09, +	Education, rs301800, p = 2.433e-07, +
CACNA1E	chr1:181452685-181775921	gF, rs199928, p = 3.14e-06, +	Neurotic, rs4652676, p = 2.353e-06, +
RBKS	chr2:28004230-28113263	SCZ, rs12474906, p = 1.014e-07, +	BPD, rs2305929, p = 6.559e-08, -
CTNNA2	chr2:79740059-80875988	Education, rs191092, p = 3.153e-06, +	gF, rs2060396, p = 6.138e-07, -
LRP1B	chr2:140988995-142889270	gF, rs13034371, p = 7.677e-08, +	Education, rs16846463, p = 6.992e-08, +; rs2012174, p = 1.491e-06, +
ZNF385B	chr2:180306710-180726232	gF, rs10196283, p = 4.061e-06, -	SCZ, rs12478234, p = 1.982e-06, +
FHIT	chr3:59733003-61237135	SCZ, rs6780613, p = 1.13e-07, -	MDD, rs9830950, p = 1.236e-06, +
GPM6A	chr4:176554087-176923842	SCZ, rs1106568, p = 1.145e-08, -	Education, rs12640626, p = 8.683e-08, +
JADE2	chr5:133860065-133918920	Education, rs329120, p = 3.891e-07, +	BPD, rs329319, p = 1.539e-08, +
ATXN1	chr6:16299342-16761721	gF, rs180020, p = 1.495e-07, +	Education, rs2073514, p = 1.284e-07, +
ERICH1-AS1; DLGAP2	chr8:687604-1656642	SCZ, rs12681288, p = 1.564e-06, +	ADHD, rs1532744, p = 1.642e-06, -
EXT1	chr8:118811601-119124058	gF, rs7814022, p = 5.058e-08, +	AUT, rs7836146, p = 3.324e-07, -
GLIS3	chr9:3824127-4300035	SCZ, rs1445349, p = 3.809e-06, -	Neurotic, rs9298995, p = 1.11e-06, -
ASTN2	chr9:119187503-120177317	BPD, rs1572156, p = 4.814e-06, -	AUT, rs7026354, p = 3.736e-06, +
TEAD1	chr11:12695968-12966284	Education, rs11022505, p = 4.178e-07, -	Wellbeing, rs7952069, p = 5.66e-06, +
LRRC4C	chr11:40135750-41481186	Anorexia, rs1348001, p = 4.824e-06, -	gF, rs2939755, p = 5.415e-06, -
TENM4	chr11:78364327-79151695	BPD, rs11237821, p = 1.173e-08, +	gF, rs547292, p = 3.119e-06, +
MIPEPP3	chr13:21872263-21967061	gF, rs4770140, p = 1.247e-06, +	SCZ, rs9316337, p = 1.713e-06, -
RBFOX1	chr16:6069131-7763340	SCZ, rs12447542, p = 1.122e-06, +; rs8054347, p = 2.7	gF, rs7192025, p = 9.85e-09, -; rs9934041, p = 1.883e-09, -
SNX29	chr16:12070590-12668146	Education, rs11859542, p = 3.488e-07, +	gF, rs2457192, p = 1.091e-08, -
CDH2	chr18:25530926-25757410	gF, rs11083241, p = 1.153e-06, -	Education, rs62103084, p = 9.428e-07, +
MACROD2	chr20:13976145-16033841	BPD, rs6079463, p = 5.378e-08, -	gF, rs743134, p = 9.922e-11, -
PHACTR3	chr20:58152563-58422766	gF, rs6123924, p = 1.449e-06, +	SCZ, rs7266699, p = 4.892e-06, -
**Gene-based analysis**			
COL16A1	chr1:32117847-32169768	Education, rs2297600, p_min = 1.187e-06	gF, rs10798879, p_min = 3.777e-15
LRRN2	chr1:204586302-204654597	SCZ, rs7551222, p_min = 3.831e-06	Education, rs11588857, p_min = 1.314e-12
BRE-AS1	chr2:28112322-28113981	SCZ, rs12474906, p_min = 1.014e-07	BPD, rs74446114, p_min = 6.238e-08
SFXN5	chr2:73169164-73298965	SCZ, rs2077586, p_min = 1.534e-07	gF, rs6732160, p_min = 6.66e-10
TEX41	chr2:145425533-145834291	ADHD, rs72854462, p_min = 3.715e-07	Education, rs72852162, p_min = 1.97e-06
SATB2	chr2:200134222-200335989	SCZ, rs6704641, p_min = 3.397e-08	gF, rs35731967, p_min = 6.572e-07
FXR1	chr3:180630233-180700539	SCZ, rs34796896, p_min = 6.234e-11	gF, rs484824, p_min = 4.004e-06
LINC01378	chr4:118349553-118610258	SCZ, rs4834639, p_min = 4.906e-06	BPD, rs2635253, p_min = 5.063e-07
EFNA5	chr5:106712589-107006596	Education, rs2283, p_min = 5.355e-08	gF, rs1835111, p_min = 4.112e-06
MAN2A1	chr5:109025066-109205326	SCZ, rs4388249, p_min = 1.025e-07	Education, rs1368357, p_min = 3.375e-06
EYS	chr6:64429875-66417118	SCZ, rs9341835, p_min = 1.687e-06	Education, rs1938060, p_min = 5.145e-06
ZMIZ2	chr7:44788164-44809479	Education, rs13224222, p_min = 2.635e-07	gF, rs7794880, p_min = 9.211e-08
CLU; MIR6843	**chr8:27454433-27472328**	**SCZ, rs73229090, p_min = 1.952e-08**	**Alz, rs7982, p_min = 2.477e-17**
TMEM245	chr9:111777414-111882225	Education, rs2203874, p_min = 1.136e-06	gF, rs2439649, p_min = 4.268e-06
KCNC2	chr12:75433857-75603528	Education, rs1405452, p_min = 1.142e-06	Neuroticism, rs11613919, p_min = 1.549e-06
CKB; TRMT61A; APOPT1	chr14:103985994-104058510	SCZ, rs12887734, p_min = 1.172e-13	gF, rs2765042, p_min = 1.289e-07
SLCO3A1	chr15:92396937-92715665	Education, rs7171414, p_min = 5.035e-06	gF, rs6496869, p_min = 1.333e-06
CDH8	chr16:61685914-62070739	ADHD, rs8058677, p_min = 4.543e-07	gF, rs62047280, p_min = 1.194e-08
TCF4	chr18:52889561-53303224	SCZ, rs9636107, p_min = 9.091e-13	Neuroticism, rs1262465, p_min = 1.66e-07
SHANK3	chr22:51113069-51171640	Education, rs9616906, p_min = 1.733e-09	gF, rs9616946, p_min = 7.058e-08

Column names: Gene - RefSeq gene names; coordinates - chromosome position (hg19); Trait 1 - trait name, rs id of selected in cojo-GCTA SNP, p-value of association with the Trait 1; Trait 2 - trait name, rs id of selected in cojo-GCTA SNP, p-value of association with the Trait 2; Trait 3 - trait name, rs id of selected in cojo-GCTA SNP, p-value of association with the Trait 3. Markers selected for Trait1, Trait2 and Trait3 are independent.

SCZ - schizophrenia; BPD - bipolar disoder; Alz - Alzheimer

Genes highlighted with bold, have genome-wide significant associations with 2+ traits
