# Supplementary material for: Identification of pleiotropy at the gene level between psychiatric disorders and related traits
Source: Transl Psychiatry. 2021 Jul 29;11:410. doi: 10.1038/s41398-021-01530-4 (PMC8322263; doi:10.1038/s41398-021-01530-4)
Supplement: Supplementary file 13 — Supplementary Table 3 [file 41398_2021_1530_MOESM13_ESM.pdf]

Gene	Coordinates	Trait 1
BSN	chr3:49591921-49708982	Education, rs4241405, p = 3.266e-08, +; rs4855852, p = 2.635e-06, -
NKAIN2	chr6:124124990-125146786	gF, rs12191379, p = 3.927e-06, +; rs6569363, p = 3.142e-06, +
RPS6KA2	chr6:166822851-167276039	BPD, rs9295358, p = 2.132e-06, +; rs960145, p = 4.634e-08, +
CDKAL1	**chr6:20534687-21232634**	**gF, rs4307160, p = 2.407e-09, +; rs9295486, p = 1.501e-08, -**
CTNNA3	chr10:67672275-69455949	Education, rs10996967, p = 3.844e-06, +; rs1925576, p = 2.472e-06, -
APOE region (NECTIN2, TOMM40, APOE, APOC1)	**chr19:45349392-45422606**	**Alz, rs10414043, p = 1.15e-522, +; rs760136, p = 1.69e-128, -**

Column names: SNPs selected within one gene are independent

Gene	RefSeq gene names
coordinates	chromosome position (hg19)
Trait 1	trait name, rsid of selected SNPs in cojo GCTA, pvalues

SCZ - schizophrenia; BPD - bipolar disoder; Alz - Alzheimer

Genes highlighted with bold, have genome-wide significant associations with 2+ traits
