# Supplementary material for: Identification of pleiotropy at the gene level between psychiatric disorders and related traits
Source: Transl Psychiatry. 2021 Jul 29;11:410. doi: 10.1038/s41398-021-01530-4 (PMC8322263; doi:10.1038/s41398-021-01530-4)
Supplement: Supplementary file 14 — Supplementary Table 4 [file 41398_2021_1530_MOESM14_ESM.pdf]

Gene	CHR	Trait 1	Trait 2	Trait3
**SNP-based analysis**				
PTPRF	**chr1:43991707-44089343**	**gF, rs2819336, p = 8.442e-11, +**	**Education, rs673253, p = 8.275e-10, +**	
ST3GAL3	**chr1:44173203-44396837**	**ADHD, rs17531412, p = 3.612e-13, +**	**gF, rs2906455, p = 5.5e-09, -**	**Education, rs2993621, p = 1.429e-07, +**
AK5	chr1:77747661-78025654	BPD, rs12751064, p = 7.9e-07, -	Education, rs12754946, p = 1.769e-06, +	
MIR137HG	chr1:98453555-98515249	SCZ, rs1702294, p = 2.794e-17, -	gF, rs2391903, p = 3.693e-06, +	
C1orf132	chr1:207974862-207996048	gF, rs2724373, p = 9.134e-07, +	SCZ, rs7523273, p = 1.609e-07, +	
BCL11A	**chr2:60678301-60780633**	**gF, rs10189857, p = 1.127e-09, +**	**SCZ, rs7599488, p = 3.114e-07, -**	**Education, rs7599488, p = 2.046e-11**
ALMS1	chr2:73612885-73837046	SCZ, rs56145559, p = 8.424e-08, +	gF, rs6546827, p = 2.224e-08, -	
ARHGAP15	**chr2:143886898-144525921**	**gF, rs13428598, p = 3.439e-09, +**	**Education, rs6430030, p = 2.049e-11, +**	
Block(TANK;LOC101929512;	chr2:161993465-162111154	Neuroticism, rs3754970, p = 4.741e-06, -	Education, rs7309, p = 3.026e-10, -	
DPP4	**chr2:162848754-162931052**	**Hippocampus, rs2268894, p = 2.213e-07, -**	**gF, rs2284871, p = 2.624e-12, +**	**SCZ, rs2909457, p = 4.375e-08, -**
CUL3	chr2:225334866-225450114	SCZ, rs11685299, p = 1.109e-08, -	Anorexia, rs72974222, p = 3.947e-06, -	
Block(ITIH3;ITIH4)	**chr3:52828783-52864717**	**BPD, rs2071044, p = 9.097e-09, -**	**SCZ, rs2535627, p = 3.956e-11, +**	
SLC39A8	chr4:103172197-103266410	gF, rs17032400, p = 8.258e-10, -	Consc, rs233826, p = 2.699e-06, -	
TET2.AS1	**chr4:106092510-106274038**	**gF, rs2726513, p = 4.927e-18, -**	**Education, rs2726518, p = 2.249e-10, +**	
MEF2C	**chr5:88014057-88199922**	**Education, rs304138, p = 2.643e-10, -**	**gF, rs42850, p = 1.691e-12, -**	
SLC22A23	chr6:3269206-3456793	Education, rs9503598, p = 1.462e-06, +	gF, rs9503599, p = 2.898e-07, -	
Block(NEU1;SLC44A4)	chr6:31826828-31846823	SCZ, rs2242665, p = 2.402e-07, +; rs2507954, p = 3.6	gF, rs494620, p = 5.435e-06, -	
EHMT2	chr6:31847535-31865484	SCZ, rs2242665, p = 2.402e-07, +	gF, rs494620, p = 5.435e-06, -	
ENPP5	chr6:46126918-46138747	gF, rs2143745, p = 2.789e-06, +	AUT, rs9472703, p = 6.133e-07, -	
FOXO3	chr6:108881025-109005971	ICV, rs3813498, p = 8.909e-07, +	SCZ, rs9398171, p = 3.373e-08, -	
CALN1	chr7:71244475-71877360	SCZ, rs2867673, p = 1.647e-06, +	gF, rs56150095, p = 5.894e-09, -	Education, rs6979866, p = 1.766e-06, +
SRPK2	**chr7:104756820-105029377**	**gF, rs1144, p = 1.008e-08, +**	**SCZ, rs3801278, p = 8.431e-09, -**	
FGFR1	chr8:38268655-38326352	SCZ, rs57709857, p = 2.324e-07, -	BPD, rs6984358, p = 2.131e-06, -	
TSNARE1	**chr8:143293440-143484610**	**gF, rs13262595, p = 2.688e-13, -**	**SCZ, rs4129585, p = 2.028e-13, +**	
APBA1	chr9:72042448-72287275	gF, rs1105307, p = 2.39e-06, -	Education, rs9775451, p = 9.646e-08, -	
MIR4481	chr10:12695136-12695196	gF, rs61663121, p = 2.909e-06, -	Education, rs7087145, p = 2.408e-06, +	
Block(BORCS7-ASMT;AS3MT)	chr10:104613966-104661655	SCZ, rs12244388, p = 1.087e-17, -	BPD, rs12764899, p = 5.632e-06, -	
MARK2	chr11:63606399-63678492	SCZ, rs4963432, p = 1.312e-06, +	BPD, rs4980532, p = 8.953e-07, -	
DLG2	chr11:83166055-85338314	SCZ, rs1864774, p = 5.477e-07, -	Putamen, rs6592145, p = 3.159e-08, -	
DISC1FP1	chr11:89984399-90648220	Education, rs10741373, p = 2.763e-07, +	gF, rs4753292, p = 3.97e-07, -	
ATF7IP	chr12:14518565-14655869	Education, rs10845998, p = 3.023e-11, +	gF, rs7978070, p = 2.286e-07, +	
Block(SUOX;LOC105369781)	**chr12:56391042-56418875**	**Education, rs10876864, p = 3.319e-09, -**	**gF, rs772921, p = 1.044e-10, +**	
ANKS1B	chr12:99128568-100378432	BPD, rs10745843, p = 3.411e-07, +	gF, rs61940242, p = 4.2e-07, -	SCZ, rs935143, p = 1.805e-06, +
MPHOSPH9	chr12:123640942-123717785	gF, rs1727301, p = 1.135e-06, -	SCZ, rs2102949, p = 2.416e-14, -	
FARP1	chr13:98794815-99102027	Thal, rs12261, p = 2.571e-06, +	Education, rs2281767, p = 4.677e-09, -	Neuroticism, rs7983438, p = 7.523e-07, +
STK24	chr13:99102452-99229405	Thal, rs12261, p = 2.571e-06, +	Education, rs2281767, p = 4.677e-09, -	Neuroticism, rs7983438, p = 7.523e-07, +
PRKD1	**chr14:30045685-30396948**	**SCZ, rs2068012, p = 4.142e-08, -**	**gF, rs3783300, p = 6.989e-10, -**	
AKAP6	chr14:32798478-33302268	ADHD, rs10133551, p = 5.098e-06, -	SCZ, rs12883788, p = 3.421e-07, +	gF, rs2239647, p = 5.774e-12, -
RTN1	chr14:60062693-60337557	SCZ, rs12431410, p = 4.217e-07, -	gF, rs35097543, p = 7.229e-08, +	
BCL11B	chr14:99635624-99738050	BPD, rs11624408, p = 3.642e-06, +	SCZ, rs2693698, p = 1.375e-08, -	
LINGO1	**chr15:77905365-78113242**	**Neuroticism, rs12903563, p = 2.864e-08, +**	**Education, rs3935685, p = 3.723e-08, -**	
GRIN2A	chr16:9847261-10276611	BPD, rs11647445, p = 1.217e-07, -	SCZ, rs9922678, p = 6.721e-09, +	
SH2B1	chr16:28857920-28885534	Caud, rs11150623, p = 4.907e-07, -	gF, rs62037363, p = 1.089e-18, +	
Block(LINC02210-CRHR1;CRI	**chr17:43697694-43913194**	**ICV, rs17689882, p = 2.855e-07, -**	**gF, rs4327090, p = 6.762e-11, +**	**Education, rs62057121, p = 2.8e-08, -**
DCC	**chr18:49866541-51062273**	**gF, rs11662271, p = 5.652e-13, +**	**SCZ, rs4632195, p = 4.186e-06, +**	**Putamenen, rs62097986, p = 3.242e-10, +**
SLC27A5	chr19:59009699-59023432	gF, rs12980430, p = 3.66e-07, -	BPD, rs28821001, p = 3.51e-06, -	
ARFGEF2	chr20:47538274-47653230	Education, rs4810893, p = 1.223e-06, -	Deprsym, rs4810896, p = 9.004e-07, -	
**Gene-based analysis**				
DPYD	**chr1:97543299-98386615**	**SCZ, rs1702294, p_min = 2.794e-17**	**Education, rs61785883, p_min = 1.771e-08**	**gF, rs2391903, p_min = 3.693e-06**
MIR2682; MIR137	**chr1:98510798-98511727**	**SCZ, rs1702294, p_min = 2.794e-17**	**Education, rs61785883, p_min = 1.771e-08**	

CD46; MIR29C; MIR29B2; LO	chr1:207925382-207995941	SCZ, rs7523273, p_min = 1.609e-07	gF, rs2724373, p_min = 9.134e-07	
CEP170; SDCCAG8	**chr1:243287729-243663393**	**SCZ, rs10803138, p_min = 1.788e-08**	**Education, rs71537331, p_min = 2.378e-10**	
PDIA6	chr2:10923516-10978103	Education, rs7590368, p_min = 5.288e-06	gF, rs116156133, p_min = 2.327e-06	
CNNM4; MIR3127	chr2:97426638-97477628	SCZ, rs7599624, p_min = 4.032e-06	BPD, rs4619651, p_min = 5.969e-09	
AFF3	**chr2:100163715-100759037**	**Education, rs12987662, p_min = 3.247e-18**	**gF, rs7583067, p_min = 4.079e-13**	
LONRF2	**chr2:100889752-100939195**	**Education, rs12987662, p_min = 3.247e-18**	**gF, rs2309812, p_min = 5.69e-13**	
ANKRD44; SF3B1	chr2:197851385-198299817	SCZ, rs6434928, p_min = 1.478e-11	gF, rs1064213, p_min = 2.544e-07	
FTCDNL1;TYW5	chr2:200625258-200820459	SCZ, rs796364, p_min = 5.89e-14	gF, rs3115423, p_min = 1.048e-06	
TRANK1	**chr3:36868307-36986548**	**SCZ, rs75968099, p_min = 3.386e-12**	**BPD, rs9834970, p_min = 5.531e-14**	
MST1R	**chr3:49924435-49941070**	**Education, rs2883059, p_min = 7.149e-25**	**gF, rs9873183, p_min = 4.551e-20**	
CACNA2D2	**chr3:50400043-50541675**	**Education, rs7630869, p_min = 7.644e-23**	**gF, rs7646366, p_min = 8.27e-18**	
ALAS1; STAB1; NT5DC2; SMI	**chr3:52232098-52719866**	**SCZ, rs2535627, p_min = 3.956e-11**	**BPD, rs2071044, p_min = 9.097e-09**	**gF, rs6778735, p_min = 1.194e-14**
MIR1284	chr3:71591120-71591240	SCZ, rs6803008, p_min = 3.288e-07	Education, rs7610856, p_min = 3.151e-07	gF, rs17663165, p_min = 1.171e-08
CD47	chr3:107761940-107809935	SCZ, rs9839167, p_min = 9.97e-05	BPD, rs3804640, p_min = 9.265e-08	Education, rs7640424, p_min = 1.044e-06
SLC9B2	chr4:103946647-103998528	SCZ, rs7688014, p_min = 4.264e-06	ADHD, rs227280, p_min = 2.422e-07	
TET2	**chr4:106067841-106200960**	**Education, rs6839705, p_min = 1.715e-11**	**gF, rs2726513, p_min = 4.927e-18**	
ETF1	chr5:137841781-137878989	SCZ, rs3849046, p_min = 4.833e-09	gF, rs409273, p_min = 3.885e-07	
HSPA9	chr5:137890570-137911318	SCZ, rs3849046, p_min = 4.833e-09	gF, rs409273, p_min = 3.885e-07	
LOC101929719	**chr5:139536903-139548370**	**Alz, rs2878896, p_min = 9.219e-08**	**gF, rs4463213, p_min = 7.96e-10**	
CYSTM1	**chr5:139554652-139623374**	**Alz, rs2878896, p_min = 9.219e-08**	**gF, rs4463213, p_min = 7.96e-10**	
BTN1A1	**chr6:26501494-26510652**	**SCZ, rs7746199, p_min = 3.35e-26**	**Neuroticismism, rs6904071, p_min = 7.587e-07**	**gF, rs9379900, p_min = 2.317e-14**
ABT1	**chr6:26597170-26600277**	**SCZ, rs7746199, p_min = 3.35e-26**	**Neuroticismism, rs6904071, p_min = 7.587e-07**	**gF, rs9379900, p_min = 2.317e-14**
HIST1H2BJ	**chr6:27100094-27100575**	**SCZ, rs7746199, p_min = 3.35e-26**	**Neuroticismism, rs6904071, p_min = 7.587e-07**	**gF, rs9379900, p_min = 2.317e-14**
ENPP4	chr6:46097700-46114436	AUT, rs7762549, p_min = 3.282e-07	gF, rs5011271, p_min = 1.582e-06	
CENPW	chr6:126660934-126804466	ICV, rs6918725, p_min = 7.165e-07	gF, rs9388487, p_min = 1.802e-07	
MPP6	chr7:24612964-24733322	SCZ, rs79210963, p_min = 1.382e-06	BPD, rs17150022, p_min = 2.704e-08	
DFNA5	chr7:24737973-24797639	SCZ, rs79210963, p_min = 1.382e-06	BPD, rs17150022, p_min = 2.704e-08	
LHFPL3	**chr7:103969103-104549003**	**SCZ, rs6466055, p_min = 2.463e-09**	**gF, rs1144, p_min = 1.008e-08**	
DGKI	**chr7:137072395-137531610**	**SCZ, rs3735025, p_min = 7.752e-09**	**Education, rs320700, p_min = 1.499e-09**	
FAM86B3P	**chr8:8086091-8102387**	**SCZ, rs2945232, p_min = 2.034e-08**	**Neuroticismism, rs2921036, p_min = 1.233e-14**	
ERI1	**chr8:8860313-8890849**	**SCZ, rs2945232, p_min = 2.034e-08**	**Neuroticismism, rs2921036, p_min = 1.233e-14**	
TNKS	chr8:9413444-9639856	SCZ, rs2048656, p_min = 1.451e-07	Neuroticismism, rs9329221, p_min = 6.627e-15	
XKR6	chr8:10753656-11058875	SCZ, rs12541491, p_min = 2.151e-07	Neuroticismism, rs2572431, p_min = 4.197e-16	
KIFC2	**chr8:145691719-145699499**	**Education, rs11774212, p_min = 2.91e-09**	**gF, rs750472, p_min = 3.679e-10**	
FOXH1	**chr8:145699114-145701718**	**Education, rs11774212, p_min = 2.91e-09**	**gF, rs750472, p_min = 3.679e-10**	
PPP1R16A	**chr8:145703364-145727504**	**Education, rs11774212, p_min = 2.91e-09**	**gF, rs750472, p_min = 3.679e-10**	
MFSD3	**chr8:145734418-145736611**	**Education, rs11774212, p_min = 2.91e-09**	**gF, rs750472, p_min = 3.679e-10**	
RECQL4	**chr8:145736666-145743210**	**Education, rs11774212, p_min = 2.91e-09**	**gF, rs750472, p_min = 3.679e-10**	
LRRC14	**chr8:145743348-145750559**	**Education, rs11774212, p_min = 2.91e-09**	**gF, rs750472, p_min = 3.679e-10**	
LRRC24	**chr8:145747760-145752416**	**Education, rs11774212, p_min = 2.91e-09**	**gF, rs750472, p_min = 3.679e-10**	
C8orf82	**chr8:145751602-145754458**	**Education, rs11774212, p_min = 2.91e-09**	**gF, rs750472, p_min = 3.679e-10**	
ARHGAP39	**chr8:145754562-145911197**	**Education, rs11774212, p_min = 2.91e-09**	**gF, rs750472, p_min = 3.679e-10**	
MGEA5	**chr10:103544199-103578222**	**Education, rs11191193, p_min = 6.967e-13**	**gF, rs1044258, p_min = 1.045e-13**	
KCNIP2	**chr10:103585730-103603677**	**Education, rs11191193, p_min = 6.967e-13**	**gF, rs1044258, p_min = 1.045e-13**	
C10orf76	**chr10:103605355-103815932**	**Education, rs11191193, p_min = 6.967e-13**	**gF, rs1044258, p_min = 1.045e-13**	
GBF1	**chr10:104005254-104142656**	**Education, rs11191193, p_min = 6.967e-13**	**gF, rs1044258, p_min = 1.045e-13**	
NT5C2	chr10:104847773-104953063	SCZ, rs12244388, p_min = 1.087e-17	Education, rs7101182, p_min = 7.55e-08	
PTPMT1	chr11:47586981-47595013	Neuroticismism, rs7107356, p_min = 1.265e-07	gF, rs3817334, p_min = 4.392e-09	
KBTBD4	chr11:47593748-47600567	Neuroticismism, rs7107356, p_min = 1.265e-07	gF, rs3817334, p_min = 4.392e-09	
NDUFS3	chr11:47600561-47606115	Neuroticismism, rs7107356, p_min = 1.265e-07	gF, rs3817334, p_min = 4.392e-09	
FAM180B	chr11:47608229-47610746	Neuroticismism, rs7107356, p_min = 1.265e-07	gF, rs3817334, p_min = 4.392e-09	
C1QTNF4	chr11:47611215-47615961	Neuroticismism, rs7107356, p_min = 1.265e-07	gF, rs3817334, p_min = 4.392e-09	

MTCH2	chr11:47638857-47664206	Neuroticismism, rs7107356, p_min = 1.265e-07	gF, rs3817334, p_min = 4.392e-09	
AGBL2	chr11:47681142-47736928	Neuroticismism, rs7107356, p_min = 1.265e-07	gF, rs3817334, p_min = 4.392e-09	
FNBP4	chr11:47738061-47789030	Neuroticismism, rs7107356, p_min = 1.265e-07	gF, rs3817334, p_min = 4.392e-09	
C11orf84	chr11:63580845-63595190	SCZ, rs4963432, p_min = 1.312e-06	BPD, rs4980532, p_min = 8.953e-07	
MTMR2	chr11:95566043-95657371	Education, rs523934, p_min = 1.089e-08	gF, rs523934, p_min = 2.582e-07	
RCOR2	chr11:63678692-63684316	SCZ, rs4963432, p_min = 1.312e-06	BPD, rs4980532, p_min = 8.953e-07	
CACNA1C-IT3	**chr12:2378941-2397911**	**SCZ, rs2007044, p_min = 2.625e-17**	**BPD, rs10744560, p_min = 2.918e-09**	
PTPRO	chr12:15475190-15751265	Education, rs10846170, p_min = 4.838e-06	gF, rs1318878, p_min = 1.122e-09	
PRKAG1	chr12:49396054-49412629	BPD, rs7969091, p_min = 3.248e-07	gF, rs10875914, p_min = 1.174e-10	
KMT2D	chr12:49412757-49449107	BPD, rs7969091, p_min = 3.248e-07	gF, rs10875914, p_min = 1.174e-10	
RHEBL1	chr12:49458458-49463808	BPD, rs7969091, p_min = 3.248e-07	gF, rs10875914, p_min = 1.174e-10	
LOC101928937	chr12:99487136-99498789	SCZ, rs660884, p_min = 1.701e-06	BPD, rs10745843, p_min = 3.411e-07	gF, rs61940242, p_min = 4.2e-07
NOVA1	chr14:26915088-27066960	Education, rs2224039, p_min = 3.413e-07	gF, rs1268446, p_min = 2.369e-06	
EXD2	chr14:69658193-69710737	Education, rs242093, p_min = 1.153e-06	gF, rs11158785, p_min = 1.308e-06	
HACD3	chr15:65822826-65870693	Education, rs12900061, p_min = 2.458e-10	gF, rs67089111, p_min = 1.824e-06	
ZNF592	**chr15:85291817-85349663**	**SCZ, rs950169, p_min = 7.62e-11**	**BPD, rs71395455, p_min = 1.934e-08**	
ATXN2L	**chr16:28834368-28848558**	**Putamen, rs4787452, p_min = 1.669e-05**	**Caud, rs11150623, p_min = 4.907e-07**	**Education, rs8049439, p_min = 2.686e-09**
TUFM	**chr16:28853731-28857729**	**Putamen, rs4787452, p_min = 1.669e-05**	**Caud, rs11150623, p_min = 4.907e-07**	**Education, rs8049439, p_min = 2.686e-09**
MIR4721	**chr16:28855239-28855328**	**Putamen, rs4787452, p_min = 1.669e-05**	**Caud, rs11150623, p_min = 4.907e-07**	**Education, rs8049439, p_min = 2.686e-09**
ATP2A1	**chr16:28889808-28915830**	**Putamen, rs4787452, p_min = 1.669e-05**	**Caud, rs11150623, p_min = 4.907e-07**	**Education, rs8049439, p_min = 2.686e-09**
ATP2A1-AS1	**chr16:28890277-28891242**	**Putamen, rs4787452, p_min = 1.669e-05**	**Caud, rs11150623, p_min = 4.907e-07**	**Education, rs8049439, p_min = 2.686e-09**
RAI1	**chr17:17584786-17714765**	**SCZ, rs8082590, p_min = 6.841e-09**	**gF, rs4925114, p_min = 1.265e-08**	
SREBF1	**chr17:17714662-17740325**	**SCZ, rs8082590, p_min = 6.841e-09**	**gF, rs4925114, p_min = 1.265e-08**	
MIR6777	**chr17:17716793-17716859**	**SCZ, rs8082590, p_min = 6.841e-09**	**gF, rs4925114, p_min = 1.265e-08**	
MIR33B	**chr17:17717149-17717245**	**SCZ, rs8082590, p_min = 6.841e-09**	**gF, rs4925114, p_min = 1.265e-08**	
GID4	**chr17:17942610-17971718**	**SCZ, rs8082590, p_min = 6.841e-09**	**gF, rs4925114, p_min = 1.265e-08**	
NSF	**chr17:44668034-44834828**	**ICV, rs17689882, p_min = 3.268e-07**	**Education, rs62057121, p_min = 2.8e-08**	**Neuroticismism, rs193236081, p_min = 6.264**
WNT3	**chr17:44839871-44896126**	**ICV, rs17689882, p_min = 3.268e-07**	**Education, rs62057121, p_min = 2.8e-08**	**Neuroticismism, rs193236081, p_min = 6.264**
KATNAL2	**chr18:44526786-44628614**	**Education, rs12962421, p_min = 1.495e-08**	**Consc, rs2576037, p_min = 4.908e-08**	
SKOR2	chr18:44738459-44775554	Education, rs12962421, p_min = 1.495e-08	Consc, rs2576037, p_min = 4.908e-08	
ZNF446	chr19:58988545-59000900	BPD, rs28821001, p_min = 3.51e-06	gF, rs12980430, p_min = 3.66e-07	
ZBTB45	chr19:59024896-59053079	BPD, rs28821001, p_min = 3.51e-06	gF, rs12980430, p_min = 3.66e-07	
CSE1L	chr20:47662782-47713497	Education, rs6122735, p_min = 9.672e-08	gF, rs6095417, p_min = 4.342e-16	
CACNA1I	chr22:39966757-40085740	SCZ, rs5995756, p_min = 2.836e-11	Education, rs5995757, p_min = 1.022e-07	gF, rs5757730, p_min = 6.545e-08
L3MBTL2	chr22:41601312-41627275	SCZ, rs9607771, p_min = 5.174e-09	Neuroticismism, rs2273085, p_min = 5.115e-07	
CENPM	chr22:42334724-42343168	SCZ, rs6002655, p_min = 1.475e-09	gF, rs5758605, p_min = 2.045e-11	
LINC00634	chr22:42348190-42354946	SCZ, rs6002655, p_min = 1.475e-09	gF, rs5758605, p_min = 2.045e-11	
CYP2D7	chr22:42535602-42540576	SCZ, rs6002655, p_min = 1.475e-09	gF, rs5758605, p_min = 2.045e-11	

Column names: Gene - RefSeq gene names; coordinates - chromosome position (hg19); Trait 1 - trait name, rs id of selected in cojo-GCTA SNP, p-value of association with the Trait 1; Trait 2 - trait name, rs id of selected in cojo-GCTA SNP, p-value of association with the Trait 2; Trait 3 - trait name, rs id of selected in cojo-GCTA SNP, p-value of association with the Trait 3, etc. Markers selected for Trait1, Trait2 and Trait3 etc are LD – dependent.
Method – name of the method and reference, if a corresponding gene was selected in previous studies.
SCZ - schizophrenia; BPD - bipolar disoder; Alz - Alzheimer

Genes highlighted with bold, have genome-wide significant associations with 2+ traits

Trait4	Trait 5	Trait 6	Method
			MTAG, education/intelligence (Hill et al, 2019).
			MTAG, education/intelligence (Hill et al, 2019).
			MTAG, education/intelligence (Hill et al, 2019).
			MTAG, education/intelligence (Hill et al, 2019),
			MTAG, education/intelligence (Hill et al, 2019).
			MTAG, education/intelligence (Hill et al, 2019).
**Education121, rs2268894, p = 4.689e-09**			MTAG, education/intelligence (Hill et al, 2019).
			MTAG, education/intelligence (Hill et al, 2019).
			MTAG, education/intelligence (Hill et al, 2019).
			MTAG, education/intelligence (Hill et al, 2019), cFDR, SCZ/education (Le Hellard et al, 2017)
			MTAG, education/intelligence (Hill et al, 2019).
			MTAG, education/intelligence (Hill et al, 2019),cFDR, SCZ/education (Le Hellard et al, 2017)
			MTAG, education/intelligence (Hill et al, 2019), cFDR, SCZ/education (Le Hellard et al, 2017)
			MTAG, education/intelligence (Hill et al, 2019), cFDR, SCZ/education (Le Hellard et al, 2017)
			MTAG, education/intelligence (Hill et al, 2019).
			MTAG, education/intelligence (Hill et al, 2019).
			MTAG, education/intelligence (Hill et al, 2019).
			MTAG, education/intelligence (Hill et al, 2019).
			MTAG, education/intelligence (Hill et al, 2019).
			MTAG, education/intelligence (Hill et al, 2019).
Deprsym, rs9584855, p = 3.325e-06, +			
			MTAG, education/intelligence (Hill et al, 2019).
			MTAG, education/intelligence (Hill et al, 2019).
			MTAG, education/intelligence (Hill et al, 2019).
			MTAG, education/intelligence (Hill et al, 2019).
**Neuroticism, rs62057143, p = 1.762e-10, -**			MTAG, education/intelligence (Hill et al, 2019).
**Education, rs62100765, p = 1.126e-08, -**	**Deprsym, rs8099160, p = 2.683e-08, +**	**Neuroticism139, rs4632195, p = 4.573e-07**	MTAG, education/intelligence (Hill et al, 2019).
			MTAG, education/intelligence (Hill et al, 2019).
			MTAG, education/intelligence (Hill et al, 2019).

MTAG, education/intelligence (Hill et al, 2019).
MTAG, education/intelligence (Hill et al, 2019).

MTAG, education/intelligence (Hill et al, 2019).
MTAG, education/intelligence (Hill et al, 2019).

MTAG, education/intelligence (Hill et al, 2019).

MTAG, education/intelligence (Hill et al, 2019).
MTAG, education/intelligence (Hill et al, 2019).
MTAG, education/intelligence (Hill et al, 2019).

MTAG, education/intelligence (Hill et al, 2019).

cFDR, SCZ/education (Le Hellard et al, 2017)

MTAG, education/intelligence (Hill et al, 2019).
MTAG, education/intelligence (Hill et al, 2019).
MTAG, education/intelligence (Hill et al, 2019).

MTAG, education/intelligence (Hill et al, 2019).
MTAG, education/intelligence (Hill et al, 2019).
MTAG, education/intelligence (Hill et al, 2019).
MTAG, education/intelligence (Hill et al, 2019).
MTAG, education/intelligence (Hill et al, 2019).
MTAG, education/intelligence (Hill et al, 2019).
MTAG, education/intelligence (Hill et al, 2019).
MTAG, education/intelligence (Hill et al, 2019).
MTAG, education/intelligence (Hill et al, 2019).

	MTAG, education/intelligence (Hill et al, 2019).
	MTAG, education/intelligence (Hill et al, 2019).
	MTAG, education/intelligence (Hill et al, 2019).
	MTAG, education/intelligence (Hill et al, 2019).
	MTAG, education/intelligence (Hill et al, 2019).
**gF, rs62037363, p_min = 1.089e-18**	MTAG, education/intelligence (Hill et al, 2019).
**gF, rs62037363, p_min = 1.089e-18**	MTAG, education/intelligence (Hill et al, 2019).
**gF, rs62037363, p_min = 1.089e-18**	
**gF, rs62037363, p_min = 1.089e-18**	MTAG, education/intelligence (Hill et al, 2019).
**gF, rs62037363, p_min = 1.089e-18**	
**gF, rs113434679, p_min = 1.441e-11**	MTAG, education/intelligence (Hill et al, 2019).
**gF, rs113434679, p_min = 1.441e-11**	MTAG, education/intelligence (Hill et al, 2019).
	MTAG, education/intelligence (Hill et al, 2019).
	MTAG, education/intelligence (Hill et al, 2019).
	MTAG, education/intelligence (Hill et al, 2019).
