# Supplementary material for: Identification of pleiotropy at the gene level between psychiatric disorders and related traits
Source: Transl Psychiatry. 2021 Jul 29;11:410. doi: 10.1038/s41398-021-01530-4 (PMC8322263; doi:10.1038/s41398-021-01530-4)
Supplement: Supplementary file 15 — Supplementary Table 5 [file 41398_2021_1530_MOESM15_ESM.pdf]

Gene	Coordinates	Trait1	Trait2	Trait3
**SNP-based analysis**				
Block(FOXO6;LOC101929901)	chr1:41827602-41849263	Education, rs12029493, p = 1.177e-06, +	gF, rs61774752, p = 5.076e-08, -	
NEGR1	**chr1:71868624-72748277**	**Education, rs12137231, p = 3.856e-07, +; rs669**	**gF, rs1620977, p = 1.282e-12, +**	**SCZ, rs7531932, p = 2.881e-06, +**
ZEB2	chr2:145141941-145277958	Education, rs12614546, p = 1.289e-06, +	SCZ, rs35015447, p = 1.291e-06, +	
ATP2B2	chr3:10365706-10547374	BPD, rs735931, p = 1.298e-06, +	SCZ, rs9879311, p = 2.771e-06, +	
FOXP1	chr3:71003864-71633140	Education, rs11714337, p = 4.995e-07, +	SCZ, rs6803008, p = 3.288e-07, -; rs7372960, p	gF, rs9847154, p = 8.805e-07, +
BBX	chr3:107241782-107530176	gF, rs11710737, p = 2.801e-06, -	ADHD, rs7634587, p = 3.481e-06, +	Education, rs7635655, p = 1.771e-06, -
SOX2-OT	chr3:180774467-181460013	SCZ, rs17537703, p = 5.375e-07, -	gF, rs2216428, p = 5.349e-06, -	
MAML3	chr4:140637545-141075233	Education, rs12331577, p = 7.679e-07, +; rs4863	Depressive symptoms, rs12504594, p = 5.239e-(	Wellbeing, rs17005492, p = 2.012e-06, +
LINC00461	**chr5:87836596-87980620**	**gF, rs10514301, p = 8.96e-12, +**	**ADHD, rs4916723, p = 1.576e-08, -**	**Education, rs6882046, p = 7.921e-14, -**
MEF2C-AS1	**chr5:88179146-88763398**	**SCZ, rs16867576, p = 1.358e-08, +**	**Alz, rs190982, p = 2.547e-06, -**	**ADHD, rs304132, p = 4.225e-08, -**
LOC101927078	chr5:113783114-114109110	gF, rs10519387, p = 1.995e-06, +	Education, rs13165806, p = 1.188e-06, +; rs2553520, p = 1.256e-08, +	
MAD1L1	chr7:1855427-2272583	BPD, rs10950456, p = 2.376e-06, +	ADHD, rs13234909, p = 2.814e-06, +	SCZ, rs58120505, p = 6.426e-14, +
SP4	chr7:21467651-21554440	gF, rs39302, p = 8.402e-08, -	BPD, rs6954854, p = 4.107e-06, -	SCZ, rs73060317, p = 6.604e-07, +
PDE1C	chr7:31823125-32467836	Education, rs57877670, p = 1.935e-06, +	gF, rs9648380, p = 2.616e-06, +	
POU6F2	chr7:39017608-39504390	gF, rs28599716, p = 1.145e-07, -	SCZ, rs7786896, p = 2.383e-06, +	
EXOC4	chr7:132937822-133750513	gF, rs10258052, p = 1.002e-11, +	Education, rs17167210, p = 2.791e-06, -	AUT, rs6467494, p = 1.425e-06, +
ELAVL2	chr9:23690102-23826063	AUT, rs1329044, p = 5.277e-06, -	gF, rs3793574, p = 1.484e-06, -	
CAMK1D	chr10:12391541-12877545	Education, rs10752262, p = 7.562e-08, +; rs7087	gF, rs61663121, p = 2.909e-06, -	
JMJD1C	**chr10:64926980-65281835**	**gF, rs10761765, p = 1.916e-08, -**	**Education, rs7896518, p = 2.25e-08, -**	
SORCS3	chr10:106400858-107025000	gF, rs11192193, p = 1.609e-06, -	ADHD, rs12265655, p = 1.34e-08, +	Depressive symptoms, rs7074335, p = 4.57e-(
CACNA1C	**chr12:2162415-2807115**	**BPD, rs10744560, p = 2.918e-09, +**	**SCZ, rs12823424, p = 5.473e-09, +; rs2007044, p = 2.625e-17,**	
SEMA6D	chr15:47476402-48066420	Education, rs281302, p = 6.107e-08, -	ADHD, rs281324, p = 2.678e-08, -; rs977667, p = 5.838e-08, +	
EFL1	chr15:82422560-82555104	gF, rs12439619, p = 3.384e-08, -	Alz, rs905450, p = 2.819e-06, -	
Gene-based analysis				
LOC101929901	chr1:41840675-41841341	Education, rs11209970, p_min = 3.124e-07	gF, rs1892419, p_min = 6.172e-16	
KDM4A-AS1	**chr1:44165408-44173012**	**SCZ, rs11210892, p_min = 4.97e-10**	**ADHD, rs112984125, p_min = 3.581e-13**	**Education, rs12410444, p_min = 2.136e-11**
LOC101929592	**chr1:44175075-44193014**	**SCZ, rs11210892, p_min = 4.97e-10**	**ADHD, rs112984125, p_min = 3.581e-13**	**Education, rs12410444, p_min = 2.136e-11**
AKT3	chr1:243651534-244006584	SCZ, rs13376709, p_min = 7.516e-08	Education, rs4614242, p_min = 5.255e-06	
VRK2	chr2:58134785-58387055	SCZ, rs11682175, p_min = 2.543e-12	Neuroticism, rs62140779, p_min = 1.157e-06	
C2orf74	chr2:61372202-61391964	Education, rs10496091, p_min = 5.611e-06	gF, rs7608965, p_min = 1.342e-06	
SLC4A10	**chr2:162480844-162841786**	**Hippocampus, rs2268894, p_min = 2.213e-07**	**Education, rs2268894, p_min = 4.689e-09**	**gF, rs2284871, p_min = 2.624e-12**
ARIH2	**chr3:48956252-49022971**	**ADHD, rs62262061, p_min = 9.691e-07**	**Education, rs13090388, p_min = 4.287e-23**	**gF, rs7646366, p_min = 8.27e-18**
MST1	**chr3:49721379-49726196**	**ADHD, rs62262061, p_min = 9.691e-07**	**Education, rs2883059, p_min = 7.149e-25**	**gF, rs9873183, p_min = 4.551e-20**
RNF123	**chr3:49726931-49758962**	**ADHD, rs62262061, p_min = 9.691e-07**	**Education, rs2883059, p_min = 7.149e-25**	**gF, rs9873183, p_min = 4.551e-20**
RBM6	**chr3:49977476-50114685**	**ADHD, rs62262061, p_min = 9.691e-07**	**Education, rs2883059, p_min = 7.149e-25**	**gF, rs9873183, p_min = 4.551e-20**
NDUFAF2	**chr5:60240955-60448864**	**SCZ, rs4391122, p_min = 1.734e-13**	**Education, rs61160187, p_min = 5.929e-13**	**gF, rs34627, p_min = 2.251e-09**
SMIM15-AS1	**chr5:60458142-60527907**	**SCZ, rs4391122, p_min = 1.734e-13**	**Education, rs61160187, p_min = 5.929e-13**	**gF, rs34627, p_min = 2.251e-09**
MIR9.2	**chr5:87962670-87962757**	**ADHD, rs304132, p_min = 4.225e-08**	**Education, rs6882046, p_min = 7.921e-14**	**Neuroticism, rs6882046, p_min = 1.603e-07**
HBEGF	**chr5:139712427-139726188**	**SCZ, rs13168670, p_min = 4.277e-07**	**Alz, rs2878896, p_min = 9.219e-08**	**gF, rs4463213, p_min = 7.96e-10**
HIST1H2BD	**chr6:26158348-26171576**	**SCZ, rs34661691, p_min = 1.769e-24**	**Education, rs766406, p_min = 1.888e-08**	**Depressive symptoms, rs3799380, p_min = 3**
ZNF204P	**chr6:27325601-27343153**	**SCZ, rs7746199, p_min = 3.35e-26**	**Depressive symptoms, rs853679, p_min = 6.62**	**Neuroticism, rs9468186, p_min = 2.321e-07**
ZNF391	**chr6:27342413-27371686**	**SCZ, rs7746199, p_min = 3.35e-26**	**Depressive symptoms, rs853679, p_min = 6.62**	**Neuroticism, rs9468186, p_min = 2.321e-07**
ZNF184	**chr6:27371790-27440897**	**SCZ, rs7746199, p_min = 3.35e-26**	**Depressive symptoms, rs853679, p_min = 6.62**	**Neuroticism, rs9468186, p_min = 2.321e-07**

ZSCAN31	**chr6:28292514-28324048**	**SCZ, rs7766356, p_min = 7.679e-22**	**gF, rs28551159, p_min = 5.544e-11**	
PSMB8	**chr6:32808493-32812712**	**SCZ, rs116385615, p_min = 5.38e-20**	**Alz, rs111805310, p_min = 8.413e-09**	**Education, rs9274660, p_min = 5.145e-06**
ATF6B	**chr6:32083044-32096017**	**SCZ, rs116385615, p_min = 5.38e-20**	**Alz, rs77212406, p_min = 6.835e-09**	**Education, rs9267677, p_min = 4.067e-06**
TAP2	chr6:32789609-32806547	SCZ, rs116385615, p_min = 5.38e-20	Alz, rs115674098, p_min = 6.714e-07	
C2	chr6:31865561-31913451	SCZ, rs116385615, p_min = 5.38e-20	Education, rs9267677, p_min = 4.067e-06	gF, rs2442727, p_min = 4.573e-06
C2-AS1	chr6:31902250-31909501	SCZ, rs116385615, p_min = 5.38e-20	Education, rs9267677, p_min = 4.067e-06	gF, rs2442727, p_min = 4.573e-06
TNXB	**chr6:32008931-32077151**	**SCZ, rs116385615, p_min = 5.38e-20**	**Alz, rs77212406, p_min = 6.835e-09**	**Education, rs9267677, p_min = 4.067e-06**
KMT2E	chr7:104654636-104754532	SCZ, rs6466055, p_min = 2.463e-09	BPD, rs118089279, p_min = 8.294e-07	Education, rs4073894, p_min = 4.125e-06
MIR1183	chr7:21510675-21510764	SCZ, rs73060317, p_min = 6.604e-07	gF, rs39302, p_min = 8.402e-08	
FOXP2	chr7:113726364-114333827	ADHD, rs10262192, p_min = 2.887e-08	Education, rs10953766, p_min = 5.28e-08	
MIR3666	chr7:114293399-114293510	ADHD, rs10262192, p_min = 2.887e-08	Education, rs10953766, p_min = 5.28e-08	
MSRA	chr8:9911778-10286401	SCZ, rs2048656, p_min = 1.451e-07	Neuroticism, rs2572431, p_min = 4.197e-16	
LOC105378330	**chr10:65190334-65190962**	**Education, rs1396967, p_min = 2.901e-09**	**gF, rs10761765, p_min = 1.916e-08**	
MACROD1	chr11:63766029-63933585	SCZ, rs4963432, p_min = 1.312e-06	BPD, rs4980532, p_min = 8.953e-07	gF, rs683686, p_min = 1.535e-07
LOC100507431	**chr11:130714087-130732005**	**SCZ, rs10791097, p_min = 2.876e-12**	**Education, rs11222416, p_min = 1.167e-08**	
SBNO1	**chr12:123773655-123849756**	**SCZ, rs2102949, p_min = 2.416e-14**	**Education, rs9739070, p_min = 3.954e-16**	**gF, rs2030401, p_min = 2.492e-07**
SAXO2	**chr15:82555151-82577267**	**SCZ, rs783540, p_min = 5.857e-08**	**Education, rs28420834, p_min = 1.549e-09**	**gF, rs12439619, p_min = 3.384e-08**
CPEB1	**chr15:83211950-83316767**	**SCZ, rs783540, p_min = 5.857e-08**	**Education, rs28420834, p_min = 1.549e-09**	**gF, rs12439619, p_min = 3.384e-08**
GOSR2	chr17:45000482-45044366	Education, rs7220777, p_min = 4.146e-06	gF, rs17668126, p_min = 1.01e-06	
NCAN	**chr19:19322781-19363061**	**SCZ, rs2905426, p_min = 6.921e-09**	**BPD, rs111444407, p_min = 2.404e-10**	**Education, rs12981405, p_min = 4.467e-06**
HAPLN4	**chr19:19366451-19373596**	**SCZ, rs2905426, p_min = 6.921e-09**	**BPD, rs111444407, p_min = 2.404e-10**	**Education, rs12981405, p_min = 4.467e-06**
MAU2	**chr19:19431629-19469563**	**SCZ, rs2905426, p_min = 6.921e-09**	**BPD, rs111444407, p_min = 2.404e-10**	**Education, rs12981405, p_min = 4.467e-06**
YJEFN3	**chr19:19639669-19648393**	**SCZ, rs2905426, p_min = 6.921e-09**	**BPD, rs111444407, p_min = 2.404e-10**	**Education, rs12981405, p_min = 4.467e-06**
CILP2	**chr19:19649073-19657468**	**SCZ, rs2905426, p_min = 6.921e-09**	**BPD, rs111444407, p_min = 2.404e-10**	**Education, rs12981405, p_min = 4.467e-06**
MGAT3	**chr22:39853324-39888199**	**SCZ, rs5995756, p_min = 2.836e-11**	**Education, rs5995757, p_min = 1.022e-07**	**gF, rs3959642, p_min = 2.582e-08**
RANGAP1	**chr22:41640780-41698217**	**SCZ, rs9607771, p_min = 5.174e-09**	**Neuroticism, rs2273085, p_min = 5.115e-07**	**gF, rs12170228, p_min = 4.491e-08**
ZC3H7B	**chr22:41697506-41756151**	**SCZ, rs9607771, p_min = 5.174e-09**	**Neuroticism, rs2273085, p_min = 5.115e-07**	**gF, rs12170228, p_min = 4.491e-08**
SLC25A17	**chr22:41165633-41215403**	**SCZ, rs133047, p_min = 9.728e-10**	**BPD, rs138321, p_min = 4.692e-09**	
MIR4766	**chr22:41209886-41209962**	**SCZ, rs9607771, p_min = 5.174e-09**	**BPD, rs138321, p_min = 4.692e-09**	

Column names: Gene - RefSeq gene names; coordinates - chromosome position (hg19); Trait 1 - trait name, rs id of selected in cojo-GCTA SNP, p-value of association with the Trait 1; Trait 2 - trait name, rs id of selected in cojo-GCTA SNP, p-value of association with the Trait 2; Trait 3 - trait name, rs id of selected in cojo-GCTA SNP, p-value of association with the Trait 3, etc. r2 - rs ids and r2 value for the SNPs. R2 is calculated based on 1000 Genomes, EUR sample. Markers highligthed with blue are used for LD calculation,

SCZ - schizophrenia; BPD - bipolar disoder; Alz - Alzheimer

Genes highlighted with bold, have genome-wide significant associations with 2+ traits

Trait 4	r2	Method
		MTAG, education/intelligence (Hill et al, 2019)
	**rs12137231,rs1620977: r2 = 0.36**	MTAG, education/intelligence (Hill et al, 2019)
		cFDR, SCZ/education (Le Hellard et al, 2017)
		MTAG, education/intelligence (Hill et al, 2019), cFDR, SCZ/education (Le Hellard et al, 2017)
	rs11710737,rs7634587: r2 = 0.47	
		MTAG, education/intelligence (Hill et al, 2019)
**Neurotic52, rs6882046, p = 1.603e-07**	**rs6882046, rs6882046: r2=1**	MTAG, education/intelligence (Hill et al, 2019)
Education, rs304138, p = 2.643e-10, -		MTAG, education/intelligence (Hill et al, 2019)
Education, rs62444881, p = 4.855e-07, +		
		MTAG, education/intelligence (Hill et al, 2019)
		MTAG, education/intelligence (Hill et al, 2019)
		MTAG, education/intelligence (Hill et al, 2019)
		MTAG, education/intelligence (Hill et al, 2019)
	rs61663121,rs7087145: r2 = 0.26	
		MTAG, education/intelligence (Hill et al, 2019)
07, +		MTAG, education/intelligence (Hill et al, 2019)
**gF, rs2819336, p_min = 8.442e-11**		MTAG, education/intelligence (Hill et al, 2019)
**gF, rs2819336, p_min = 8.442e-11**		
		cFDR, SCZ/education (Le Hellard et al, 2017)
		MTAG, education/intelligence (Hill et al, 2019)
		MTAG, education/intelligence (Hill et al, 2019)
		MTAG, education/intelligence (Hill et al, 2019)
		MTAG, education/intelligence (Hill et al, 2019)
		MTAG, education/intelligence (Hill et al, 2019)
		MTAG, education/intelligence (Hill et al, 2019)
**gF, rs42850, p_min = 1.691e-12**		
		MTAG, education/intelligence (Hill et al, 2019)
**Neuroticism, rs6904071, p_min = 7.587e-07**	gF, rs9379900, p_min = 2.317e-14	MTAG, education/intelligence (Hill et al, 2019)
**gF, rs9379900, p_min = 2.317e-14**		MTAG, education/intelligence (Hill et al, 2019)
**gF, rs9379900, p_min = 2.317e-14**		MTAG, education/intelligence (Hill et al, 2019)
**gF, rs9379900, p_min = 2.317e-14**		MTAG, education/intelligence (Hill et al, 2019)

	MTAG, education/intelligence (Hill et al, 2019)
**gF, rs2442727, p_min = 4.573e-06**	
gF, rs1144, p_min = 1.008e-08	MTAG, education/intelligence (Hill et al, 2019), cFDR, SCZ/education (Le Hellard et al, 2017)
	MTAG, education/intelligence (Hill et al, 2019)
	MTAG, education/intelligence (Hill et al, 2019), cFDR, SCZ/education (Le Hellard et al, 2017)
	MTAG, education/intelligence (Hill et al, 2019)
