# Supplementary material for: Identification of pleiotropy at the gene level between psychiatric disorders and related traits
Source: Transl Psychiatry. 2021 Jul 29;11:410. doi: 10.1038/s41398-021-01530-4 (PMC8322263; doi:10.1038/s41398-021-01530-4)
Supplement: Supplementary file 16 — Supplementary Table 6 [file 41398_2021_1530_MOESM16_ESM.pdf]

Identified by	Independent signals	*Gene and localisation*	Traits	Function	Relevant notes from literature	Reference	Previous finding with methods using several GWASs
Association overlap	Same block	*RERE (Arginine-Glutamic Acid Dipeptide Repeats) 1:8,412,463-8,877,699*	SCZ, Education	The encoded protein co-localizes with a transcription factor in the nucleus, and its overexpression triggers apoptosis. May function as an epigenetic regular of gene expression. (PMID: 27762073)	Patients with neurodevelopmental disorder with or without anomalies of the brain, eye, or heart.	OMIM	
Gene score	Different blocks	*COL16A1 (Collagen Type XVI Alpha 1 Chain) 1:32,117,847-32,169,768*	Education, gF	Involved in mediating cell attachment and inducing integrin-mediated cellular reactions, such as cell spreading and alterations in cell morphology.			
Association overlap	Same block	*CACNA1E (Calcium Voltage-Gated Channel Subunit Alpha1 E) 1:181,452,685-181,775,921*	gF, Neuroticism	Voltage-sensitive calcium channels (VSCC) mediate the entry of calcium ions into excitable cells	pathogenic variants in CACNA1E as a cause of developmental and epileptic encephalopathies	28573794, 30343943	
Gene score	Same block	*LRRN2 (leucine rich repeat neuronal 2) 1:204,586-204,654*	SCZ, Education	Involved in the development and maintenance of excitatory synapses in the vertebrate nervous system. Regulates surface expression of AMPA receptors and instructs the development of functional glutamate release sites.	Causes neurodegeneration and exacerbated neuroinflammation in the brain of mouse model.	29800472, 30368241	MTAG, education/intelligence (Hill et al, 2019)
Gene score	Same block	*BRE-AS1 (BABAM2 Antisense RNA 1) 2:28,112,322-28,113,981*	SCZ , BPD	An RNA Gene, affiliated with the lncRNA class. Diseases associated with it include Chromophobe Renal Cell Carcinoma.			
Association overlap	Same block	*RBKS (ribokinase) 2:204,586,302-204,654,597*	SCZ, BPD	Catalyzes the phosphorylation of ribose at O-5 in a reaction requiring ATP and magnesium. The resulting D-ribose-5-phosphate can then be used either for sythesis of nucleotides, histidine, and tryptophan.			
Gene score	Different blocks	*SFXN5 (Sideroflexin 5) 2:73,169,164-73,298,965*	SCZ, gF	Gene Ontology annotations related to this gene include cation transmembrane transporter activity and citrate transmembrane transporter activity. An important paralog of this gene is SFXN2.	Northern blot analysis revealed a single SFXN5 transcript of approximately 4.3 kb, which was primarily expressed in the brain.	12039050	
Association overlap	Different blocks	*CTNNA2 (catenin alpha 2) 2:79,740,059-80,875,988*	Education, gF	May function as a linker between cadherin adhesion receptors and the cytoskeleton to regulate cell-cell adhesion and differentiation in the nervous system (By similarity). Required for proper regulation of cortical neuronal migration and neurite growth (PubMed:30013181).	*CTNNA2* as the first catenin family member with biallelic mutations in humans, uncover a key factor involved in ARP2/3 repression in neurons	30013181, 28250917	
Association overlap	Different blocks	*LRP1B (LDL Receptor Related Protein 1B) 2:140,988,995-142,889,270*	Education, gF	LRP1B is a putative tumor suppressor and a member of the low-density lipoprotein (LDL) receptor family. The LDL receptor family have roles related to clearance of extracellular ligand and are proposed to be involved in extracellular signal transduction.	LRP1B has the significant association with Alz susceptibility in Caribbean Hispanic individuals.	26621834	
Gene score	Different blocks	*TEX41 (Testis Expressed 41) 2:145,425,533-145,834,291*	ADHD, Education	TEX41 is an RNA Gene, and is affiliated with the lncRNA class. Diseases associated with TEX41 include Aortic Valve Disease 1.	De novo triplication in a boy with global developmental delay, cognitive impairment, multiple congenital anomalies and behavior problems	26705424	
Association overlap	Different blocks	*ZNF385B (Zinc Finger Protein 385B) 2:180,306,710-180,726,232*	SCZ, gF	Diseases associated with ZNF385B include Long Qt Syndrome 1. Gene Ontology (GO) annotations related to this gene include nucleic acid binding and p53 binding. An important paralog of this gene is ZNF385D.			
Gene score	Different blocks	*SATB2 (SATB Homeobox 2) 2:200,134,222-200,335,989*	SCZ, gF	Acts as a docking site for several chromatin remodeling enzymes and also by recruiting corepressors (HDACs) or coactivators (HATs) directly to promoters and enhancers. Required for the initiation of the upper-layer neurons (UL1) specific genetic program and for the inactivation of deep-layer neurons (DL) and UL2 specific gene.	Data suggest that Satb2 functions as a repressor of Ctip2 and regulatory determinant of corticocortical connections in the developing cerebral cortex.	18255030	
Association overlap	Different blocks	*FHIT (fragile histidine triad) 3:59,733,003-61,237,135*	SCZ , MDD	Modulates transcriptional activation by CTNNB1 and thereby contributes to regulate the expression of genes essential for cell proliferation and survival, such as CCND1 and BIRC5. Plays a role in the induction of apoptosis via SRC and AKT1 signaling pathways.	Rare CNVs in the *FHIT* gene have been reported in autism	17363630	

Gene score	Same block	*FXR1 (Fragile X Mental Retardation, Autosomal Homolog 1)* 3:180,630,233-180,700,539	SCZ, gF	RNA-binding protein required for embryonic and postnatal development of muscle tissue. May regulate intracellular transport and local translation of certain mRNAs (By similarity).	Premutation CGG repeat expansions (55–200 CGG repeats; preCGG) within the *FMR1* gene cause fragile X-associated tremor/ataxia syndrome.	22466801	
Gene score	Different blocks	*LINC01378 (Long Intergenic Non-Protein Coding RNA 1378)* 4:118,349,553-118,610,258	SCZ, BPD	An RNA Gene, and is affiliated with the lncRNA class			
Association overlap	Different blocks	*GPM6A (glycoprotein M6A) 4:176,554,087-176,923,842*	SCZ, Education	Involved in neuronal differentiation, including differentiation and migration of neuronal stem cells. Plays a role in neuronal plasticity and is involved in neurite and filopodia outgrowth, filopodia motility and probably synapse formation.	Mutation can cause claustrophobia in mice	23632458	
Gene score	Same block	*EFNA5 (Ephrin A5) 5:106,712,589-107,006,596*	Education, gF	A member of the ephrin gene family; prevents axon bundling in cocultures of cortical neurons with astrocytes, a model of late stage nervous system development and differentiation.	The third lowest p-value in OCD GWAS (2013) with for the SNP rs26728 (p=4.75×10-6), located within an intron of *EFNA5*	22889921	
Gene score	Same block	*MAN2A1 (mannosidase alpha class 2A member 1) 5:5:109,025,066-109,205,326*	SCZ, Education	Catalyzes the first committed step in the biosynthesis of complex N-glycans. It controls conversion of high mannose to complex N-glycans; the final hydrolytic step in the N-glycan maturation pathway.	SNPs of the *MAN2A1* were associated with exploratory eye movement abnormalities; dysfunction seems to be specific to schizophrenia	26242244	MTAG, education/intelligence (Hill et al, 2019)
Association overlap	Same block	*JADE2 (jade family PHD finger 2) 5:133,860,065-133,918,920*	Education, BPD	Component of the HBO1 complex which has a histone H4-specific acetyltransferase activity, a reduced activity toward histone H3 and is responsible for the bulk of histone H4 acetylation in vivo		16387653	
Association overlap	Different blocks	*ATXN1 (Ataxin 1) 6:16,299,342-16,761,721*	Education, gF	The autosomal dominant cerebellar ataxias are a heterogeneous group of neurodegenerative disorders characterized by progressive degeneration of the cerebellum, brain stem and spinal cord.	Deletions in chromosome 6p22.3-p24.3, including ATXN1, are associated with developmental delay and autism spectrum disorders	28288114, 22480366	
Gene score	Different blocks	*EYS (Eyes Shut Homolog) 6:64,429,875-66,417,118*	SCZ, Education	The product of this gene contains multiple epidermal growth factor (EGF)-like and LamG domains. The protein is expressed in the photoreceptor layer of the retina, and the gene is mutated in autosomal recessive retinitis pigmentosa.			
Gene score	Same block	*ZMIZ2 (Zinc Finger MIZ-Type Containing 2) 7:44,788,164-44,809,479*	Education, gF	Increases ligand-dependent transcriptional activity of AR and other nuclear hormone receptors.			
Gene score	Same block	*CLU (clusterin) 8:27,454,433-27,472,328*	SCZ, Alzheimer	Extracellular chaperone that prevents aggregation of non-native proteins.	SNP assoctition with reduced risk for mild cognitive impariment in diabetes	1585460, 27516739, 28701379	
Association overlap	Different blocks	*DLGAP2 (DLG associated protein 2) 8:687,604-1,656,642*	SCZ, ADHD	May play a role in the molecular organization of synapses and neuronal cell signaling. Could be an adapter protein linking ion channel to the subsynaptic cytoskeleton.	Deleted in autism.	20531469	
Association overlap	Different blocks	*EXT1 (Exostosin Glycosyltransferase 1) 8:118,811,601-119,124,058*	gF, AUT	This gene encodes an endoplasmic reticulum-resident type II transmembrane glycosyltransferase involved in the chain elongation step of heparan sulfate biosynthesis. Mutations in this gene cause the type I form of multiple exostoses.	Removal of neuronal heparan sulfates by conditional deletion of the *Ext1* gene in postnatal neurons of amyloid model APP/PS1 mice led to a reduction in both Aβ oligomerization and the deposition of amyloid plaques. It has implications for Alz pathogenesis.	27030596	
Association overlap	Different blocks	*GLIS3 (GLIS family zinc finger 3) 9:3,824,127-4,300,035*	SCZ, Neuroticism	Acts as both a repressor and activator of transcription.	--		
Association overlap	Different blocks	*ASTN2 (astrotactin 2) 9:119,187,503-120,177,317*	BPD, Autism	Mediates recycling of the neuronal cell adhesion molecule ASTN1 to the anterior pole of the cell membrane in migrating neurons. ASTN2 modulates synaptic strength by trafficking and degradation.	Rare variants implicated in intellectual disablities, ADHD, autism and schizophrenia.	24381304, 18940311, 30242134	

Gene score	Same block	*TMEM245 (Transmembrane Protein 245) 9:111,777,414-111,882,225*	Education, gF	TMEM245 (Transmembrane Protein 245) is a Protein Coding gene.	SNP rs523340 within the *TMEM245* is associated with negative symptoms in SCZ, information processing speed	23382809	
Association overlap	Different blocks	*TEAD1 (TEA domain transcription factor 1) 11:12,695,968-12,966,284*	Education, Wellbeing	Transcription factor which plays a key role in the Hippo signaling pathway, a pathway involved in organ size control and tumor suppression by restricting proliferation and promoting apoptosis.	Mutations in Sveinsson chorioretinal atrophy (autosomal dominant eye disorder) and non X-linked Aicardi syndrome (congenital neurodevelopment disorder with mental retardation).	26091538	MTAG, education/intelligence (Hill et al, 2019)
Association overlap	Different blocks	*LRRC4C (Leucine Rich Repeat Containing 4C) 11:40,135,750-41,481,186*	Anorexia, gF	May promote neurite outgrowth of developing thalamic neurons	A 7q31.33q32.1 microdeletion including LRRC4 and GRM8 is associated with severe intellectual disability and characteristics of autism	doi:10.1038/hgv.2017.1	
Association overlap	Different blocks	*TENM4 (Teneurin Transmembrane Protein 4) 11:78,364,327-79,151,695*	BPD, gF	The protein encoded by this gene plays a role in establishing proper neuronal connectivity during development. Defects in this gene have been associated with hereditary essential tremor-5.	Missense mutations in *TENM4* , a regulator of axon guidance and central myelination, cause essential tremor	26188006, 30745909	
Gene score	Same block	*KCNC2 (Potassium Voltage-Gated Channel Subfamily C Member 2) 12:75,433,857-*	Education, Neuroticism	Voltage-gated potassium channel that mediates transmembrane potassium transport in excitable membranes, primarily in the brain. Contributes to the regulation of the fast action potential repolarization and in sustained high-frequency firing in neurons of the central nervous system. Channel properties	Deletion of exons 3–5 of the KCNC2 gene, was present in three affected members of the family with neurodevelopmental delay and ataxia.	23475819	
Association overlap	Different blocks	*MIPEPP3 (Mitochondrial Intermediate Peptidase Pseudogene 3) 13:21,872,263-21,967,061*	SCZ, gF				
Gene score	Same block	*CKB (Creatine Kinase B) 14:103,985,994-103,989,196*	SCZ, gF	Reversibly catalyzes the transfer of phosphate between ATP and various phosphogens (e.g. creatine phosphate). One of three cytosolic isoforms of creatine kinase that is predominantly expressed in the brain			
Gene score	Same block	*TRMT61A (TRNA Methyltransferase 61A) 14:103,995,508-104,003,410*	SCZ, gF	Among its related pathways are tRNA processing and Gene Expression. Gene Ontology annotations related to this gene include tRNA (adenine-N1-)-methyltransferase activity.			
Gene score	Same block	*APOPT1 (Cytochrome C Oxidase Assembly Factor 8) 14:104,029,293-104,058,510*	SCZ, gF	This gene encodes a protein that localizes to the mitochondria, where it stimulates the release of cytochrome c, thereby promoting programmed cell death.	APOPT1 mutations were identified in patients with brain MRI pattern characterized by cavitating leukodystrophy	25175347	
Association overlap	Different blocks	*EFL1 (elongation factor like GTPase 1) 15:82,422-82,555*	Intelligence, Alzheimer	Involved in the biogenesis of the 60S ribosomal subunit and translational activation of ribosomes.	Mutations/deletions cause Shwachman-Diamond like syndrome - global development delay.	https://www.ncbi.nlm.nih.gov/books/NBK1756/	
Gene score	Different blocks	*SLCO3A1 (Solute Carrier Organic Anion Transporter Family Member 3A1) 15:92,396,937-92,715,665*	Education, gF	Mediates the Na(+)-independent transport of organic anions such as estrone-3-sulfate (PubMed:10873595). Mediates transport of prostaglandins (PG) E1 and E2, thyroxine (T4), deltorphin II, BQ-123 and vasopressin	associated with nicotine dependence; mediates inflammatory processes in intestinal epithelial cells through NF-κB transcription activation, resulting in a higher incidence of bowel perforation in Crorh disease patients.	22377092, 24945726	
Association overlap	Different blocks	*RBFOX1 (RNA binding fox-1 homolog 1) 16:6,069,131-7,763,340*	SCZ, Intelligence	RNA-binding protein that regulates alternative splicing events by binding to 5'-UGCAUGU-3' elements. Regulates alternative splicing of tissue-specific exons and of differentially spliced exons.	Implicated in autism and other neurodevelopmental disorders.	27481563	MTAG, education/intelligence (Hill et al, 2019)
Association overlap	Different blocks	*SNX29 (Sorting Nexin 29) 16:12,070,590-12,668,146*	Education, gF	Gene Ontology annotations related to this gene include microtubule motor activity and phosphatidylinositol binding.	SNX29 as additional association signals for autism in Han Chinese	31051333, 31647196	

Gene score	Different blocks	*CDH8 (Cadherin 8) 16:61,685,914-62,070,739*	ADHD, gF	Cadherins are calcium-dependent cell adhesion proteins. They preferentially interact with themselves in a homophilic manner in connecting cells; cadherins may thus contribute to the sorting of heterogeneous cell types.	Rare familial 16q21 microdeletions and expression analysis implicate *CDH8* in susceptibility to autism and leaning disability.	20972252	
Association overlap	Different blocks	*TCF4 (transcription factor 4) 18:52,889-53,303*	Neuroticism, SCZ, Depressive symptoms	Transcription factor that binds to the immunoglobulin enhancer Mu-E5/KE5-motif. Involved in the initiation of neuronal differentiation.	Mutations can cause of Pitt-Hopkins Syndrome (mental retardation) and Fuch's corneal dystrophy.		
Association overlap	Same block	*CDH2 (Cadherin 2) 18:25,530,926-25,757,410*	Education, gF	Encodes a classical cadherin and member of the cadherin superfamily. Corresponding protein plays a role in the establishment of left-right asymmetry, development of the nervous system and the formation of cartilage and bone.	Disruption of CDH2/N-Cadherin-Based Adherens Junctions Leads to Apoptosis of Ependymal Cells and Denudation of Brain Ventricular Walls	23965744	
Association overlap	Different blocks	*MACROD2 (Mono-ADP Ribosylhydrolase 2) 20:13,976,145-16,033,841*	BPD, gF	Gene Ontology annotations related to this gene include hydrolase activity, acting on glycosyl.	MACROD2 gene associated with autistic-like traits in a general population sample	PMC4320645	
Association overlap	Same block	*PHACTR3 (Phosphatase And Actin Regulator 3) 20:58,152,563-58,422,766*	SCZ, gF	Gene Ontology annotations related to this gene include actin binding and protein phosphatase 1 binding.			
Gene score	Same block	*SHANK3 (SH3 and multiple ankyrin repeat domains 3) 22:51,113-*	Education, gF	Major scaffold postsynaptic density protein which interacts with multiple proteins and complexes to orchestrate the dendritic spine and synapse formation, maturation and maintenance.	Mutations implicated in autism. Deletions implicated in Phelan-McDermid syndrome (psychiatric and neurological features)	25894671, 20531469	**MTAG, education/intelligence (Hill et al, 2019)**
**Columns:** Identified by - refers to the steps of analysis, where the gene was identified; Independent signals - independent signals belong to either clear different LD blocks, or different haplotypes; Gene and location - RefSeq gene name, full gene name and gene coordinates with chromosome name, start and end positions in hg19; Traits - names of traits in which the associated signals were selected; Function - gene function from UniProt database https://www.uniprot.org/ (except *); Relevant notes from literature - description of known gene link to mental disorders or relevant phenotypes; Reference - PMID id or link to the studies referred to in the previous column; Previous finding with methods using several GWASs - if a gene was already reported in methods using pleiotropy-based methods, then names of the method, phenotypes and reference are shown. Bold text indicates previously published phentoypes that are identical to phenotypes in our study.							
Genes highlighted with grey are located in the same genomic locus.							
