# Supplementary material for: Identification of pleiotropy at the gene level between psychiatric disorders and related traits
Source: Transl Psychiatry. 2021 Jul 29;11:410. doi: 10.1038/s41398-021-01530-4 (PMC8322263; doi:10.1038/s41398-021-01530-4)
Supplement: Supplementary file 17 — Supplementary Table 7 [file 41398_2021_1530_MOESM17_ESM.pdf]

Supplementary Table 7. Summary statistics for SNPs rs4388249 and rs1368357 from SCZ and Education GWASs.

**SNP**	rs4388249	rs1368357
**Position**	Chr5: 109,036,066	Chr5:109,189,130
**SCZ GWAS**		
Allele	T/C	T/C
OR	1.075	0.974
P-value	1.025×10^{-7}	0.020
Frequencies in cases/controls for some separate SCZ substudies		
Danish, aarh	0.1989/0.1712	0.3181/0.3541
German, boco	0.1940/0.1797	0.3248/0.3248
Swedish, s234	0.2004/0.1828	0.3177/0.3308
Norwegian, top8	0.1645/0.1849	0.3522/0.3364
PEIC, WTCCC2, pewb	0.1594/0.1493	0.3416/0.3244
**Education GWAS**		
Allele	T/C	T/C
Effective allele frequency	0.160	0.347
Beta	0.006	-0.012
P-value	0.063	3.375×10^{-6}
